# Gallium-68 and zirconium-89 complexes of DFO-analogs for molecular imaging applications using PET/CT

**DOI:** 10.1186/s41181-026-00509-0

**Published:** 2026-10-23

**Authors:** Robin A. J. Maradan, Barbora Neuzilova, Julia Kronberger, Irene Feiner, Xabier Guarrochena, Ayfer Y. Kılçar, Jan Ralis, Milos Petrik, Thomas L. Mindt

**Affiliations:** 1 Institute of Inorganic Chemistry, Faculty of Chemistry, University of Vienna, Josef-Holaubek-Platz 2 and Währinger Straße 42, Vienna, 1090 Austria; 2 Vienna Doctoral School in Chemistry, University of Vienna, Währinger Straße 42, Vienna, 1090 Austria; 3 Joint Applied Medicinal Radiochemistry Facility, University of Vienna, Währinger Straße 42, Vienna, 1090 Austria; 4 Medical University of Vienna, Spitalgasse 23, Vienna, 1090 Austria; 5 Institute of Molecular and Translational Medicine, Faculty of Medicine and Dentistry, Palacký University, Olomouc, Czech Republic; 6 Ludwig Boltzmann Institute Applied Diagnostic, General Hospital of Vienna, Vienna, Austria; 7 Institute of Nuclear Sciences, Ege University, Bornova-Izmir, 35100 Türkiye; 8 Nuclear Physics Institute, Czech Academy of Sciences, Husinec, Czechia; 9 Czech Advanced Technology and Research Institute, Palacký University, Olomouc, Czech Republic; 10 Institute of Molecular and Translational Medicine, University Hospital, Olomouc, Czech Republic

**Keywords:** Gallium-68, Zirconium-89, Hydroxamate chelators, DFO*, DFO*^sp^, OxoDFO*, Positron emission tomography (PET)

## Abstract

**Background:**

The β^+^-emitters gallium-68 (^68^Ga) and zirconium-89 (^89^Zr) are among the most widely used metallic radionuclides in nuclear medicine for diagnostic imaging by Positron Emission Tomography (PET). Inspired by nature’s bacterial siderophore desferrioxamine-B (DFO), a number of structurally related oligohydroxamate-based chelators have been studied for the complexation of both radiometals and for applications in nuclear medicine. We report here the solid phase (sp) synthesis of a new member of this family of acyclic chelators, DFO*^sp^ 3, and its comparison with DFO 1 (control) and DFO-analogs DFO* 2, and oxoDFO* 4 in vitro and in vivo.

**Results:**

DFO*^sp^ 3 and oxoDFO* 4 were synthesized efficiently on solid support by applying different protective group strategies. The [^68^Ga]Ga- and [^89^Zr]Zr-labeled chelators 1–4 were obtained with high radiochemical yield and purity. They were also found to be of favorable hydrophilicity (logP <-2,2), high stability in the media tested, and low protein binding (with the exception of [^68^Ga]Ga-DFO 5 and [^68^Ga]Ga-DFO* 6). In vivo, the radiometal complexes 5–12 showed good serum stability, fast clearance predominantly by renal excretion and low uptake in organs and tissues. In vivo PET/CT imaging confirmed the results of the ex vivo biodistribution studies, leading to the acquisition of high-quality PET/CT images with a low signal-to-noise ratio.

**Conclusion:**

The excellent in vitro and in vivo characteristics of [^68^Ga]Ga- and [^89^Zr]Zr-complexes of hydroxamate-based chelators 1–4 support their use in radiopharmaceutical development and diagnostic PET-imaging applications in nuclear medicine.

**Supplementary Information:**

The online version contains supplementary material available at https://doi.org/10.1186/s41181-026-00509-0.

## Background

The β^+^-emitters gallium-68 (^68^Ga) and zirconium-89 (^89^Zr) are among the most widely used metallic radionuclides in nuclear medicine for diagnostic imaging by Positron Emission Tomography (PET) (Brandt et al. 2018). According to their physical half-life (t½), they are usually paired with biological vectors of matching biological half-life. Therefore, ^68^Ga (t_½_=68 min, E_β+,max_=1.9 MeV, E_β+, ave_=0.89 MeV) is predominantly used in combination with small molecules and peptides with fast pharmacokinetics, whereas ^89^Zr (t_½_=78.4 h, E_β+,max_=897 keV, E_β+, ave_=396.9 keV) is employed for the radiolabeling of proteins, including antibodies, which display a long circulation time in the blood of several days (Feo et al. 2022, Martiniova et al. 2016). In terms of the latter, ^89^Zr-immunoPET has become an important tool for the in vivo visualization and quantification of cancer biomarkers (Dongen et al. 2021).

**Fig. 1:**
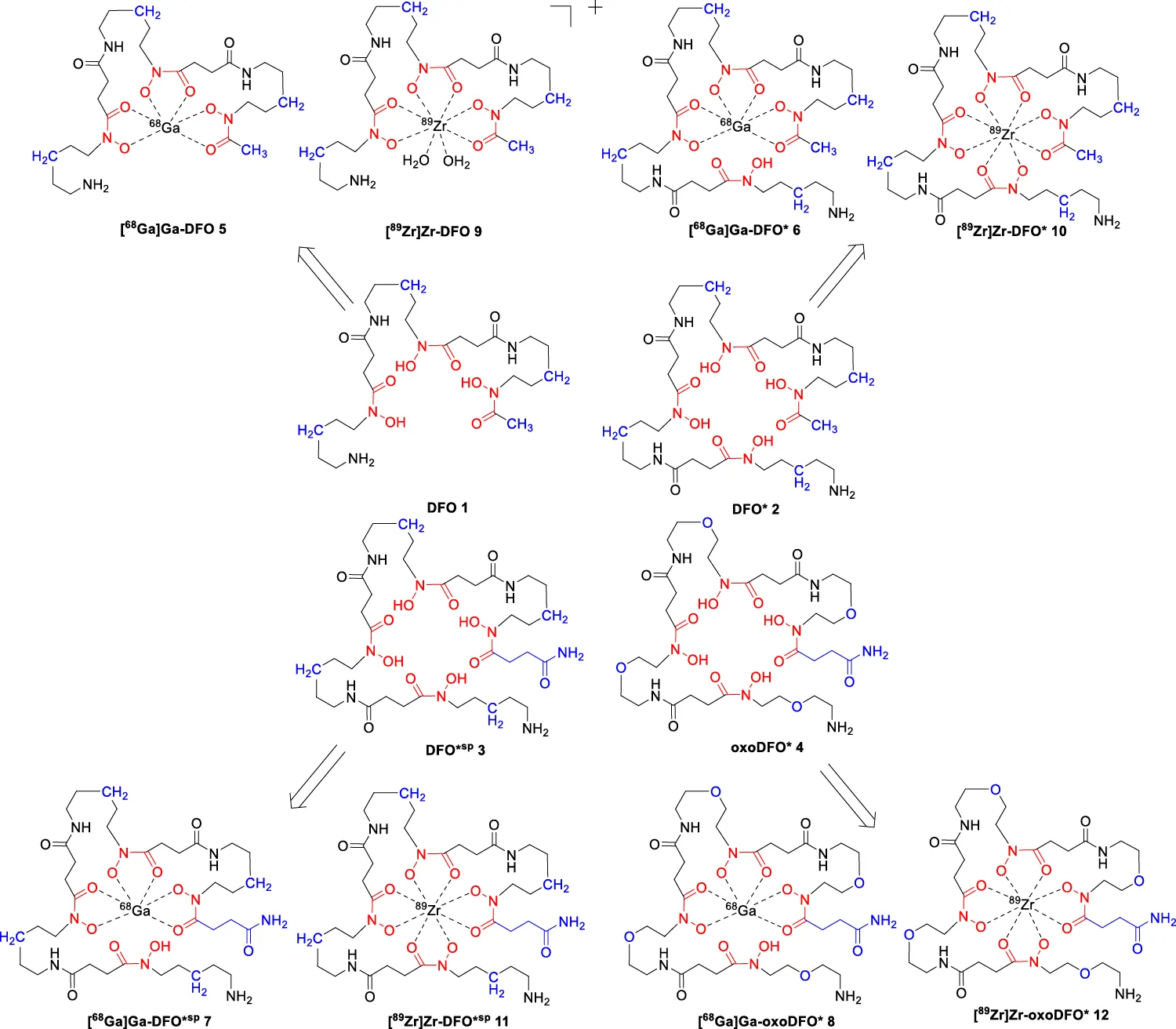
Structures of oligo hydroxamate-based, acyclic ^68^Ga- and ^89^Zr-chelators and the corresponding complexes used in this study. Hydroxamate groups for the complexation of the metal are shown in red and the structural differences of DFO-derivatives highlighted in blue

A prerequisite for the medical applications of ^68^Ga and ^89^Zr in nuclear medicine is the availability of chelators that form complexes of sufficient kinetic and thermodynamic stability in vitro and in vivo. Among the numerous chelators reported for ^68^Ga and ^89^Zr, some have been utilized for both radiometals, namely 1,4,7,10-tetraazacyclododecane-1,4,7,10-tetraacetic acid (DOTA) and desferroxamine-B (DFO 1, Fig. 1), each with its advantages and disadvantages (Feo et al. 2022, Martiniova et al. 2016, Petrik et al. 2021, Vugts et al. 2017, Pandya et al. 2017). Although radiolabeling of ^68^Ga and ^89^Zr with DOTA provides highly stable complexes, it requires elevated temperatures, reaction conditions unsuitable for sensitive biomolecules, e.g., antibodies. In the case of ^89^Zr, quantitative complexation with DOTA can only be achieved at a rather low pH (4.5) using [^89^Zr]ZrCl_4_, which is not yet commercially available (Lyashchenko et al. 2024). Conversely, hexadentate DFO 1 forms complexes with both metals at room temperature (RT) and neutral pH. [^68^Ga]Ga-DFO 5 has demonstrated its utility for applications in nuclear medicine, for example, for infection imaging (Petrik et al. 2021, Barrington et al. 2026). However, in the case of ^89^Zr, the resulting [^89^Zr]Zr-DFO 9 complex when conjugated to proteins has been shown to be unstable in vivo, leading to the release of osteophilic ^89^Zr^4+^ and consequently to unspecific accumulation of radioactivity in bones (Vugts et al. 2017, Feiner et al. 2021). As a result, an extended version of DFO 1, termed DFO* 2, was developed that fulfills the preferred octadentate coordination sphere of ^89^Zr. [^89^Zr]Zr-DFO* 10 exhibits an improved stability in vivo compared to [^89^Zr]Zr-DFO 9 (Vugts et al. 2017, Patra et al. 2014). DFO* 2 is currently considered as a potential new standard for ^89^Zr-complexation and investigated in several clinical trials for ^89^Zr-immunoPET applications (Oordt , Flavell , Monopar).

Meanwhile, Mindt and coworkers have reported an improved synthesis of DFO* 2 by adapting the protective group strategy, as well as a water-soluble version of the chelator, termed oxoDFO* 4, with oxygen atoms within the linker moieties connecting the four hydroxamate units (Briand et al. 2017, Guarrochena et al. 2024). Both DFO* 2 and oxoDFO* 4 have been shown to chelate efficiently and stably ^68^Ga in addition to ^89^Zr, thereby expanding the scope of their potential applications in nuclear medicine beyond ^89^Zr (Brandt et al. 2020). However, neither biological data on [^68^Ga]Ga-DFO* 6, [^68^Ga]Ga-oxoDFO* 8, or [^89^Zr]Zr-oxoDFO* 12 nor a comparison among them or with ^68^Ga/^89^Zr-labeled DFO have yet been published.

We herein report the efficient preparation of a novel, close analog of DFO*, named DFO*^sp^ 3, which is obtained by convenient solid phase (sp) synthesis in combination with the previously reported protective group strategy. The same approach was attempted for the preparation of oxoDFO* 4; however, it was not successful. DFO 1 (control), DFO* 2, novel DFO*^sp^ 3, and oxoDFO* 4, were radiolabeled with both [^68^Ga]GaCl_3_ and [^89^Zr]Zr-oxalate. The resulting radiometal complexes were evaluated in vitro and compared based on their physicochemical properties, such as log P, protein binding, and stability in different media, including blood serum. Finally, the in vivo stability and pharmacokinetic properties of [^68^Ga]Ga-DFO 5, [^68^Ga]Ga-DFO* 6, [^68^Ga]Ga-DFO*^sp^ 7, [^68^Ga]Ga-oxoDFO* 8, [^89^Zr]Zr-DFO 9, [^89^Zr]Zr-DFO* 10, [^89^Zr]Zr-DFO*^sp^ 11, and [^89^Zr]Zr-oxoDFO* 12 complexes were assessed in female balb/C mice, including ex vivo biodistribution experiments and microPET/CT imaging.

## Results

### Synthesis of monomers

**Scheme 1:**
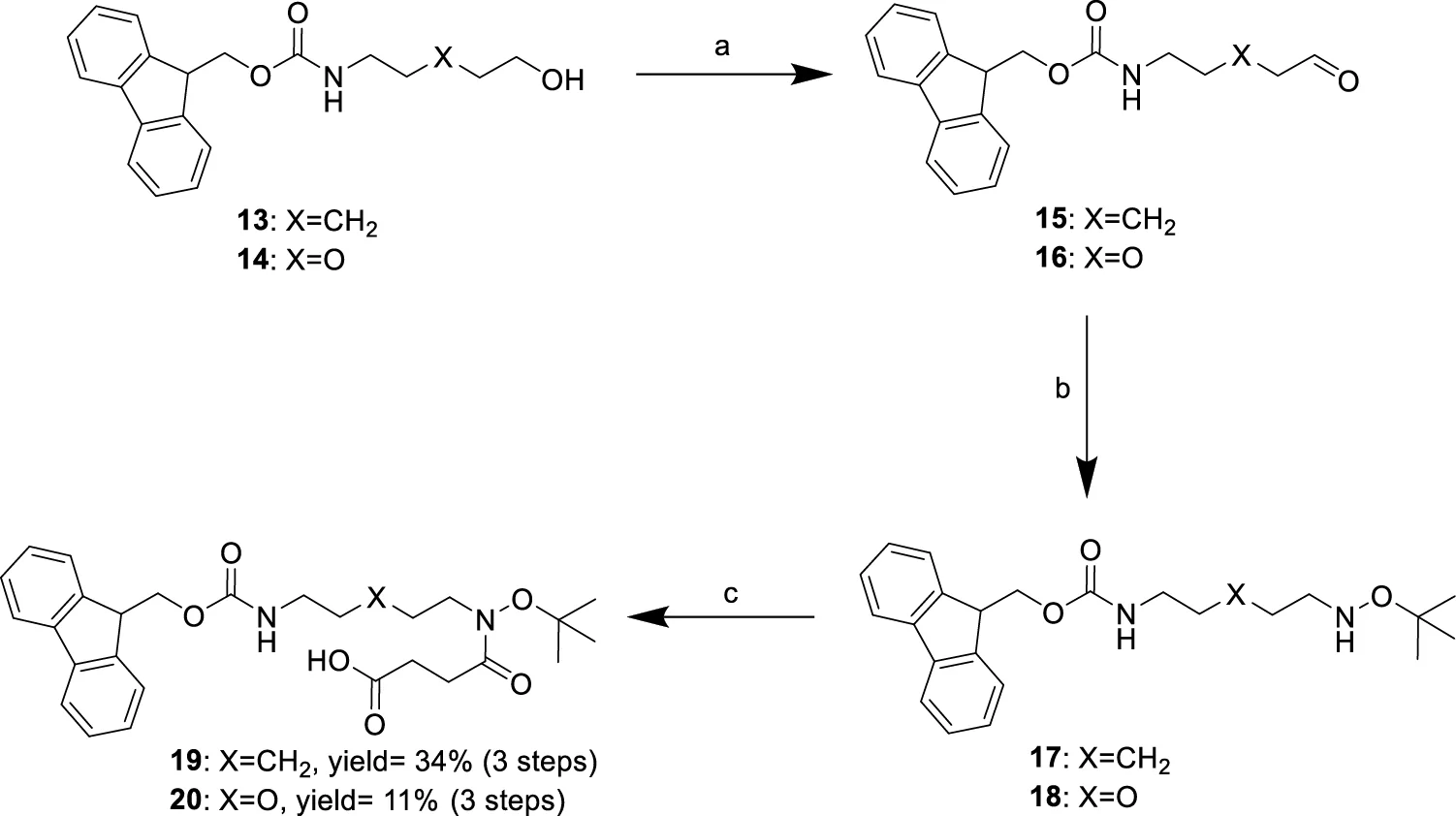
Synthesis of the monomers 19, 20. a: DMSO, Oxalyl Chloride, DIPEA, DCM, -45 °C; b: (1) NH_2_-OtBu in MeOH, RT (2) NaBH_3_CN, AcOH, MeOH, RT; c: succinic anhydride, DMAP, DMF, 50 °C. Yields: for 19 = 34% (3 steps), for 20 = 11% (3 steps)

**Scheme 2:**
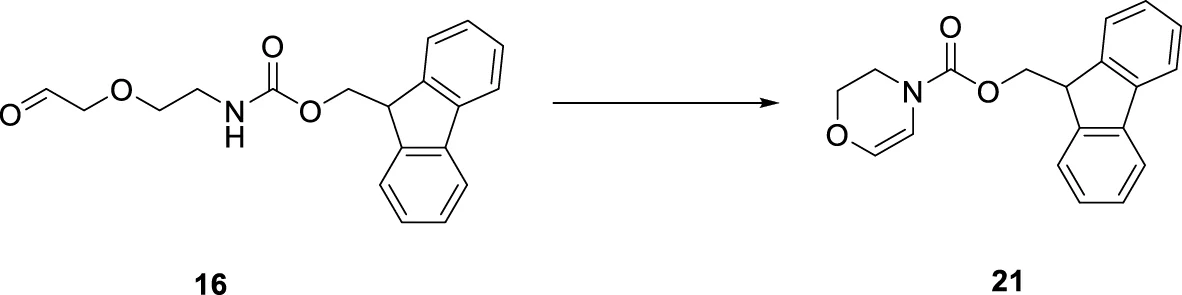
Intramolecular cyclization of compound 16, yielding undesired side product 21

The synthesis of the monomer for DFO*^sp^ 3 was carried out as described by Feiner et al. (Feiner et al. 2025). For oxoDFO* 4, the same method was used, using Fmoc-protected amino-alcohol 14 featuring the desired oxygen atom in the alkyl chain (Scheme 1). The first step involved a Swern oxidation of Fmoc-protected amino alcohols 13 and 14 to the corresponding aldehydes 15 and 16. In the case of alcohol 14, this step yielded an undesired cyclized side product (compound 21, Scheme 2). This byproduct could be isolated in a 40% yield and characterized. Therefore, the next step in the synthesis of the oxoDFO*-series was conducted with crude aldehyde 16 immediately after its isolation. Reductive amination of intermediates 15 and 16 was carried out with O-tert-butyl hydroxamate. The respective imine intermediates (Figure S1 and S23) were formed in situ overnight, after which they were reduced by the addition of NaBH_3_CN to afford products 17 and 18. The final step involved grafting of succinic anhydride onto the hydroxylamines 17 and 18 to form the desired protected monomers 19 and 20, with overall yields of 34% and 11%, respectively (3 steps). Final compounds were fully characterized by nuclear magnetic resonance (NMR) spectroscopy and high-resolution mass spectroscopy (HRMS).

### Synthesis of DFO* 2, DFO*^sp^ 3, and oxoDFO* 4

DFO* 2 was obtained from DFO 1 using a fragment condensation approach previously reported by Guarrochena et al. (Guarrochena et al. 2024).

**Scheme 3:**
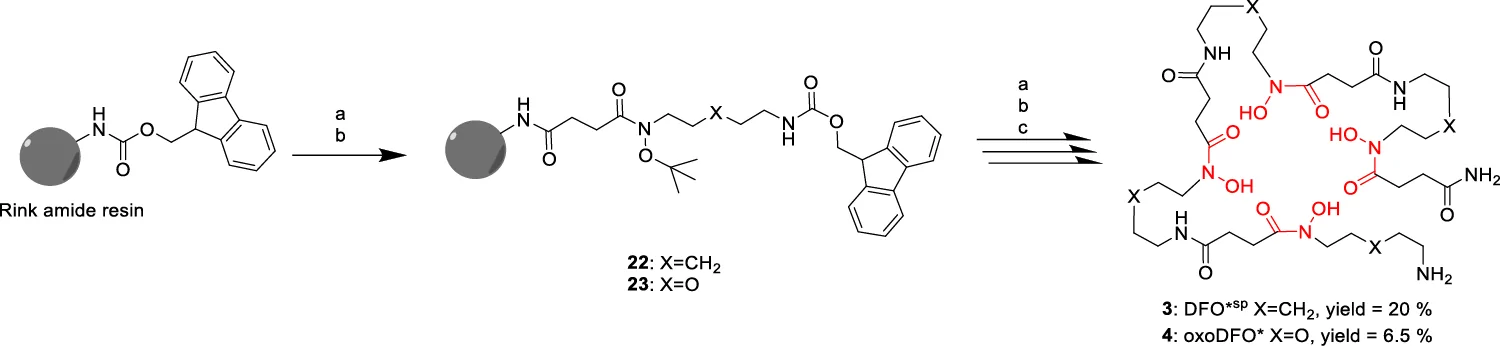
Synthesis of the chelators 3 and 4 on a rink amide resin (scale: 0.020 mmol; loading: 0.41 mol.g^-1^). a: DMF, 20% pyridine, 45 °C; b: monomer 19 or 20 (2.0 equiv.), HATU (2.0 equiv.), DIPEA (2.0 equiv.), DMF, 45 °C (by microwave), 1 h; c: TFA/H_2_O/TIPS (38:1:1), 1 h, 45 °C. Yields: for 3 = 20%, for 4 = 6.5%

Chelators 3 and 4 were synthesized on solid support using standard fluorenylmethoxycarbonyl (Fmoc)/ tertbutyl (tBu) chemistry on an automated microwave-assisted peptide synthesizer. All reagents and monomers 19 and 20 were dissolved in DMF, and the synthesis was performed following an adapted standard sp peptide synthesis protocol. The products were cleaved from the resin and simultaneously fully deprotected using a mixture of TFA/TIPS/H_2_O (98/1/1), precipitated in cold Et_2_O and purified by preparative-HPLC to give chelators 3 and 4 in yields of 20% and 6.5%, respectively, and a purity > 95%. The final products were characterized by NMR spectroscopy and HRMS (Scheme 3).

### (Radio)metal labeling of the chelators

DFO 1, DFO* 2, DFO*^sp^ 3, and oxoDFO* 4 were labeled with non-radioactive, natural (nat) isotopes of gallium and zirconium by adapting procedures from Jodal et al. (Jodal et al. 2014) and Patra et al. (Patra et al. 2014). The complexes were characterized by high-pressure liquid chromatography (HPLC) coupled to mass spectrometry (LC-MS), confirming a 1:1 metal-to-chelator ratio.

**Scheme 4:**
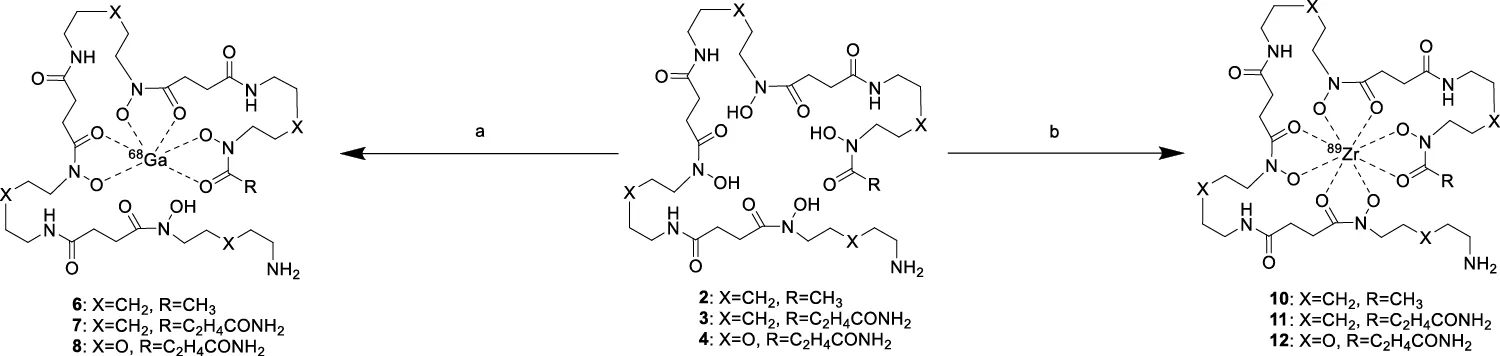
Radiolabeling of DFO analogs 2, 3, and 4 with [^68^Ga]Ga-chloride and [^89^Zr]Zr-oxalate. a: 300 µL [^68^Ga]GaCl_3_ in 0.1 M HCl aq., 130 µL sodium acetate (155 mg.ml^-1^), 10 µL DFO-analog (1 µg.µL^-1^), 95 °C, 15 min; b: 10 µL [89Zr]Zr in 0.5 M oxalic acid, 2.5 µL 2 M Na2CO3, 42.3 µL HEPES pH 7.4, 10 µL DFO- analog 1 mM, 37 °C, 1–2 h, shaking

DFO 1 and DFO-analogs 2, 3, and 4 were radiolabeled with [^68^Ga]GaCl_3_, applying published protocols ((Scheme 4; final chelator concentration: 10 µM, starting activity: 15–40 MBq, buffer: 1.15 M NaOAc pH 9.4) giving the respective complexes 5, 6, 7, and 8 (final pH 6–7) in radiochemical purity (RCP) and radiochemical yield (RCY) of > 95%, as confirmed by reversed-phase high-pressure liquid chromatography (RP-HPLC) and instant thin layer chromatography with silica gel stripes (iTLC-SG). DFO 1 and DFO-analogs 2, 3, and 4 were radiolabeled with [^89^Zr]Zr-oxalate using an established method (Brandt et al. 2020) (final chelator concentration: 5 µM, starting activity: 10–11 MBq, buffer: 0.5 M HEPES pH 7.4). Complexes 9, 10 and 11 formed quantitatively at RT after 1 h, while complex 12 required 2 h of incubation (Scheme 4). RCP and RCY of all complexes were > 95% as determined by RP-HPLC and iTLC-SG. The specific activity for all complexes was 2.5–3.6 MBq µg^− 1^ except for [^89^Zr]Zr–DFO 9 with 0.5–0.8 MBq µg^− 1^. The results of the radiolabeling reactions of the chelators with [^68^Ga]Cl_3_ and [^89^Zr]Zr-oxalate are summarized in Table 1.

**Table 1:** Radiochemical purity, yield and specific activities of [^68^Ga]Ga- and [^89^Zr]Zr-complexes 5–12. Results are given as mean values of *n* = 3 ± SD

Radiolabeled compound	RCP/RCY* [%]	Specific activity
[^68^Ga]Ga–DFO 5	99.8 ± 0.1	2.5-3.0 MBq µg^− 1^
[^68^Ga]Ga–DFO* 6	99.2 ± 1.0	2.5-3.0 MBq µg^− 1^
[^68^Ga]Ga–DFO*^sp^ 7	98.5 ± 1.4	2.5-3.0 MBq µg^− 1^
[^68^Ga]Ga–oxoDFO* 8	97.4 ± 1.8	2.5-3.0 MBq µg^− 1^
[^89^Zr]Zr–DFO 9	99.6 ± 0.4	0.5–0.8 MBq µg^− 1^
[^89^Zr]Zr–DFO* 10	97.9 ± 2.0	3.6 MBq µg^− 1^
[^89^Zr]Zr–DFO*^sp^ 11	99.1 ± 0.3	2.6 MBq µg^− 1^
[^89^Zr]Zr–oxoDFO* 12	99.0 ± 0.4	2.6 MBq µg^− 1^

Note: * The complexes were not purified and as a result, the RCY equals the RCP

### In vitro assays

In vitro assays were compared with reference complexes [^68^Ga]Ga-DFO 5 and [^89^Zr]Zr-DFO 9 (Table 2, and Table S1-2). The ^68^Ga-labeled complexes exhibited a hydrophilic character similar to those of the ^89^Zr-analogs - with a logP ranging from − 3,3 to -2,4. For an assessment of the stability of the radiometal complexes, they were incubated in PBS or human serum for 2 h (^68^Ga) or 48 h (^89^Zr). The stability of most complexes was above 95% in both media. In the case of [^68^Ga]Ga-DFO*^sp^ 7, the stability was slightly lower in PBS and in human serum in comparison to the other compounds. Also, the stability of [^89^Zr]Zr-DFO*^sp^ 11 in human serum was found decreased, with 90% intact complex left after 48 h. Protein binding in human serum was generally low for all complexes with the exception of [^68^Ga]Ga-DFO 5 and [^68^Ga]Ga-DFO* 6, for which unexpectedly high protein binding was observed. Overall, the protein binding was in general lower in mouse serum than in human serum but in the case of [^89^Zr]Zr-DFO 9 the opposite was true.

**Table 2:** Physico-chemical properties of complexes 5, 6, 7, 8, 10, 11, and 12. Results are given as mean values of *n* = 3 ± SD

Radiolabeled compound	Log P (*n* = 6)	Protein binding in human serum [%]	Protein binding in mouse serum [%]	Stability in human serum [%]	Stability in PBS [%]
[^68^Ga]Ga-DFO 5	-3.3 ± 0.2	26.7 ± 3.9^1^	1.7 ± 0.3^1^	98.4 ± 0.5^1^	98.0 ± 1.2^1^
[^68^Ga]Ga-DFO* 6	-2.4 ± 0.3	30.2 ± 2.5^1^	4.3 ± 0.1^1^	98.6 ± 1.1^1^	98.5 ± 1.0^1^
[^68^Ga]Ga-DFO*^sp^ 7	-3.2 ± 0.1	4.4 ± 1.2^1^	4.3 ± 0.9^1^	88.2 ± 5.6^1^	94.8 ± 1.8^1^
[^68^Ga]Ga-oxoDFO* 8	-3.4 ± 0.3	9.6 ± 1.1^1^	6.5 ± 1.1^1^	96.4 ± 0.7^1^	96.9 ± 2.3^1^
[^89^Zr]Zr-DFO 9	-2.7 ± 0.1	3.4 ± 1.1^2^	13.0 ± 1.6^2^	93.4 ± 7.4^2^	99.9 ± 0.1^2^
[^89^Zr]Zr-DFO* 10	-2.6 ± 0.1	4.1 ± 1.2^2^	5.8 ± 1.1^2^	98.1 ± 1.8^2^	97.8 ± 0.9^2^
[^89^Zr]Zr-DFO*^sp^ 11	-2.4 ± 0.3	7.1 ± 5.0^2^	5.5 ± 1.5^2^	90.4 ± 8.3^2^	97.6 ± 0.3^2^
[^89^Zr]Zr-oxoDFO* 12	-2.9 ± 0.2	6.3 ± 1.7^2^	2.3 ± 0.5^2^	96.4 ± 2.9^2^	99.8 ± 0.1^2^

Note: ^1^: after 2 h; ^2^: after 48 h. Values for [^68^Ga]Ga-DFO **5** correspond to previously published data (Petrik et al. 2021) and are included for comparison

### In vivo serum stability in mice

Stability of radiometal complexes in vivo was tested in balb/C mice (*n* = 3 per time point). The tested complexes were shown to be stable in vivo. Stability in blood was measured 5 min after administration of studied radiometal complexes and exceeded 90% for all [^68^Ga]Ga- and [^89^Zr]Zr-labeled DFO-analogs (Table 3) but [^68^Ga]Ga-DFO*^sp^ **7**. Stability was measured by RP-HPLC based on the integration of the peaks of radiolabeled chelators (retention time (r_t_) = 11–15 min) and the peaks of free ^68^Ga (r_t_= 3.8–4.9 min) and ^89^Zr (r_t_= 2.5–3.2 min) min; see Supporting information, Figure S52-S67).

**Table 3:** In vivo stability of [^68^Ga]Ga- and [^89^Zr]Zr-DFO-analogs 5–12. Results are given as mean values of *n* = 3 ± SD

Radiolabeled compound	stability in blood after 5 min [%]
[^68^Ga]Ga-DFO 5	96.8 ± 2.9
[^68^Ga]Ga-DFO* 6	64.4 ± 3.5
[^68^Ga]Ga-DFO*^sp^ 7	80.1 ± 0.6
[^68^Ga]Ga-oxoDFO* 8	94.5 ± 0.5
[^89^Zr]Zr-DFO 9	98.6 ± 0.6
[^89^Zr]Zr-DFO* 10	98.6 ± 1.1
[^89^Zr]Zr-DFO*^sp^ 11	97.5 ± 1.4
[^89^Zr]Zr-oxoDFO* 12	97.3 ± 0.1

### Biodistribution in balb/C mice

**Fig. 2:**
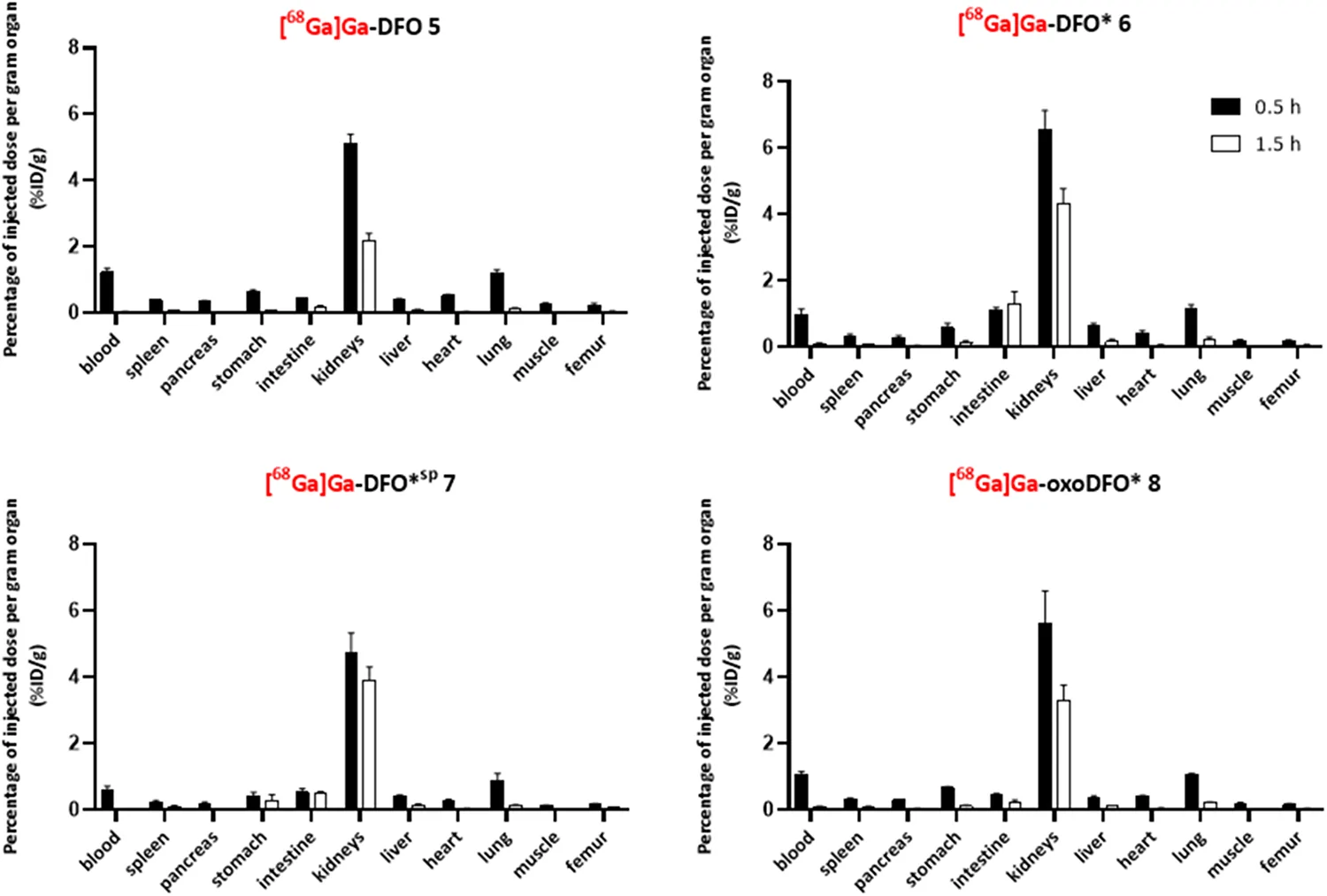
Ex vivo biodistribution of [^68^Ga]Ga-DFO 5 (Petrik et al. 2021) in comparison with [^68^Ga] Ga-DFO-analogs 6–8 in balb/C mice after 0.5 and 1.5 h p. i. Results are given as mean values of *n* = 3 ± SD

**Fig. 3:**
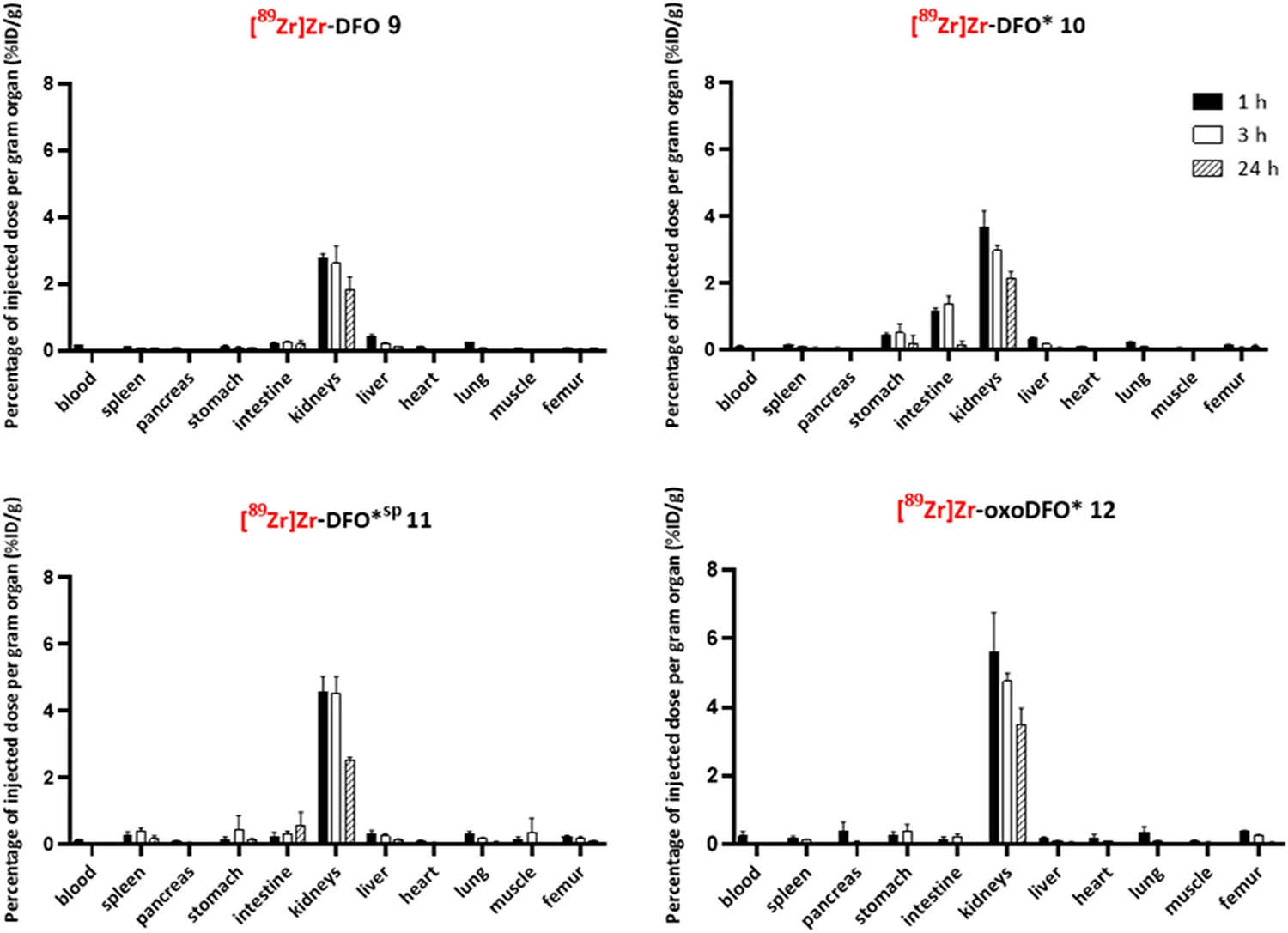
Ex vivo biodistribution of [^89^Zr] Zr-DFO-analogs 9–12 in balb/C mice after 1, 3, and 24 h p. i. Results are given as mean values of *n* = 3 ± SD

The ex vivo biodistribution data of [^68^Ga]Ga- (Fig. 2) and [^89^Zr]Zr-complexes (Fig. 3) displayed rapid renal elimination of radioactivity with minimal retention in blood and other organs and tissues already at early time points (0,5 h and 1 h for [^68^Ga]Ga- and [^89^Zr]Zr-complexes, respectively; Tables S3-S10). The highest accumulation of radioactivity at all time points was found in the kidneys, which decreased over time. Particularly at early time points, low concentrations of radioactivity were also detected in the intestines, stomach and well-perfused organs such as lung, liver, and heart. At the latest timepoint, a very low background signal was generally observed, with the exception of the kidneys for all tested radiometal complexes. Only the radiolabeled DFO*-complexes 6 and 10 showed retention of radioactivity in the intestines over time ([^68^Ga]Ga-DFO* 6: 1.12 ± 0.08%ID/g 0.5 h and 1.29 ± 0.38%ID/g 1.5 h; [^89^Zr]Zr-DFO* 10 (1.15 ± 0.1%ID/g 1 h; 1.39 ± 0.23%ID/g 3 h and 0.14 ± 0.12%ID/g 24 h). No, or negligible, uptake of radioactivity in the bones was observed for all radiolabeled chelators.

### PET/CT imaging

**Fig. 4:**
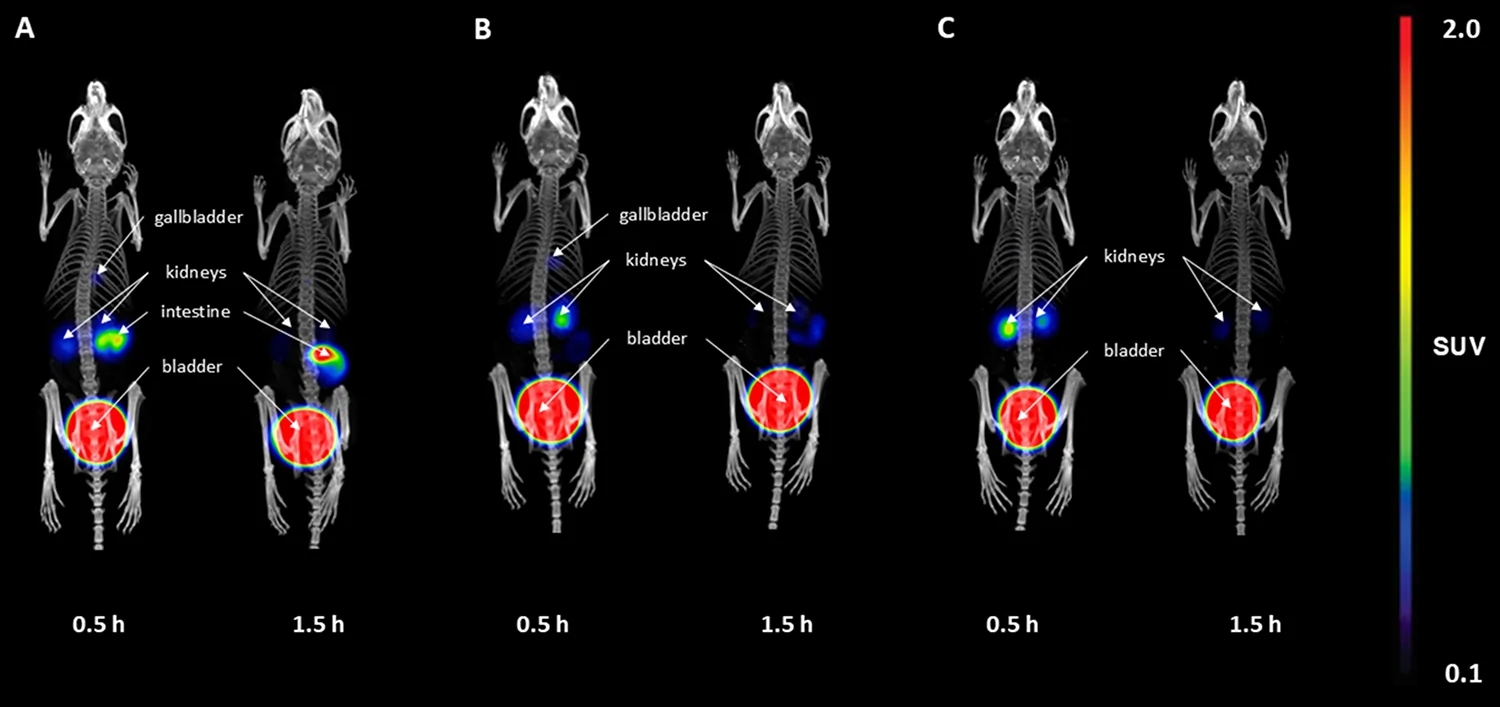
Example of maximum intensity projection (MIP) PET/CT images of in vivo [^68^Ga]Ga-DFO-analogs ([^68^Ga]Ga-DFO* 6 (**A**), [^68^Ga]Ga-DFO*^sp^ 7 (**B**), [^68^Ga]Ga-oxoDFO* 8 (**C**)) in balb/C mice 0.5 and 1.5 h p. i

**Fig. 5:**
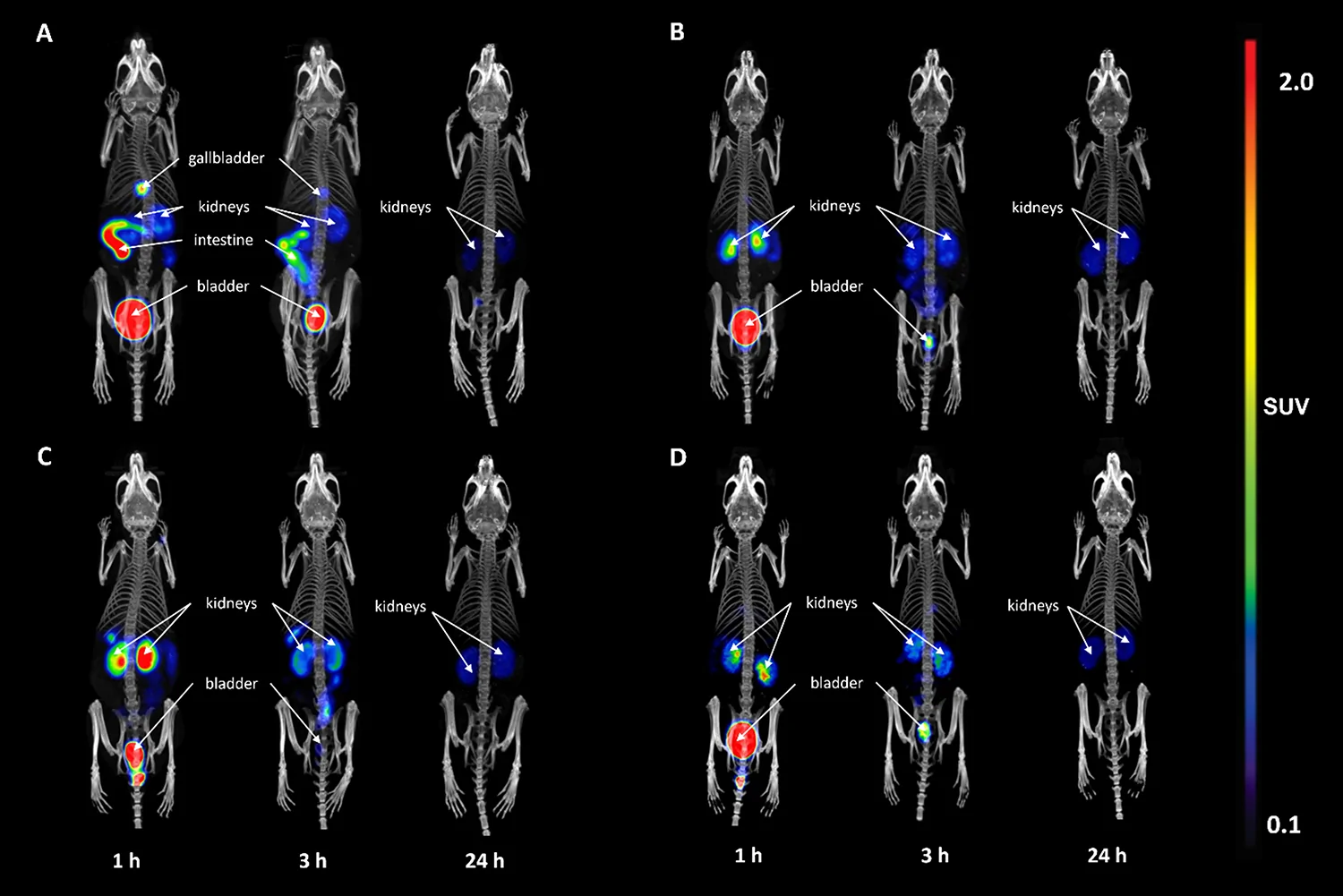
Example of maximum intensity projection (MIP) PET/CT images of in vivo [^89^Zr]Zr-DFO-analogs ([^89^Zr]Zr-DFO* 10 (**A**), [^89^Zr]Zr-DFO*^sp^ 11 (**B**), [^89^Zr]Zr-oxoDFO* 12 (**C**)) and [^89^Zr]Zr-DFO 9 (**D**) in balb/C mice 1, 3, and 24 h p. i

PET/CT imaging of [^68^Ga]Ga- (Fig. 4) and [^89^Zr]Zr-complexes (Fig. 5) in balb/C mice confirmed the results from ex vivo biodistribution studies. All radiometal complexes were rapidly cleared from the bloodstream and excreted primarily via the kidneys into the bladder. Some accumulation of radioactivity was observed in the intestines and additionally, in the gall bladder. With the exception of the organs involved in the renal excretion of the radiolabeled chelators, the background levels resulting from the uptake of radioactivity in other organs and tissue were found favorably low, particularly at later time points.

## Discussion

This work aims to present a new synthesis pathway for hydroxamate-based chelators and to compare their chelation properties for ^89^Zr and ^68^Ga as well as the in vitro and in vivo characteristics of the respective complexes. Different groups have already developed methods for synthesizing hydroxamate-based chelators for ^89^Zr, Seibold et al. designed a cyclized version of DFO* but the monomer synthesis was giving a very poor yield (Seibold et al. 2017). We have previously reported the use of carba-monomer 19 for the sp synthesis of DFO*^12^, an extended, dodecadentate analog of DFO* 2 suitable for the complexation of actinium-225 (Feiner et al. 2025). With the goal to simplify also the synthesis of DFO* 2, we prepared DFO*^sp^ 3 from the same building block by convenient automated sp synthesis. DFO*^sp^ 3 is a new acyclic DFO* analog which differs from DFO* 2 regarding its terminus and previously reported oxoDFO* 4 due to the incorporation of heteroatoms, respectively, but not in the arrangement of the four hydroxamate groups important for ^89^Zr-complexation (Fig. 1). The yield of DFO*^sp^ 3 by sp synthesis was in the same range as the one achieved for DFO* 2 using the previously reported fragment condensation approach (approx. 25–30% from carba-monomer 19, after HPLC purification). However, sp synthesis circumvents issues with the solubility of starting materials, intermediates, and products and offers a straightforward possibility for scaling-up the preparation of DFO* and analogs.

Carba-monomer 19, with a tBu-protected hydroxamate, was designed not only to accommodate Fmoc-sp synthesis and simultaneous global deprotection and cleavage of products from the resin in a single step, but also to avoid a dehydroxylation side reaction observed during final deprotection by hydrogenation in the case of the reported benzyl (Bn) protective group. However, implementation of the tBu-protective group strategy to oxo-monomer 20 and subsequently oxoDFO* 4 was not advantageous. During the synthesis of oxo-monomer 20, we observed that the aldehyde intermediate 16 in the oxo-series underwent a spontaneous cyclization, forming the side product 21 (isolated yield 40%), likely as a result of the electron-withdrawing effect (inductive -I) of the additional oxygen atom in the alkyl spacer (Scheme 2, Scheme S1).

While the side reaction forming compound 21 could be partly suppressed by using crude aldehyde 16 immediately without isolation, the overall yield of oxo-monomer 20 remained moderate (11% versus 34% for carba-monomer 19). We conclude that the originally described Bn protective group is better suited for the synthesis of oxoDFO* 4, despite the risk of partial dehydroxylation during its final hydrogenolytic removal.

With DFO 1 (control), DFO* 2, DFO*^sp^ 3, and oxoDFO* 4 in hands, we next compared their radiolabeling with ^68^Ga and ^89^Zr using established protocols. The labeling of DFO 1, DFO* 2, and oxoDFO* 4 with ^68^Ga and ^89^Zr was previously performed in our group and others (Chomet et al. 2021); the radiolabeling of all chelators, including the new DFO*^sp^ 3, at RT within a short time resulted in high RCY, RCP and good specific activities for all complexes. In the case of oxoDFO* 4 with ^89^Zr, longer incubation times were needed to achieve quantitative complexation of the radiometals, which is consistent with previous observations (Brandt et al. 2020).

The partition coefficient (logP) of ^68^Ga/^89^Zr-labeled chelators was determined by the shake flask method. All complexes were found favorably hydrophilic with the oxoDFO* complexes exhibiting, as expected, the lowest logP values in the respective radiometal series ([^68^Ga]Ga-oxoDFO* 8: -3,4 and [^89^Zr]Zr-oxoDFO* 12: -2,9). The slightly higher lipophilicity of the all-carbon analogs might reflect in part some of the differences observed in vivo as described below.

The in vitro stability of radiolabeled DFO 1 and DFO-analogs 2–4 was evaluated in human serum, as well as in PBS. Also, their in vitro protein binding properties were investigated in mouse and human serum. The results were compared with that of radiolabeled reference DFO 1 (Petrik et al. 2021). All ^68^Ga- and ^89^Zr-complexes exhibited high stability in the media tested with > 90% intact compounds after 2–48 h respectively. At first instance, the stability of [^68^Ga]Ga-DFO*^sp^ 7 and [^89^Zr]Zr-DFO*^sp^ 11 appeared to be lower than the other complexes tested, however, the differences were not significant with respect to the standard deviation of the experimental results. Protein binding was generally low for all ^68^Ga/^89^Zr-labeled chelators (2,3–13,0%) but unexpectedly high in human serum for [^68^Ga]Ga-DFO 5 (26,7%) and of [^68^Ga]Ga-DFO* 6 (30,2%). This interspecies difference may explain the long half-life in the human bloodstream observed during first clinical trials of [^68^Ga]Ga-DFO 5 for investigating its utility in infection PET imaging (Medizinische Universität 2020). The low binding of these compounds to murine serum proteins was consistent with their rapid blood clearance observed in vivo. However, HPLC analysis of blood samples collected 5 min after intravenous administration revealed differences in the proportion of intact radiolabeled siderophore among the individual ^68^Ga complexes (concrete in the case of complexes 6 and 7), indicating that their favorable in vitro stability did not necessarily translate into comparable in vivo stability. Overall, the radiolabeled DFO-analogs showed promising in vitro properties similar or superior to that of the reference compounds [^68^Ga]Ga-DFO 5 and [^89^Zr]Zr-DFO 9.

Evaluation of the pharmacokinetics of ^68^Ga/^89^Zr-labeled chelators in balb/C mice by biodistribution and PET/CT-imaging revealed good serum stability, fast clearance from blood, tissue and organs as well as primarily renal excretion. These results can be attributed to the hydrophilicity of the compounds as well as low binding to murine serum proteins. As a result, highest uptake of radioactivity was observed in the kidneys and bladder for all compounds tested. Some uptake of radioactivity was also found in the stomach, gall bladder, and intestines, which indicates partial hepatobiliary excretion. This effect was more pronounced for ^68^Ga/^89^Zr-labeled DFO all-carbon analogs with less hydrophilic character than radiometal complexes of oxoDFO* 4. This finding indicates potential advantages of using oxoDFO* 4 instead of DFO* 2 for some applications in nuclear medicine. Noteworthy, no or only negligible uptake of osteophilic ^89^Zr^4+^ in the bones was observed even with [^89^Zr]Zr-DFO 9, a complex which is known to release the radiometal over time when conjugated to antibodies exhibiting a blood circulation time longer than the time period tested in our experiments with fast clearing, low molecular weight, and hydrophilic chelates (Fig. 5). These observations are consistent with the experiments using [^89^Zr]Zr-DFO 9 and [^68^Ga]Ga-DFO 5 reported by Chomet et al. in 2021 and Petrik et al. (Chomet et al. 2021, Petrik et al. 2021).

In summary, the in vivo properties of ^68^Ga/^89^Zr-complexes of the new DFO*^sp^ 7 and 11 and previously not preclinically studied oxoDFO* 4 make them equally promising alternative candidates for the future development of PET radiotracers as the already studied analogous DFO 1 and DFO* 2.

## Conclusion

We report herein the sp synthesis of DFO*^sp^ 3, a novel close analog of DFO* 2. Sp synthesis enables a convenient and efficient synthesis of DFO*^sp^ 3 and potentially also other structurally diverse DFO*-analogs. However, the sp strategy will likely need to be tailored towards the desired monomer and/or product in terms of applied protective groups and reaction conditions, as experienced in the case of oxoDFO* 4. All chelators, DFO 1 (control), DFO* 2, DFO*^sp^ 3, and oxoDFO* 4 formed quantitatively the corresponding ^68^Ga- and ^89^Zr-complexes at RT within 1–2 h in high RCY, RCP and specific activities. The ^68^Ga-complexes 5–8 and ^89^Zr-complexes 9–12 were shown to have favorable hydrophilic character, low protein binding properties, and high stability in PBS and blood serum (in vitro and ex vivo). In balb/C mice, the ^68^Ga- and ^89^Zr-complexes had fast clearance predominantly via renal excretion and, in general, low uptake in organs and tissues with exception of the kidney and bladder. The in vivo and in vitro evaluation of ^68^Ga- and ^89^Zr-complexes of DFO*^sp^ 3 in direct comparison to DFO 1 (control), DFO* 2, and oxoDFO* 4 demonstrated favorable properties of all compounds which makes this class of oligo-hydroxamates promising chelator candidates for the development of ^68^Ga/^89^Zr-based radiopharmaceuticals for immunoPET, infection imaging, or other applications in nuclear medicine.

## Materials and methods

Reagents and biological media (blood and serum) were purchased from Sigma-Aldrich (Buchs, Switzerland), Merck (Darmstadt, Germany), abcr (Karlsruhe, Germany), Acros Organics (Geel, Belgium), Fluorochem (Hadfield, United Kingdom), Novabiochem (Darmstadt, Germany), TCI (Zwijndrecht, Belgium), and Fluka (Buchs, Switzerland) and were used without further purification. DFO and DFO* were purchased from ABX (Radeberg, Germany). DFO* (Feo et al. 2022) was also obtained using a method previously reported (Guarrochena et al. 2024). The monomer for DFO*^sp^ 19 was synthesized according to a previously reported method (Feiner et al. 2025).

The TLC-plates used for the monitoring of the reactions were purchased from Macherey-Nagel (DC-Fertigfolien ALUGRAM^®^ Xtra SIL G/UV_254_). The chelators were synthesized using the sp Fmoc chemistry on the microwave peptide synthesizer Biotage^®^ Initiator+ Alstra™. RP-HPLC-MS analyses were conducted at l = 220 nm on an Agilent 1260 Infinity II system equipped with a Flexible pump, a 1260 VWD UV-Vis detector, and the LC-MSD system with an Acquity UPLC^®^ BEH C18 column (300 Å, 1.7 μm, 2.1 mm x 100 mm). Mobile phase A is H_2_O (0.1% formic acid (FA)) and mobile phase B is acetonitrile (can with0.1% FA). Flow rate was 0.6 mL/min. For preparative RP-HPLC, a Waters XBridge C18 column was used on an Agilent 1200 Series system. The methods used are described in the respective protocol. HRESI-MS spectra for the monomer (m/z 50-1900) were obtained on a maXis UHR ESI-Qq-TOF mass spectrometer (Bruker Daltonics, Bremen, Germany) in the positive-ion mode by direct infusion. HRESI-MS spectra for the chelator (m/z 20-40000) were obtained on an ESI/MALDI-Qq-TOF with dual-TIMS analyzer mass spectrometer (Bruker Daltonics, Bremen, Germany) in the positive-ion mode by direct infusion. The sum formulas of the detected ions were determined using Bruker Compass Data Analysis 4.1 based on the mass accuracy (Δm/z ≤ 5 ppm) and isotopic pattern matching (SmartFormula algorithm). NMR spectroscopy measurements were recorded on a Bruker FT-NMR Avance III 500 and 600 MHz spectrometer at 298 K in deuterated methanol (Methanol-d4), deuterated chloroform (Chloroform-d), or deuterated dimethyl sulfoxide (DMSO-d6). The respective residual solvent peak was used as an internal reference for the chemical shifts (ppm). Spin multiplicities are abbreviated as follows: singlet, d=doublet, t=triplet, q=quartet, m=multiplet, bs=broad signal. The values of the coupling constants (*J*) are given in Hertz (Hz). For centrifugation, a ROTINA 380/380R from Hettich was used. Microwave reactions were performed with a Biotage Initiator+ system. The device has a range of power up to 400 W and takes about 1 min to heat to the desired temperature.

[^68^Ga]GaCl_3_ was obtained from a ^68^Ge/^68^Ga-generator (Eckert & Ziegler Eurotope GmbH, Berlin, Germany) using a fractionated elution method with 0.1 M HCl (Petrik et al. 2011).

The radiochemical purity of the ^68^Ga-radiotracers was evaluated using radio-RP-HPLC, consisting of a Dionex UltiMate 3000 (Thermo Scientific, Waltham, Massachusetts, USA) with a GABI Star radiation detector (Raytest, Straubenhardt, Germany); column: Nucleosil 120–5 C18 250 × 4 mm (WATREX, Prague, Czech Republic); flow rate: 1 mL/min; oven temperature: 25 °C; UV detection wavelengths 225 nm and 250 nm; mobile phase gradient: acetonitrile (ACN) with 0.1% trifluoroacetic acid (TFA) in water; 0–3 min, 0% ACN; 3–6 min, 0–50% ACN; 6–10 min, 50–80% ACN; 10–13 min, 80% ACN; 13–15 min, 0% ACN. Silica-gel-impregnated glass microfibre chromatographic papers (Varian, Lake Forest, CA, USA) were used for radio-iTLC-SG analysis of ^68^Ga-labelings. Chromatographic paper strips containing a sample of the [^68^Ga]Ga-DFO*/DFO*^sp^/oxoDFO* complex were developed in a chamber saturated with diethylenetriamine pentaacetate DTPA (0.05 M) or sodium citrate dihydrate (0.1 M, pH 5). After development, the strips were scanned using a radiometric phosphor imager (Cyclone Plus Storage Phosphor System, PerkinElmer, Waltham, MA, USA), and the chromatograms for each strip were evaluated.

[^89^Zr]Zr-oxalate was purchased from Revvity (Zirconiumcity, Oxalateland) and used directly without any purification. Instant Thin Layer Chromatography paper impregnated with a silica gel (iTLC-SG; Agilent, Santa Clara, United States) was used for the monitoring of the ^89^Zr-labelings. iTLC-SG paper strips containing a sample of the [^89^Zr]Zr-DFO*/DFO*^sp^/oxoDFO* complex were developed in a chamber saturated with aqueous citrate buffer (0.1 M, pH 4). After development, the strips were scanned using a miniGITA Dual from Elysia-Raytest. In cases where no clear quantification could be performed, the iTLC-SG strips were cut at approximately 3.5 cm from the elution front, and both halves were measured using a 2480 Wizard2 1-Detector γ-counter with an energy window of 500–1000 keV (crystal: NaI (Tl), 80 mm in height, 75 mm in diameter; Perkin Elmer).

Protein binding of ^68^Ga- and ^89^Zr-complexes was assessed via size exclusion chromatography using MicroSpin G-50 columns (Mw cut-off 30 kDa) or Sephadex G-50, GE Healthcare, Buckinghamshire, UK.

Animal imaging was performed with a Mediso NanoScan PET/CT small animal imaging system (Mediso Medical Imaging Systems, Budapest, Hungary).

All spectra used for the identification and characterization of the compounds are available in the supporting information document (Figure S2-S22 and S24-S49).

Statistical analysis of radiolabeling and stability of the complexes in vitro and ex vivo was calculated using the AVERAGE and STDEV.P functions of Microsoft^®^ Excel^®^ for Microsoft 365 MSO (Version 2607 Build 16.0.20228.20110) 64-bit.

### Syntheses of the oxo-monomer 20

All spectra used for the characterization of compounds, as well as HPLC chromatograms can be found in the Supporting Information (SI).

Oxo-monomer 20 was synthesized by adapting a method previously reported (Feiner et al. 2025). The full procedure is described below:

Amino aldehyde 16. To a 1 M solution of DMSO (1.74 mL, 34 mmol, 2.2 equiv.) in anhydrous dichloromethane (34 mL), a 2 M solution of oxalyl chloride (2.1 mL, 18.6 mmol, 1.2 equiv.) in anhydrous dichloromethane (9 mL) was added dropwise over 5 min at -45 °C (dry ice/acetonitrile). The reaction was stirred for 10 min, and a 1.3 M solution of the amino alcohol 14 (5 g, 15.5 mmol, 1.0 equiv.) in anhydrous dichloromethane (12 mL) was added dropwise over 10 min at -45 °C. The reaction was stirred for 30 min at -45 °C, followed by the addition of N, N-diisopropylethylamine (8.1 mL, 46.5 mmol, 3 equiv.), after which the temperature was allowed to increase to -20 °C and the mixture was stirred for 90 min. The reaction was monitored by thin-layer chromatography (TLC, 40/60, Ethyl acetate/Hexane; Rf = 0.5). Upon completion of the reaction indicated by TLC, the solvent was removed under reduced pressure, the crude product was dissolved in dichloromethane (30 mL) and washed three times each with 1 M KHSO_4_ (20 mL) and brine (20 mL). The organic phase was dried over Na_2_SO_4_, filtered, and the solvent was removed under reduced pressure to yield amino aldehyde 16 as a pale yellow oil. Due to its low stability, the crude amino aldehyde 16 was used for the next step without further purification.

The side product compound 21 was isolated from the reaction of 500 mg (1.53 mmol) of amino alcohol 14 as described for aldehyde 16 and isolated by normal-phase manual flash chromatography. Solvent A: hexane, Solvent B: Ethyl Acetate with a 100 g column of SiO_2_ (Particle size: 0.04–0.063 μm) with an isocratic gradient of 40% of solvent A. The fractions containing the compound were combined, concentrated under reduced pressure, and dried in high vacuum to give the compound 21 as a white solid (m = 205.4 mg, yield 40%).

Analysis for compound 21 (Note: To avoid the doubling of the signal of the carbon-carbon double bond due to the presence of isomers, this compound was measured at 100 °C):

^1^H NMR (500 MHz, DMSO) δ 7.87 (d, *J* = 7.5 Hz, 2 H), 7.63 (d, *J* = 7.5 Hz, 2 H), 7.42 (t, *J* = 7.4 Hz, 2 H), 7.34 (t, *J* = 7.4 Hz, 2 H), 6.06 (s, 1H), 6.00 (d, *J* = 4.8 Hz, 1H), 4.49 (d, *J* = 6.4 Hz, 2 H), 4.32 (t, *J* = 6.3 Hz, 1H), 4.00–3.97 (m, 2 H), 3.58–3.55 (m, 2 H) ppm.

^13^C NMR (125.75 MHz, DMSO) δ 150.80, 143.26, 140.37, 129.06, 127.09, 126.53, 124.29, 119.45, 104.93, 66.54, 63.65, 46.43, 41.03 ppm.

ESI-HR-MS m/z found (calculated for C_19_H_17_NO_3_Na): [M + Na^+^]^+^ = 330.1092 (330.1101).

Hydroxylamine 18. A 0.1 M solution of the crude amino aldehyde 16 (5.0 g, 15.5 mmol, 1.0 equiv.) was prepared in methanol (150 mL). *O*-*t*Bu-hydroxylamine-hydrochloride (5.8 g, 46.5 mmol, 3.0 equiv.) was added, and the mixture was stirred at RT overnight. The completion of the reaction was verified by TLC (40/60, Ethyl acetate/Hexane; Rf = 0.4), and the solvent was evaporated under reduced pressure. The crude mixture was dissolved in methanol (15 mL), and the reduction was conducted by adding NaBH_3_CN (4.1 g, 65.1 mmol, 4.2 equiv.) and acetic acid (4.4 mL, 77.5 mmol, 5.2 equiv.) at RT. The reaction was stirred for 3.5 h at RT, and the conversion to the desired hydroxylamine was confirmed by TLC. After evaporation of methanol, the crude product was dissolved in dichloromethane (60 mL) and washed three times with sat. NaHCO_3_ (40 mL) and brine (40 mL). The organic phase was dried over Na_2_SO_4_, filtered, and the solvent was removed under reduced pressure to yield hydroxylamine. Hydroxylamine 18 was obtained as a yellow oil and used directly for the next step without further purification.

^1^H NMR (500 MHz, Chloroform-d) δ 7.79–7.74 (m, 2 H), 7.63–7.58 (m, 2 H), 7.43–7.37 (m, 2 H), 7.35–7.29 (m, 2 H), 4.45–4.37 (d, *J* = 6.9 Hz, 2 H), 4.27–4.18 (t, *J* = 6.8 Hz, 1H), 3.61–3.56 (t, *J* = 5.0 Hz, 2 H), 3.56–3.51 (t, *J* = 4.9 Hz, 2 H), 3.43–3.36 (m, 2 H), 3.05–2.97 (t, *J* = 5.1 Hz, 2 H), 1.19 (s, 9 H) ppm.

ESI-MS m/z found (calculated for C_23_H_30_N_2_O_4_): [M + H^+^]^+^ = 399.22 (399.23).

Monomer 20. The crude hydroxylamine 18 (6.0 g, 15.5 mmol, 1.0 equiv.) was dissolved in dimethylformamide (12 mL). Succinic anhydride (7.8 g, 77.5 mmol, 5.0 equiv.) and 4-(dimethylamino) pyridine (950 mg, 7.75 mmol, 0.5 equiv.) were added at RT. The reaction was stirred for 90 min at 50 °C. The conversion to the desired monomer was monitored by HPLC-MS (45–85% ACN/H_2_O over 6 min with 0.1% FA). DMF was removed under high vacuum at 40 °C, and the crude product was dissolved in methanol (200 mg/mL) for its subsequent purification *via* preparative-HPLC: flow rate: 17 mL/min, mobile phase A = H_2_O + 0.1% FA, mobile phase B = MeOH + 0.1% FA, gradient: 0–3.5 min 65% B, 23.5 min 85% B, 24.5 min 95% B, 29.5 min 95% B, 30.0 min 65% B. The fractions containing the product, with a purity above 95%, were pooled and lyophilized to obtain Monomer 20 as a white powder, with a yield over three steps of 11%.

^1^H NMR (600 MHz, Methanol-d4) δ 7.82–7.77 (m, 2 H), 7.68–7.62 (m, 2 H), 7.41–7.36 (m, 2 H), 7.34–7.28 (m, 2 H), 4.41–4.32 (d, *J* = 6.9 Hz, 2 H), 4.25–4.16 (t, *J* = 6.8 Hz, 1H), 3.70–3.59 (m, 2 H), 3.51–3.41 (m, 2 H), 3.27–3.22 (m, 2 H), 2.81–2.71 (m, 2 H), 2.61–2.50 (m, 2 H), 1.31 (s, 9 H) ppm. One CH_2_ signal overlaps with the solvent peak (Methanol-d4, δ 3.31 ppm) as evidenced in the HSQC (Figure S30).

^13^C NMR (151 MHz, Methanol-d4) δ 176.50, 158.86, 145.36, 142.61, 128.77, 128.15, 126.61, 121.00, 120.92, 70.71, 67.69, 67.67, 49.57, 48.47, 41.74, 29.77, 27.85, 23.77, 23.04 ppm.

ESI-HR-MS m/z found (calculated for C_27_H_34_N_2_O_7_): [M + H]^+^ = 499.2436 (499.2439).

### General procedure for the synthesis of compounds 3 and 4

The synthesis of the chelators DFO^*sp^ 3 and oxoDFO*4 was performed with an automated microwave-assisted peptide synthesizer by standard Fluorenylmethoxycarbonyl (Fmoc) chemistry. The reaction was carried out in a 10 ml vial at a scale of 0.06 mmol. The low-loading Rink amide MBHA (4-methylbenzylhydrylamine) resin (0.041 mmol/g loading, 100–200 mesh) was used as a solid support. The resin was swollen in DMF at 70 °C for 20 min. The cleavage of the Fmoc protecting group was accomplished using 2 × 4.5 ml 20% piperidine in DMF at RT for 3- and 10-min reaction time, respectively. After the Fmoc-deprotection, the resin was washed with DMF (2 × 4.5 ml). The monomer (2 equiv. of compound 19 for synthesis of compound 3 or 4 equiv. of compound 20 for synthesis of compound 4) was prepared in 0.1 M solutions in DMF. HATU (0.1 M in DMF, 2 equiv. for compound 3 or 4 Equiv. for compound 4) and DIPEA (0.1 M in DMF, 5 equiv.) were used as coupling reagents. Coupling reactions were carried out for 20 min at 60 °C. After the first coupling to the resin, a capping was carried out. Acetic anhydride (5 M in DMF, 80 equiv.) was added to the reaction vial for 10 min at room temperature. The coupling cycles (Fmoc deprotection, followed by coupling) were repeated three times. The last coupling was conducted as a double coupling. After the completion of the sequence, the final Fmoc was removed, and a washing step of the resin was carried out with DCM (3 × 3 ml). The cleavage from the resin and the deprotection of the chelator were performed using a microwave reactor in TFA/H_2_O/TIPS (38:1:1) for 1 h at 45 °C. The cleaved chelator was dried under a stream of argon, precipitated with ice-cold diethyl ether (Et_2_O, 1 ml), centrifuged, and decanted. After one additional wash with ice-cold Et_2_O followed by sonication, centrifugation and decanting, the crude product was isolated and analyzed by HPLC-MS (10–45% ACN/H_2_O over 6 min with 0.1% FA) and purified by preparative-HPLC (flow rate: 17 mL/min, mobile phase A = H_2_O + 0.1% FA, mobile phase B = ACN+0.1% FA, gradient: 0–5 min 10% B, 20.0 min 40% B, 20.5 min 95% B, 24.0 min 95% B, 24.5 min 10% B). After lyophilization of the combined fractions, products 3 and 4 were obtained as a white solid with a yield of 20% and 6.5% respectively.

Analysis for compound 3

^1^H NMR (600 MHz, DMSO-d6) δ 3.48 (m, 8 H), 3.01 (m, 6 H), 2.63 (m, 2 H), 2.58 (m, 6 H), 2.40 (m, 2 H), 2.28 (m, 8 H), 1.51 (m, 10 H), 1.39 (m, 6 H), 1.25 (m, 8 H) ppm.

^13^C NMR (151 MHz, DMSO-d6) δ 173.61, 171.95, 171.27, 47.05, 46.79, 38.39, 29.84, 29.49, 28.99, 28.79, 27.52, 27.41, 27.29, 26.68, 26.00, 23.47, 22.83 ppm.

ESI-HR-MS m/z found (calculated for C_36_H_67_N_9_O_12_): [M + H^+^]^+^ = 818.4982 (817.2439).

Analysis of compound 4 was in agreement with published data reported by Briand et al. (Briand et al. 2017)

### Complexation reaction with non-radioactive ^nat^Ga and ^nat^Zr

^nat^Ga-DFO 24, ^nat^Ga-DFO* 25, ^nat^Ga-DFO*^sp^ 26, ^nat^Ga-oxoDFO* 27.

Adapting a procedure reported by Jodal et al.,(Jodal et al. 2014) the chelators (1 mg, 1 eq.) was dissolved in 200 µL DMSO. 100 µL ammonium acetate buffer (0.5 M, pH 5.5) and Ga(NO₃)₃ (3 mg, 10 equiv.) were added. The reaction mixture was shaken for 60 min at RT (final pH = 5) and analyzed by HPLC-MS (5–95% ACN/H_2_O over 6 min with 0.1% FA). After full conversion, the mixture was lyophilized to give a white powder.

^nat^Ga-DFO 24: LC-LRMS m/z found (calculated for C_25_H_45_N_6_O_8_Ga): [M + H^+^]^+^ = 627.5 (626.3)

^nat^Ga-DFO* 25: LC-LRMS m/z found (calculated for C_34_H_60_N_8_O_11_Ga): [M + H^+^]^+^ = 827.5 (825.4)

^nat^Ga-DFO*^sp^ 26: LC-LRMS m/z found (calculated for C_36_H_63_N_9_O_12_Ga): [M + H^+^]^+^ = 884.8 (882.4)

^nat^Ga-oxoDFO* 27: LC-LRMS m/z found (calculated for C_32_H_55_N_9_O_16_Ga): [M + H^+^]^+^ = 892.7 (890.3)

^nat^Zr-DFO 28, ^nat^Zr-DFO* 29, ^nat^Zr-DFO*^sp^ 30, ^nat^Zr-oxoDFO* 31

Adapting a procedure reported by Patra et al.,(Patra et al. 2014) the chelators (1 mg, 2: 1.31 µmol, 3: 1.22 µmol, 4: 1.21 µmol, 1.0 equiv.) were dissolved in 100 µL DMSO. 100 µL water with 0.1% TFA and 1.95 mL of 0.1 M HCl were added (for oxoDFO*, the chelator was directly dissolved in 2 mL 0.1 M HCl). ^nat^ZrCl_4_ (3–4 mg, 2: 13.1 µmol, 3: 12.2 µmol, 4: 12.1 µmol, 10 eq.) was added, and the pH was adjusted to 7.5-8 using K_2_CO_3_ (0.1 M) to give a final chelator concentration between 0.55 and 0.6 nM. The reaction mixture was shaken for 60 min at RT and analyzed by HPLC-MS (5–95% ACN/H_2_O over 6 min with 0.1% FA). After the completion of the reaction, the mixture was lyophilized to give a white powder.

^nat^Zr-DFO 28 was prepared according to already published literature and the analysis corresponded to the already published data (Brandt et al. 2020).

^nat^Zr-DFO* 29: LC-LRMS m/z found (calculated for C_34_H_60_N_8_O_11_Zr): [M + H^+^]^+^ = 847.5 (846.3).

^nat^Zr-DFO*^sp^ 30: LC_LRMS m/z found (calculated for C_36_H_63_N_9_O_12_Zr): [M + H^+^]^+^ = 904.6 (903.4).

^nat^Zr-oxoDFO* 31: LC-LRMS m/z found (calculated for C_32_H_55_N_9_O_16_Zr): [M + H^+^]^+^ = 912.5 (911.3).

### ^68^Ga-radiolabeling

Stock solutions of DFO 1, DFO* 2, DFO*^sp^ 3, and oxoDFO* 4 were prepared by dissolving them in water (1 µg/µL, 1.21–1.78 nM). Each compound was radiolabeled as follows: 20 µL of DFO or 10 µL of each DFO-analog solution was mixed with 30 µL of sodium acetate (1.14 M, 155 mg/mL in water) and 300 µL of [^68^Ga]GaCl_3_ generator eluate (15 − 60 MBq) in 0.1 M HCl. The reaction mixture was incubated for 15 min at 95 °C, and then the pH was adjusted to 6–7 by adding 100 µL of sodium acetate (1.14 M, 155 mg/mL). The radiochemical purity of the radiolabeled siderophores was monitored by RP-HPLC and iTLC-SG. Chromatographic paper strips containing a sample of the [^68^Ga]Ga-DFO/DFO*/DFO*^sp^/oxoDFO* complex were developed in a chamber saturated with aq. diethylenetriamine pentaacetate (DTPA, 0.05 M) or sodium citrate dihydrate (0.1 M, pH 5). After development, the strips were scanned using a radiometric phosphor imager, and the chromatograms for each strip were evaluated. For all, DFO and analogs 5, 6, 7, 8, the Rf value was 0, whereas for the free [^68^Ga]Ga^3+^, the Rf was 0.9 (Figure S50), both when DTPA and citrate were used as the mobile phase.

### ^89^Zr-Radiolabeling

^89^Zr-labeling reactions were carried out by adapting published methods by Brandt et al. (Brandt et al. 2020). In brief, stock solutions of the chelators (1 mM) were prepared in water (oxoDFO* 4) or DMSO (chelators 1, 2, and 3). [^89^Zr]Zr-oxalate (approx. 10 MBq) was transferred to a LoBind Eppendorf^®^ flask and diluted with 0.1 M oxalic acid (pH 1) to a total volume of 10 µL. Na_2_CO_3_ (2.5 µL, 2 M) was added, and the mixture was left standing for 3 min (RT, pH 6–7). 10 µL of chelator stock solutions (10 nmol) was added to the neutralized solution of ^89^Zr. HEPES buffer (0.5 M, pH 7.4) was added to a final volume of 1 mL (resulting in final precursor concentrations of 2–50 µM), and the mixture was incubated at RT for up to 2 h. The progress of the reaction was monitored by iTLC and RP-TLC with citrate buffer (0.1 M, pH 4) as mobile phase. For all complexes, the rf was 0, while for the free [^89^Zr]Zr-oxalate, rf was 0.9 (Figure S51). Complexes 10, 11, and 12 were obtained with an RCY and RCP > 95%. The specific activity of complexes 10, 11, and 12 was calculated as 3.60 MBq µg^− 1^, 2.58 MBq µg^− 1^, and 2.55 MBq µg^− 1^, respectively.

### Log P determination of complexes

The partition coefficient (log P, *n* = 6) was determined by adding 350 µL of the [^68^Ga]Ga-DFO*-6/DFO*^sp^-7/oxoDFO*-8 radiolabeling reaction mixture to 650 µL phosphate-buffered saline (PBS, Sigma-Aldrich). A 50 µL sample was taken from this dilution and mixed with 450 µL PBS and 500 µL n-octanol. This solution was vortexed (1500 rpm, 2 min) and then centrifuged (1 min, 15,000 g) to separate the phases. A 50 µL sample was taken from the aqueous and organic phase and measured in a γ-counter. The phases for the partition coefficient determination experiments were measured with a gamma counter (2480 Wizard2 automatic gamma counter; PerkinElmer, Waltham, MA, USA). Log P was then calculated from the data (mean ± SD of *n* = 6).

After labeling with [^89^Zr]Zr-DFO-analogs, the solutions were supplemented with PBS (Sigma Aldrich) to 1 ml. The subsequent procedure was the same as that for [^68^Ga]Ga-labelled analogs.

### In vitro stability tests

Stability tests of ^68^Ga-complexes (*n* = 3 for 5, 6, 7, and 8) were performed by preparing samples: (1) 50 µL of the radiolabeling mixture 5, 6, 7, and 8 (described above) and 450 µL human serum (Sigma Aldrich); (2) 50 µL of the radiolabeling mixture 5, 6, 7, and 8 (described above) and 450 µL PBS (Sigma Aldrich). All mixtures were incubated at 37 °C for 30, 60, and 120 min after which time samples of 100 µL were taken. Samples of PBS solutions were analyzed directly by RP-HPLC whereas human serum samples were mixed with acetonitrile (200 µL), centrifuged (15,000 rpm, 3 min), and the supernatant was analyzed with RP-HPLC.

For ^89^Zr complexes 9, 10, 11, and 12 (*n* = 3), 50 µL of the radiolabeling mixture (described above) was added to 950 µL of human serum or PBS. Mixtures were incubated at 37 °C under shaking conditions. After 2 h, 24 h, and 48 h, 5 µL of the reaction picture were spotted on an Instant Thin Layer Chromatography paper impregnated with a silica gel. The TLC strip was then developed in a chamber saturated with aq. citrate buffer (0.1 M, pH 4). After development, the strips were scanned using a miniGITA Dual instrument. Stability was assessed via integration of the spectrum obtained and results are shown in Table 2. In cases where no clear quantification could be performed, the iTLC-SG strips were cut at approximately 3.5 cm from the elution front, and both halves of the strips were measured separately using a γ-counter.

### Protein binding

Plasma protein binding (*n* = 3 per compound) was determined by incubating 50 µL containing approximately 3 MBq of the [^68^Ga]Ga-DFO 5/DFO* 6/DFO*^sp^ 7/oxoDFO* 8 or approximately 5 MBq of [^89^Zr]Zr-DFO 9/DFO* 10/DFO*^sp^ 11/oxoDFO* 12 radiolabeling reaction mixture in 950 µL human serum (or 950 µL PBS as a control) at 37 °C for 30, 60, and 120 min. At each time, 25 µL of the sample was separated by spin filter analysis (molecular weight cut-off: 30 kDa) and centrifugation at 2000 g for 2 min. Protein binding of [^68^Ga]Ga- DFO*6/DFO*^sp^7/oxoDFO*8 was determined by measuring the activity of the spin filter (protein bound fraction) and the eluate (non-protein bound fraction) using a γ-counter. The procedure was repeated for mouse serum.

### Animal experiments

Animal experiments were performed with female balb/C mice, 10 to 12 weeks old (Envigo, Horst, The Netherlands). The animals were acclimatized to laboratory conditions for one week before the experiments and housed under standard laboratory conditions on sawdust in individually ventilated cages with free access to food and water. General health and body weight were monitored throughout the experiments. For all in vivo experiments, the number of animals was reduced as much as possible (generally *n* = 3 per group and time point). Injections and small animal imaging were all performed under 2% isoflurane anesthesia FORANE (Abbott Laboratories, Abbott Park, IL, USA) to minimize animal suffering and prevent animal movement. All animal experiments were done following the regulations and guidelines of the Czech Animal Protection Act (No. 246/1992) and with the approval of the Czech Ministry of Education, Youth, and Sports (MSMT-20283/2024-3 and MSMT-24421/2021-4) and the Institutional Animal Welfare Committee of the Faculty of Medicine and Dentistry of Palacky University in Olomouc.

### In vivo stability tests

balb/C mice under 2% isoflurane anesthesia were i. v. injected with [^68^Ga]Ga-DFO*-6/DFO*^sp^-7/oxoDFO*-8 at a dose of 5–10 MBq per animal (*n* = 3). Blood (ca. 50–100 µl) was obtained i. v. 5 min after injection and centrifuged at 5,000 rpm for 10 min to separate the plasma. The plasma was deproteinized by adding ACN, vortexed for 1 min, and centrifuged at 15,000 rpm for 5 min. The supernatant was collected for RP-HPLC analysis.

### Ex vivo biodistribution in balb/C mice

Biodistribution studies were performed in balb/C mice. Mice were r. o. injected with [^68^Ga]Ga-DFO 5/DFO* 6/DFO*^sp^ 7/oxoDFO* 8 (100 µl, 1 − 2 MBq, total approx. ∼0.5 µg of the chelators). All mice were sacrificed 0.5 and 1.5 h p.i. under general anesthesia by cervical dislocation, followed by exsanguination. Blood, spleen, pancreas, stomach, intestine, kidneys, liver, heart, lung, muscle, and bone were collected. The organs and tissues were weighed, and radioactivity was measured using the γ-counter. Biodistribution data were calculated as the percentage of injected dose per gram of tissue (% ID/g) (Fig. 2, SI Tables S3-S7).

Another group of mice received the [^89^Zr]Zr-DFO and DFO analogs 9, 10, 11, 12 (100 µl, 0.2 MBq, total approx. ∼0.5 µg of the chelators). The mice were sacrificed 1, 3, and 24 h p. i. and biodistribution data was collected as described above (Fig. 3, SI Tables S8 – S10).

### PET/CT imaging

The experimental animals (3 animals for ^68^Ga-labeled DFO-analogs and 3 animals for ^89^Zr-labeled DFO analogs, *n* = 1 per compound) under isoflurane anesthesia were injected r. o. with [^68^Ga]Ga-DFO*-6/DFO*^sp^-7/oxoDFO*-8 (110 µl, 5–10 MBq, approx. ∼0.5 µg of the chelators siderophore) and placed in the prone position in the small animal imaging system. After the administration of radiolabeled DFO-complexes, static imaging was initiated at 0.5 and 1.5 h p. i. for [^68^Ga]Ga-DFO-analogs and 1, 3, and 24 h p. i. for [^89^Zr]Zr-DFO-compounds. Mice were monitored by respiratory gating. Image reconstruction was performed using Mediso Tera-Tomo 3D PET iterative reconstruction (Mediso Medical Imaging Systems, Budapest, Hungary). Images were visualized, processed, and quantified using Mediso InterView FUSION (Mediso Medical Imaging Systems, Budapest, Hungary). Final images were normalized to injected activity and animal weight.

## Supplementary Information

Below is the link to the electronic supplementary material.

Supplementary Material 1: Additional file 1

## Data Availability

The datasets supporting the conclusions of this article are included within this article and in additional file 1. Further inquiries can be directed to the corresponding authors.

## Abbreviations

^68^Ga: Gallium-68
^89^Zr: Zirconium-89
ACN: Acetonitrile
Bn: Benzyl
CT: Computed tomography
DCM: Dichloromethane
DFO: Desferrioxamine-B, desferal
DIPEA: N,N-diisopropylethylamine
DMF: Dimethylformamide
DMSO: Dimethyl sulfoxide
DOTA 1,4,7,10: Tetraazacyclododecane-1,4,7,10-tetraacetic acid
DTPA: Diethylenetriaminepentaacetic acid
ESI-HR-MS: Electrospray ionization high-resolution mass spectrometry
FA: Formic acid
Fmoc: Fluorenylmethoxycarbonyle
HATU: Hexafluorophosphate azabenzotriazole tetramethyl uronium
HEPES: 4-(2-hydroxyethyl)-1-piperazineethanesulfonic acid
HRMS: High-resolution mass spectroscopy
i. v.: intravenously
iTLC-SG: instant thin layer chromatography with silica gel
K_2_CO_3_: Potassium carbonate
LC-MS: High-pressure liquid chromatography coupled to mass spectrometry
log *P*: the partition coefficient
MIP: Maximum intensity projection
NMR: Nuclear magnetic resonance
p.i.: post injection
PBS: Phosphate-buffered saline
PET: Positron emission tomography
Rf: Retention factor
r. o.: retro-orbitally
RCP: Radiochemical purity
RCY: Radiochemical yield
RM: Radiometal
RP-HPLC: Reversed-phase high-pressure liquid chromatography
RT: Room temperature
r_t_: retention time
SD: Standard deviation
sp: solid phase
SUV: Standardized uptake value
t_1/2_: half-life
tBu: tertbutyl
TFA: Trifluoroacetic acid
