# Supplementary material for: Gallium-68 and zirconium-89 complexes of DFO-analogs for molecular imaging applications using PET/CT
Source: EJNMMI Radiopharm Chem. 2026 Oct 23;11:80. doi: 10.1186/s41181-026-00509-0 (PMC13710496; doi:10.1186/s41181-026-00509-0)
Supplement: Supplementary file 1 [file 41181_2026_509_MOESM1_ESM.pdf]

Supporting Information for

Gallium-68 and Zirconium-89 complexes of DFO-analogs for molecular imaging by PET/CT

Robin A. J. Maradan[1, 2, 3 ǂ], Barbora Neuzilova[4 ǂ], Julia Kronberger[1, 2, 3], Irene Feiner[1, 5], Xabier Guarrochena[1, 2], Ayfer Y. Kılçar[6], Jan Ralis[7], Milos Petrik*[4, 8,9], Thomas L. Mindt*[1, 3, 5]

[1] Institute of Inorganic Chemistry, Faculty of Chemistry, University of Vienna, Josef-Holaubek-Platz 2 and Währinger Straße 42, 1090 Vienna, Austria

[2] Vienna Doctoral School in Chemistry, University of Vienna, Währinger Straße 42, 1090 Vienna, Austria

[3] Joint Applied Medicinal Radiochemistry Facility, University of Vienna, Währinger Straße 42, 1090, Vienna, Austria, and Medical University of Vienna, Spitalgasse 23, 1090, Vienna, Austria

[4] Institute of Molecular and Translational Medicine, Faculty of Medicine and Dentistry, Palacký University, Olomouc, Czech Republic

[5] Ludwig Boltzmann Institute Applied Diagnostic, General Hospital of Vienna, Währinger Gürtel 18-20, 1090 Vienna, Austria

[6] Institute of Nuclear Sciences, Ege University, Bornova–Izmir, 35100, Türkiye

[7] Nuclear Physics Institute of the Czech Academy of Sciences, Rez, Czech Republic

[8] Czech Advanced Technology and Research Institute, Palacký University, Olomouc, Czech Republic

[9] Institute of Molecular and Translational Medicine, University Hospital, Olomouc, Czech Republic

[ǂ] Equally contributing authors

Corresponding authors: Milos Petrik, e-mail: milos.petrik@upol.cz ; Thomas L. Mindt, e-mail: thomas.mindt@univie.ac.at

Table of contents

Analytical spectrum (NMR and HRMS) and chromatograms (LC-MS) of synthesized compounds (**3**, **17**, **18**, **19**, **20**, **21**, **24**, **25**, **26**, **27**, **28**, **29**, **30**, **31**, **32**, and **34**). 3

Hydroxylimine **32** 3

Hydroxylamine **17** 6

Monomer **19** 9

Dihydrooxazine **21** 13

Hydroxylimine **34** 21

Hydroxylamine **18** 23

Oxo-monomer **20**. 25

DFO*^{sp} **3**. 29

^{nat}Ga-DFO **24**. 34

^{nat}Ga-DFO* **25**. 34

^{nat}Ga-DFO*^{sp} **26**. 34

^{nat}Ga-oxoDFO* **27**. 35

^{nat}Zr-DFO **28**. .... **Error! Bookmark not defined.**

^{nat}Zr-DFO* **29**. 35

^{nat}Zr-DFO*^{sp} **30**. 35

^{nat}Zr-oxoDFO* **31**. 36

In vitro characterization of [^{68}Ga]Ga- and [^{89}Zr] Zr-DFO-analogs **5**, **6**, **7**, **8**, **9**, **10**, **11**, and **12**. 36

Ex vivo biodistribution study of [^{68}Ga]Ga- and [^{89}Zr]Zr-DFO-analogs **5**, **6**, **7**, **8**, **9**, **10**, **11**, and **12**. 46

Analytical spectrum (NMR and HRMS) and chromatograms (LC-MS) of synthesized compounds (**3**, **17**, **18**, **19**, **20**, **21**, **24**, **25**, **26**, **27**, **28**, **29**, **30**, **31**, **32**, and **34**).

Hydroxylimine **32**.

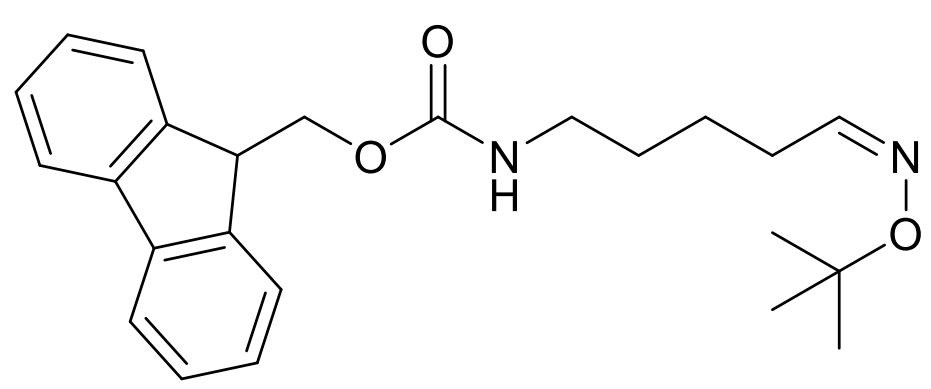

Figure S1: Structure of hydroxylimine ***32****.*

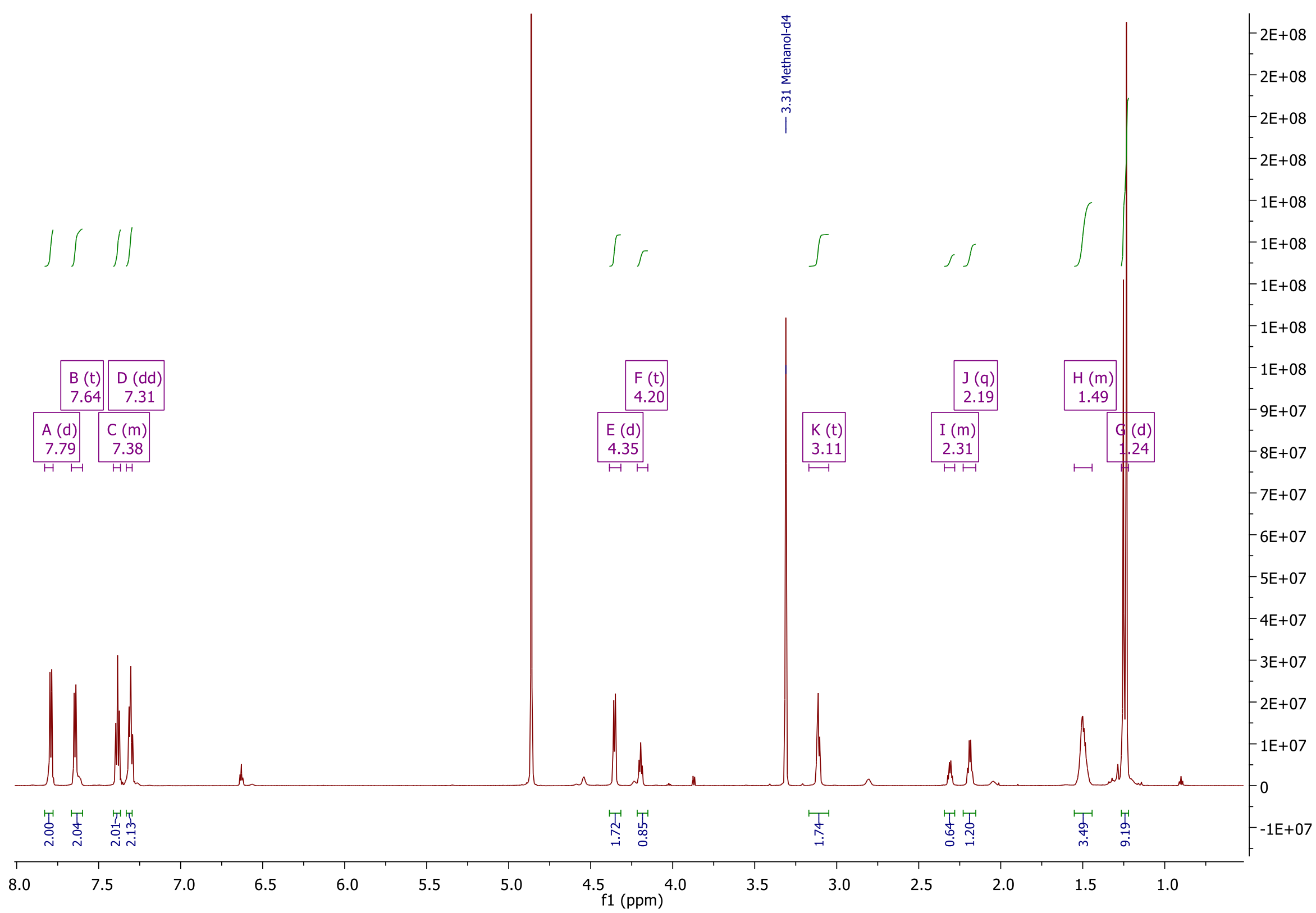

Figure S2: ¹H-NMR (700.4 MHz, Methanol-d4) spectrum of hydroxylimine ***32****.*

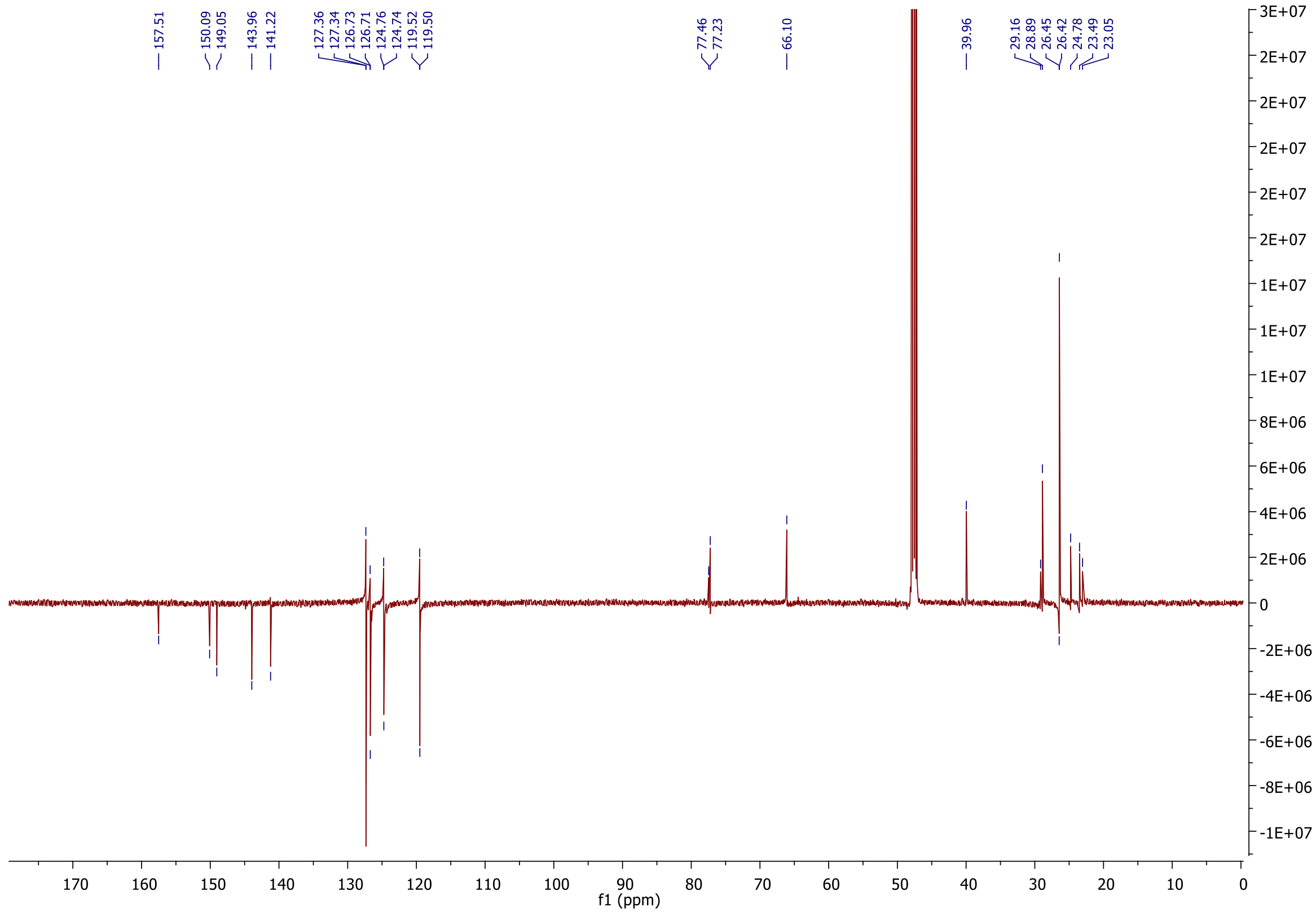

*Figure S3: ^{13}C-DEPT-NMR (176 MHz, Methanol-d4) spectrum of hydroxylimine **32**.*

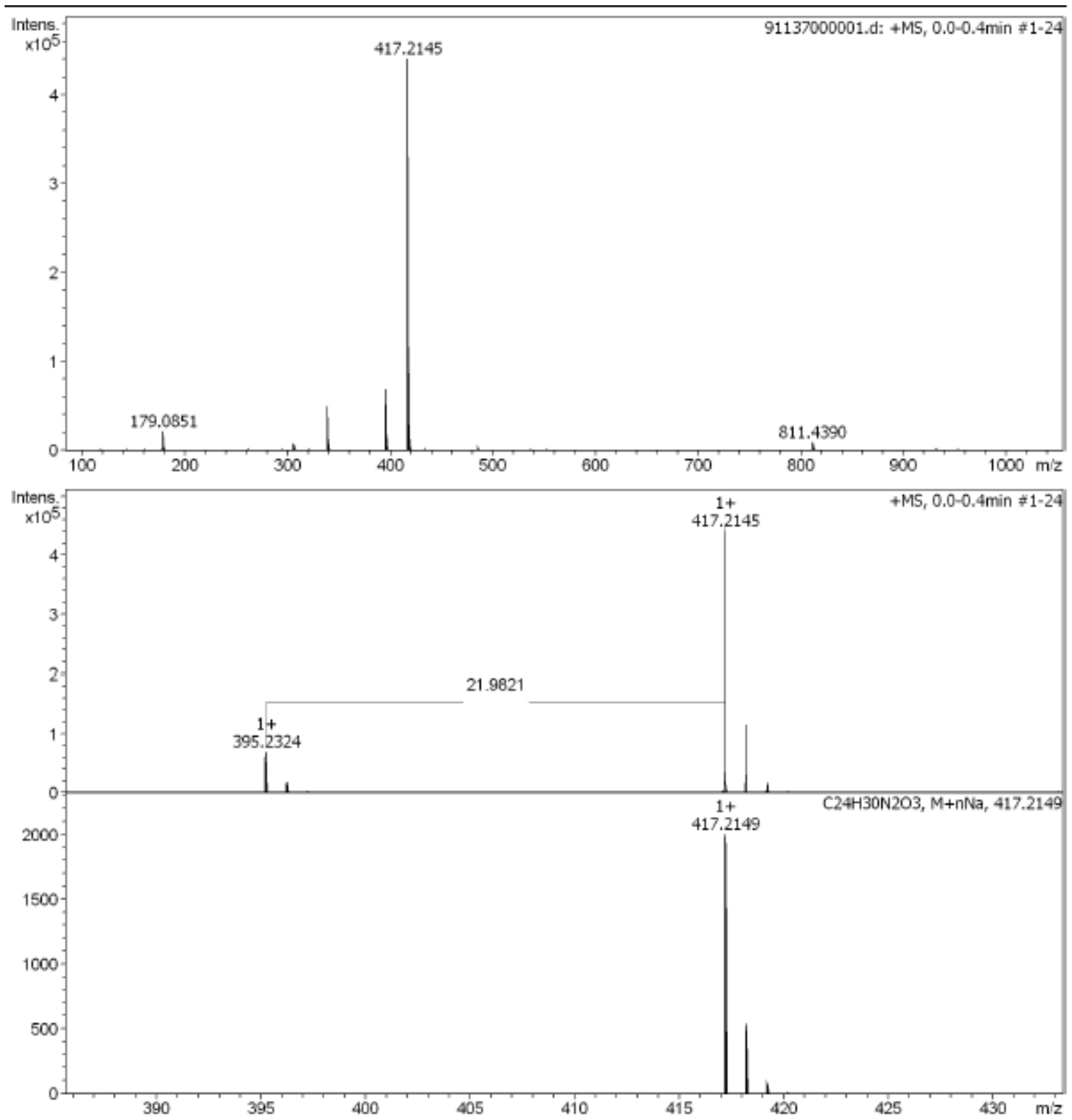

Figure S4: HRMS spectrum of hydroxylimine ***32****.*

Hydroxylamine **17**.

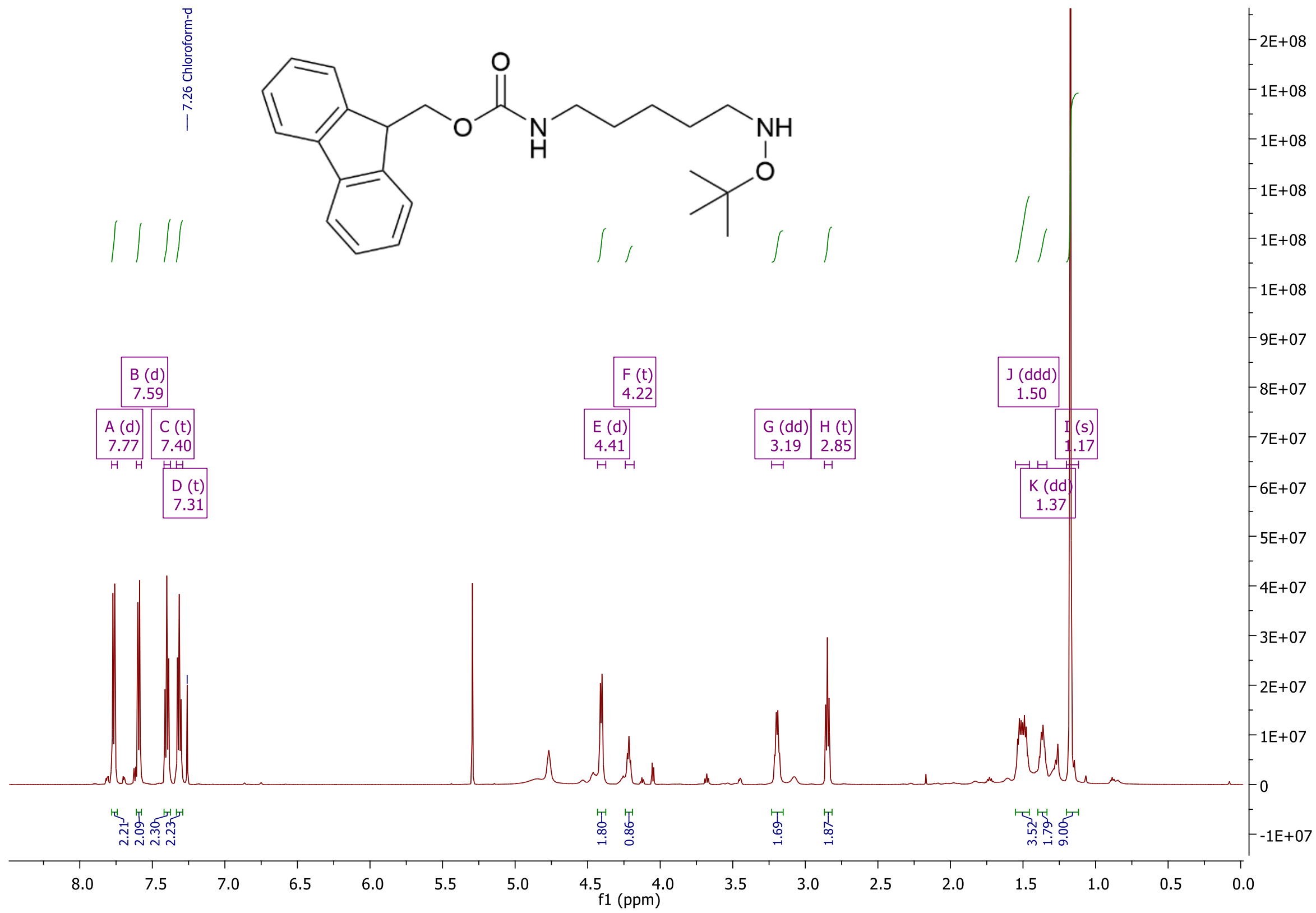

*Figure S5: ¹H-NMR (600.3 MHz, Chloroform-d) spectrum of hydroxylamine **17**.*

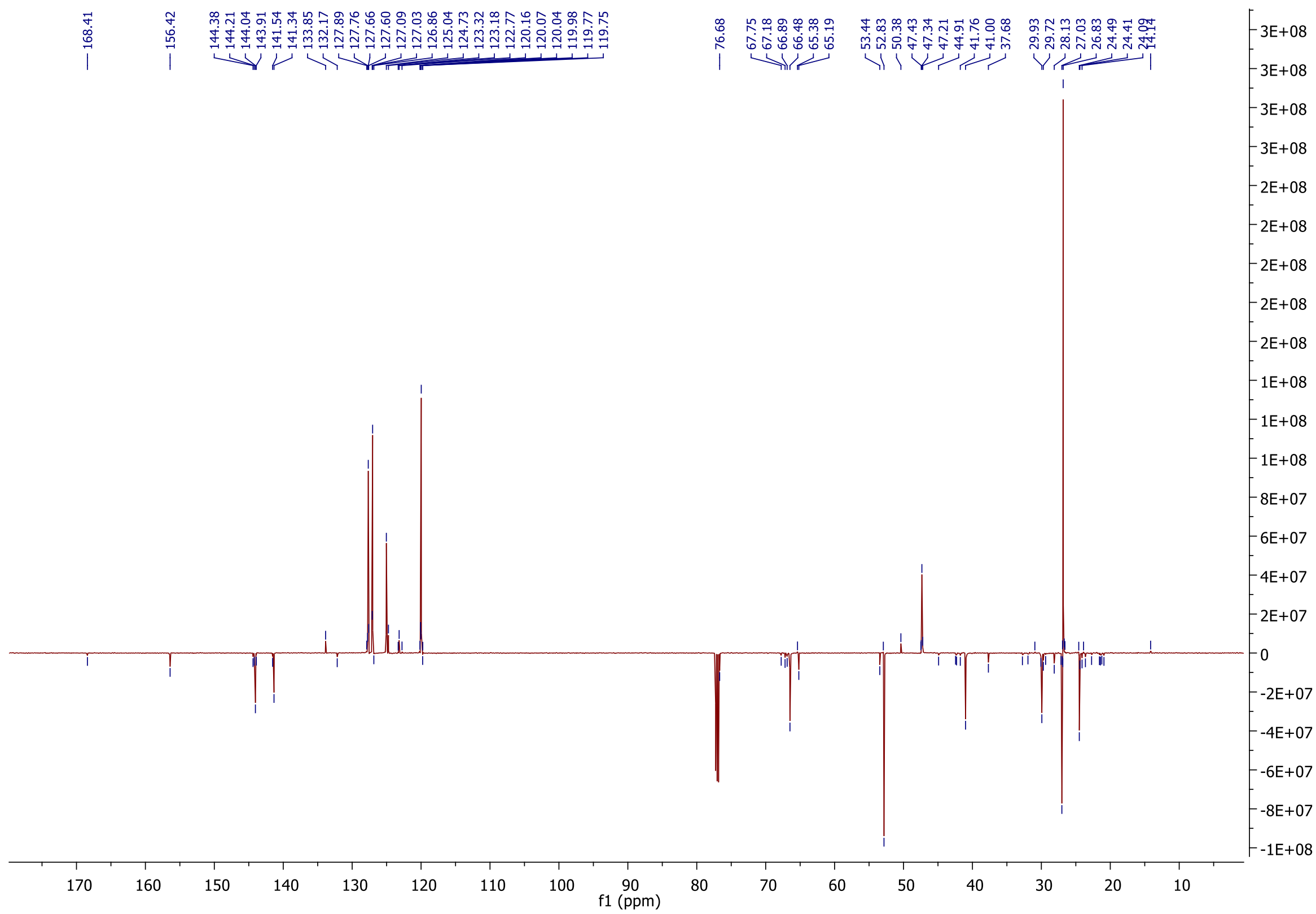

Figure S6: ^{13}C-DEPT-NMR (151 MHz, Chloroform-d) spectrum of hydroxylamine ***17***

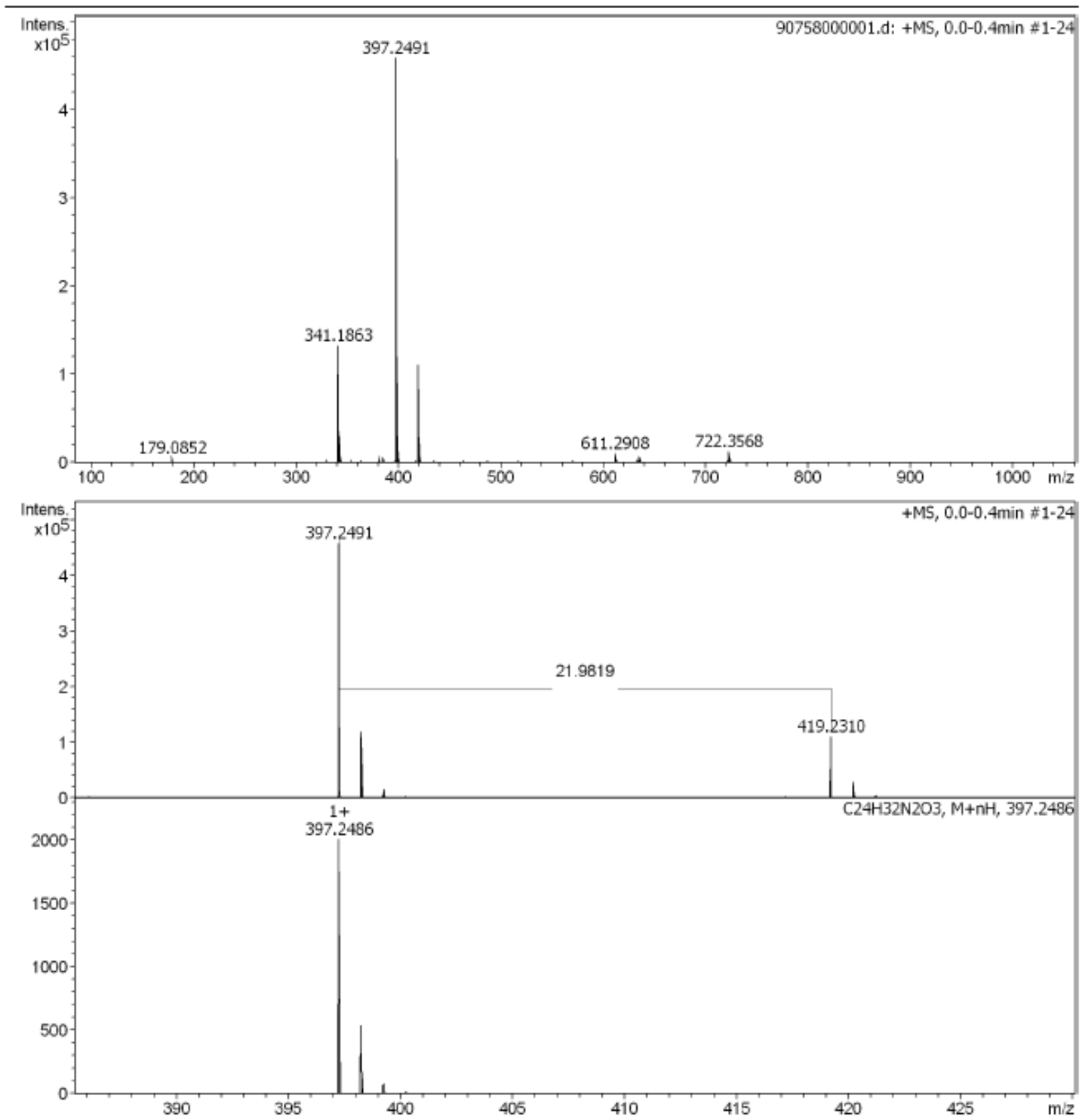

*Figure S7: HRMS spectrum of hydroxylamine **17**.*

Monomer **19**.

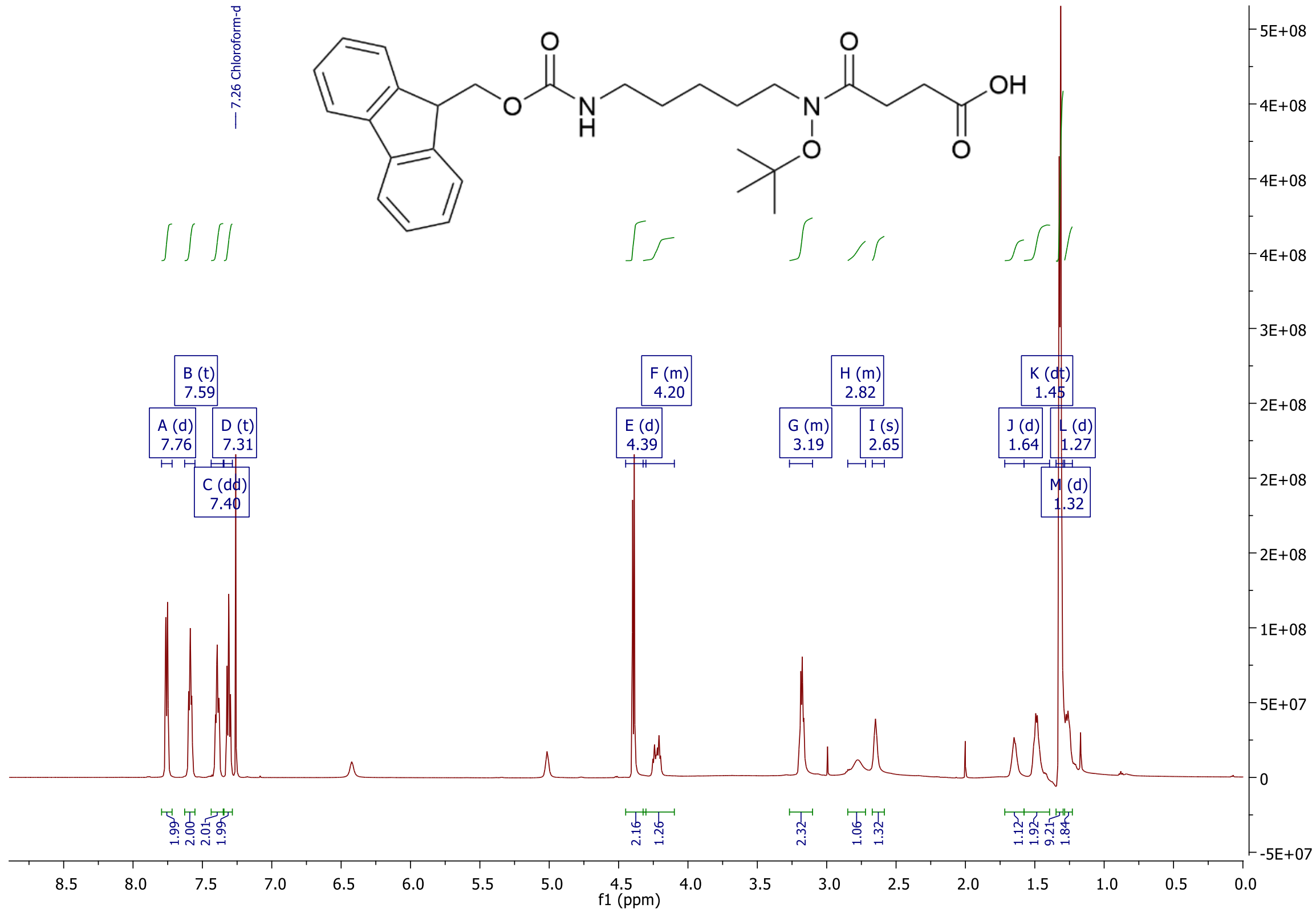

*Figure S8: ^{1}H-NMR (600.2 MHz, Chloroform-d) spectrum of monomer **19***

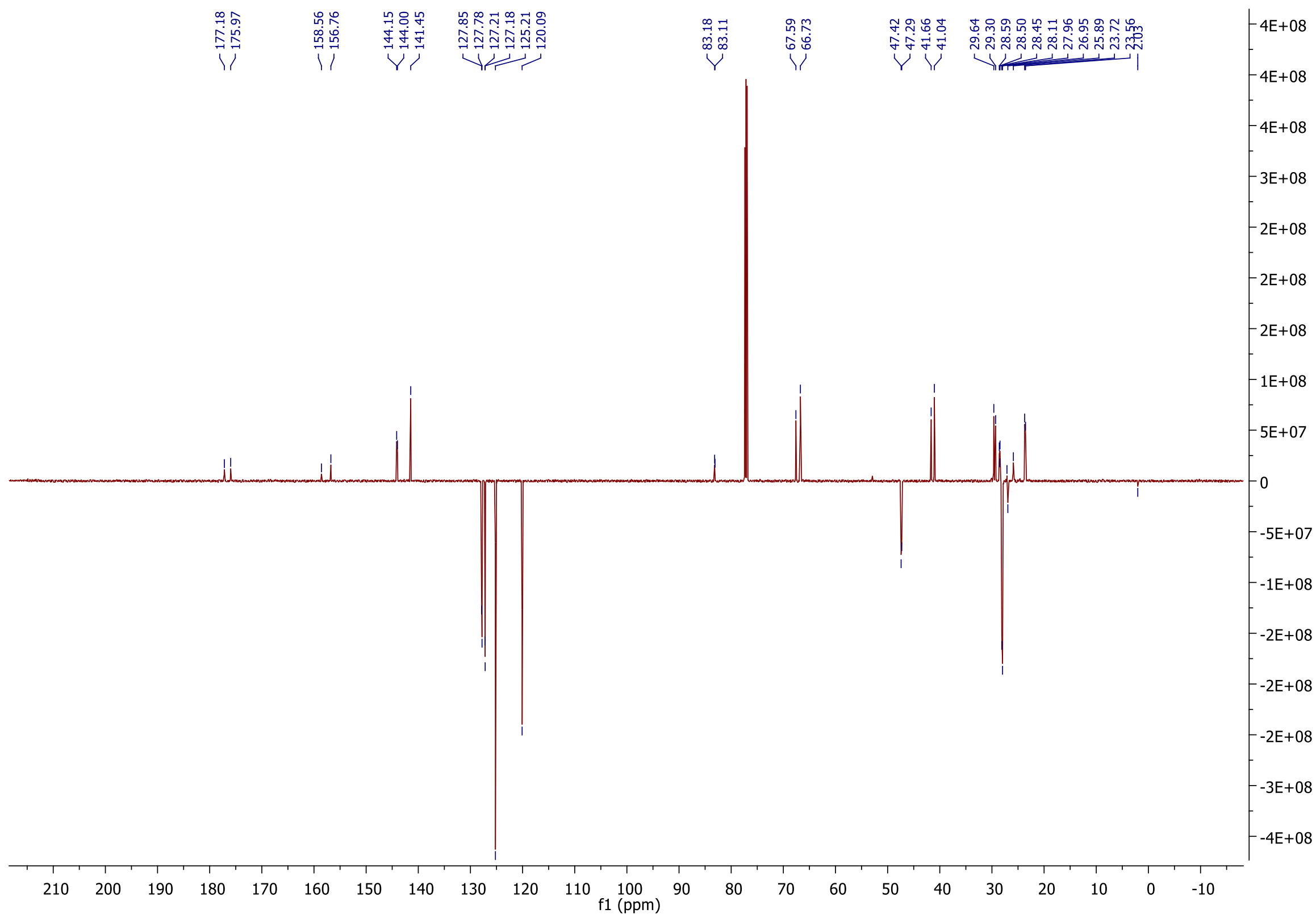

*Figure S9: ^{13}C-DEPT-NMR (151 MHz, Chloroform-d) spectrum of monomer **19**.*

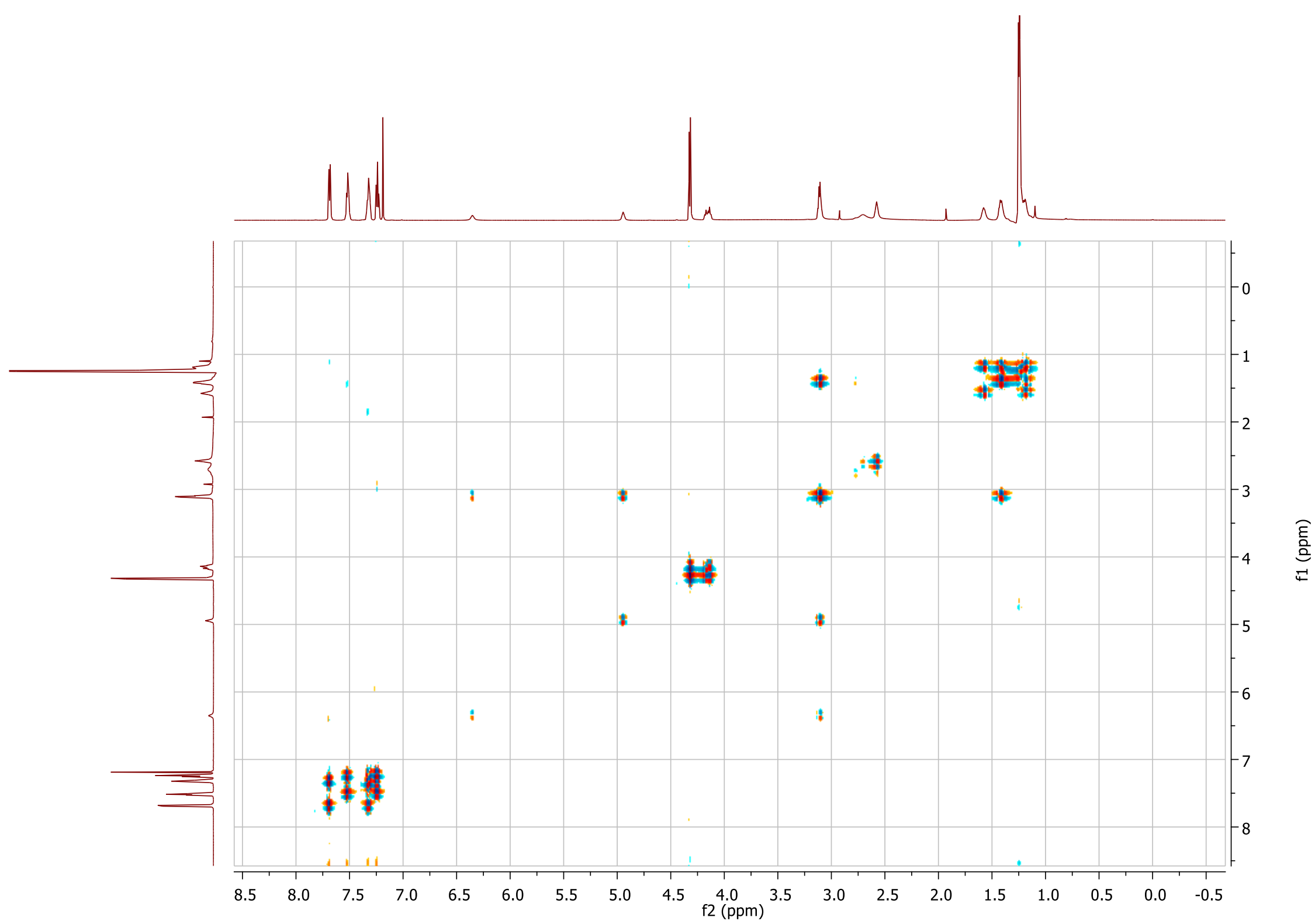

*Figure S10: COSY-NMR (Chloroform-d) of monomer **19**.*

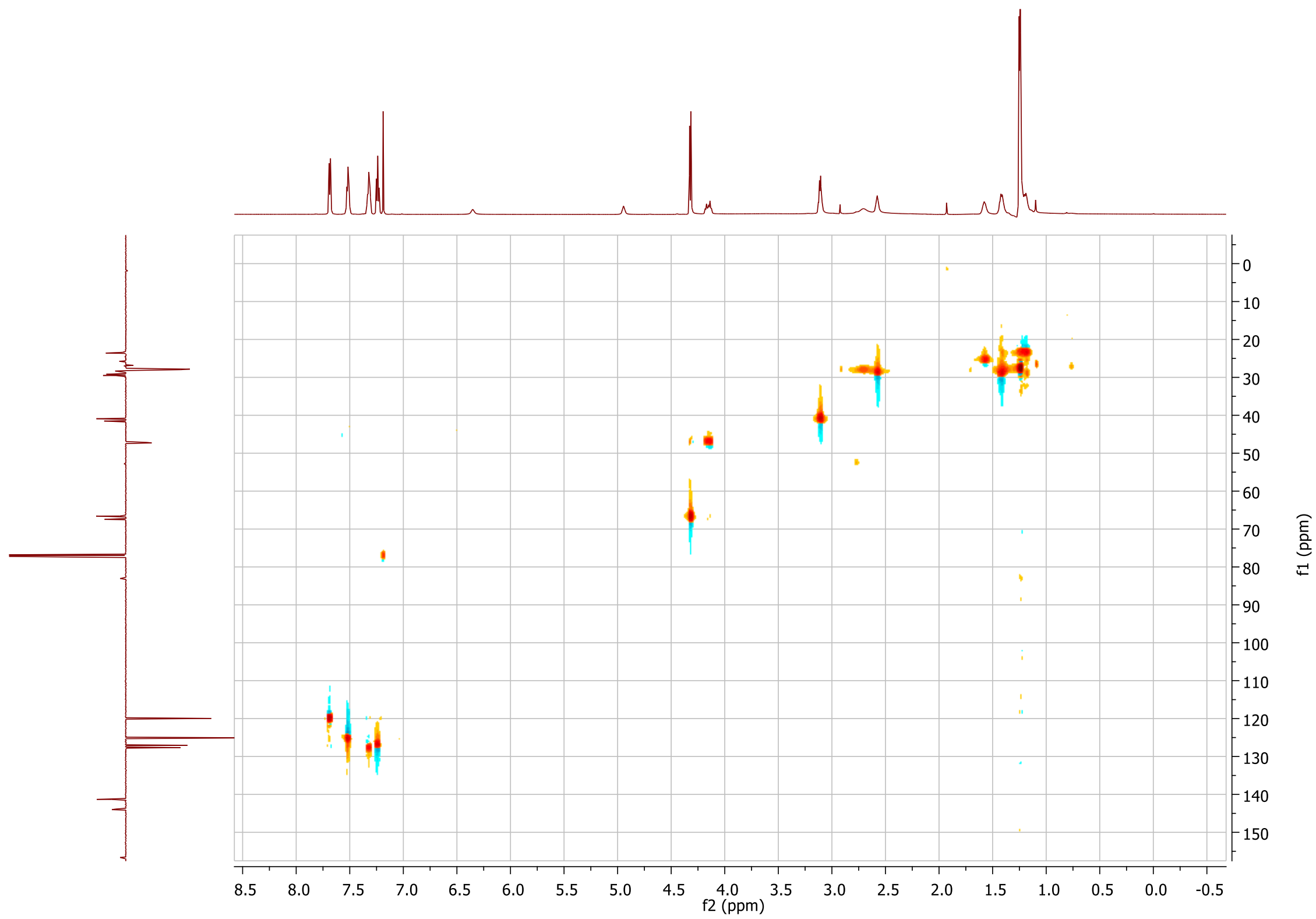

*Figure S11: HSQC-NMR (Chloroform-d) of monomer **19**.*

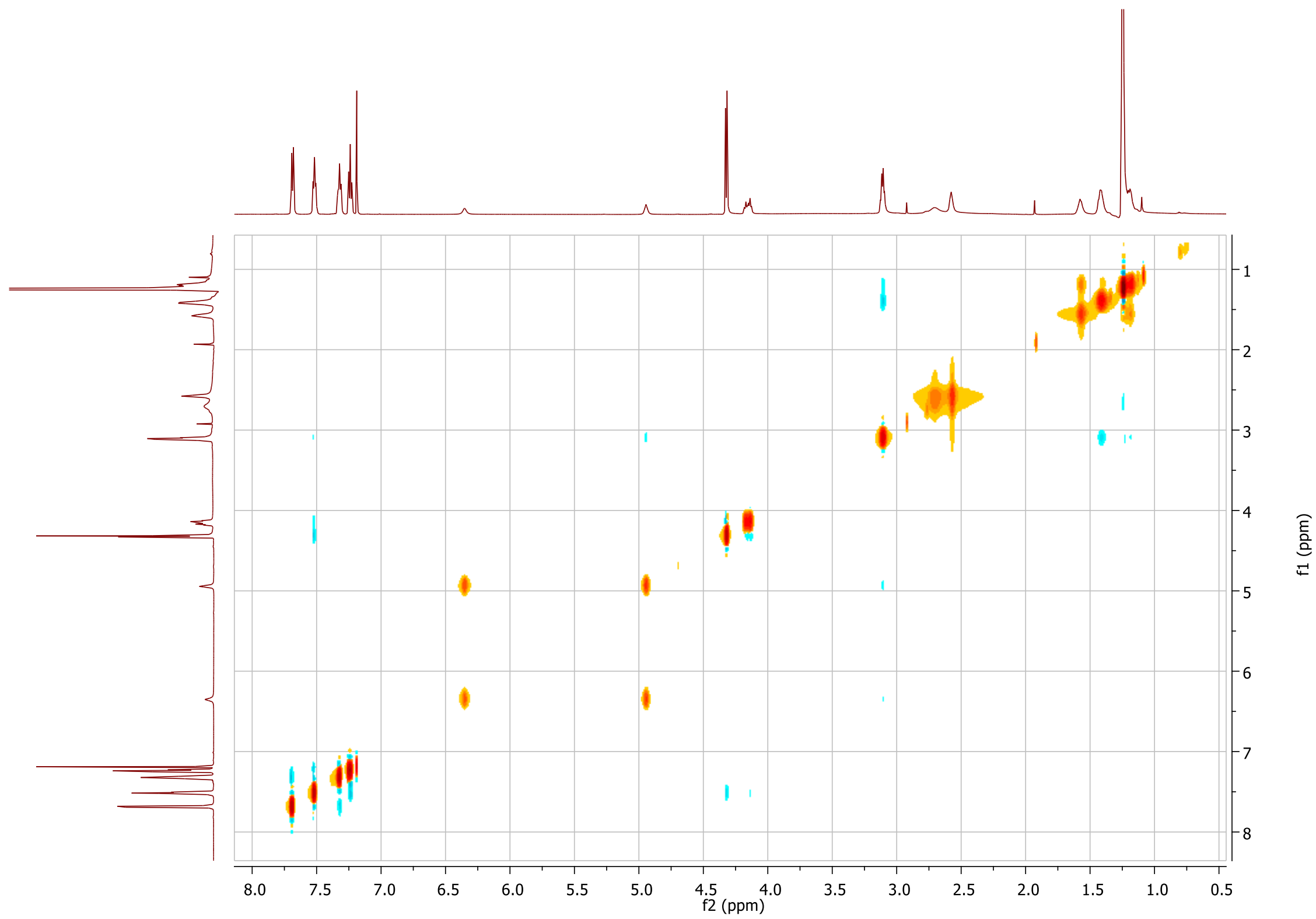

*Figure S12: NOESY-NMR (Chloroform-d) spectrum of monomer **19**.*

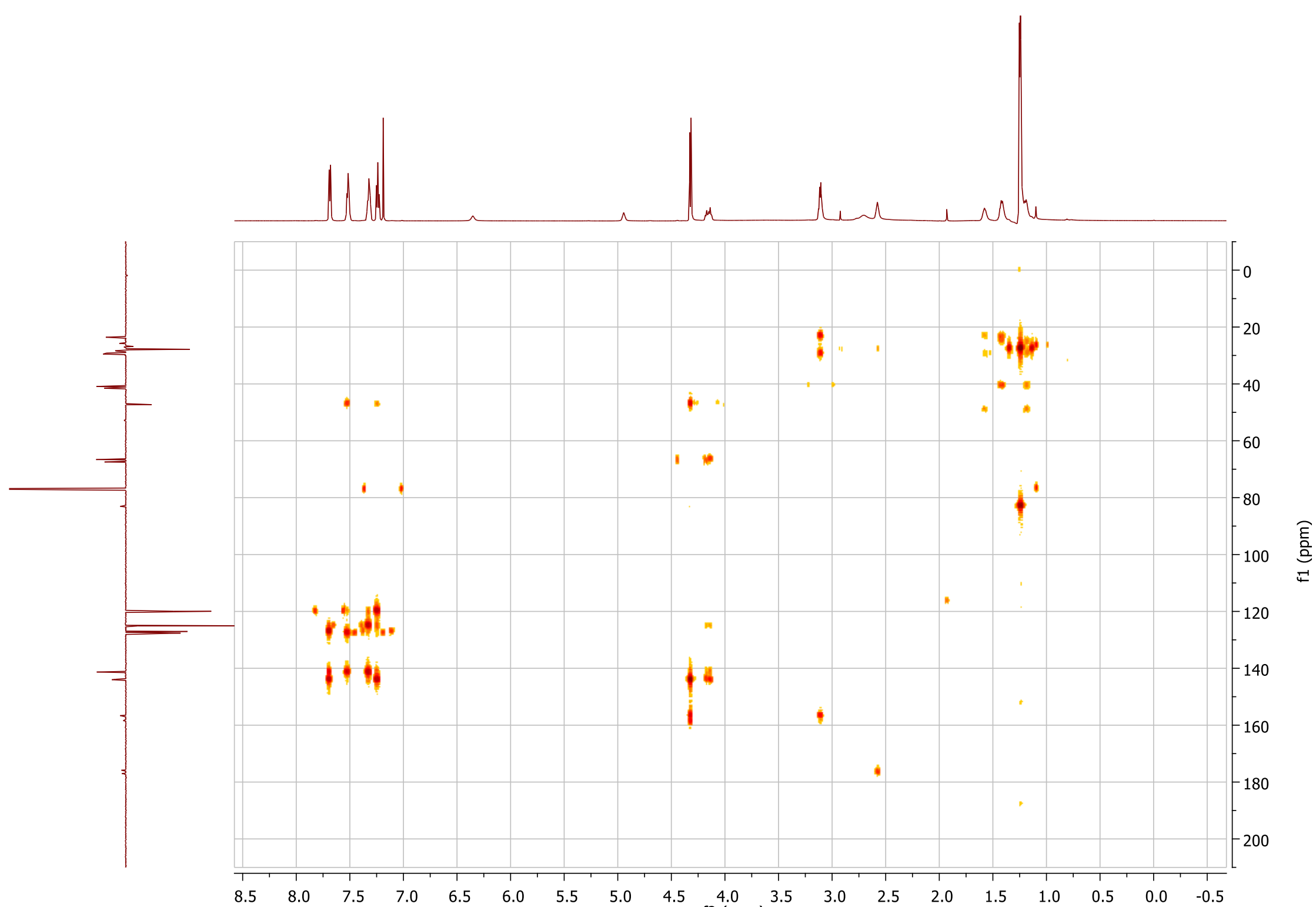

*Figure S13: HMBC-NMR (Chloroform-d) spectrum of monomer **19**.*

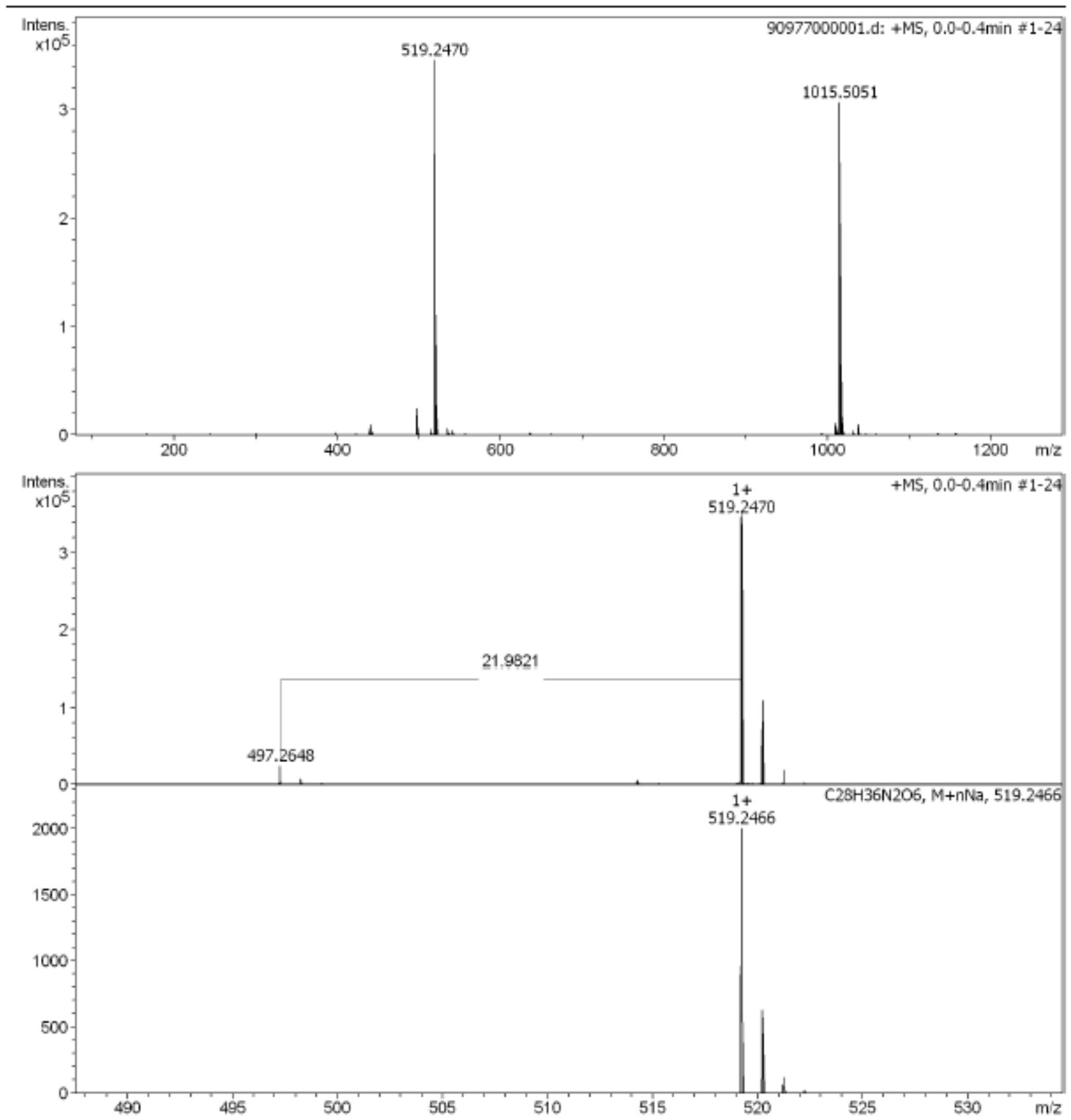

Figure S14: HRMS spectrum of Fmoc-mon(tBu) ***19****.*

Dihydrooxazine 21.

Note: The NMR analysis of this side product couldn't be done at room temperature due to the presence of isomers around the Nitrogen atom. Due to this, the signals of the carbon-carbon double bond were doubled (see Figure S16) to overcome this issue and obtain an unambiguous NMR analysis, measurement had to be performed at 100 °C (see Figure S15).

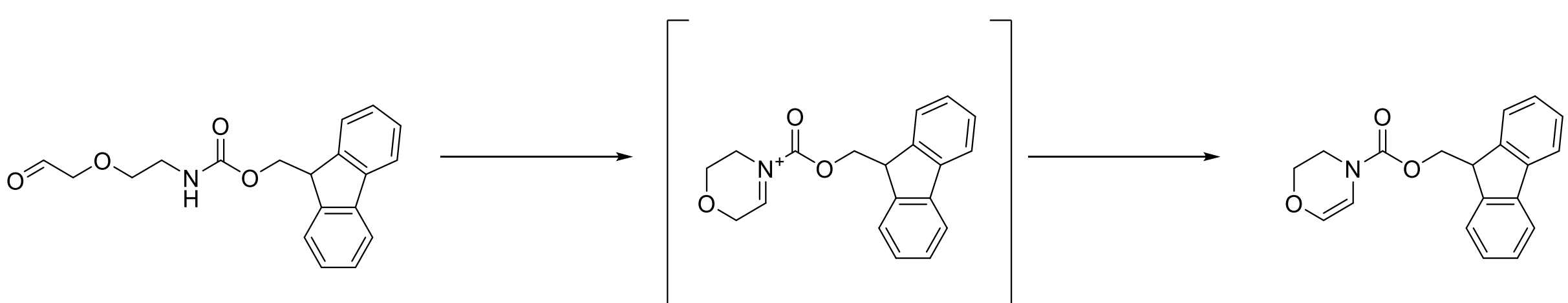

16 **33** **21**

Scheme S1: Proposed mechanism for the formation of dihydrooxazine ***21*** *from compound* ***16****.*

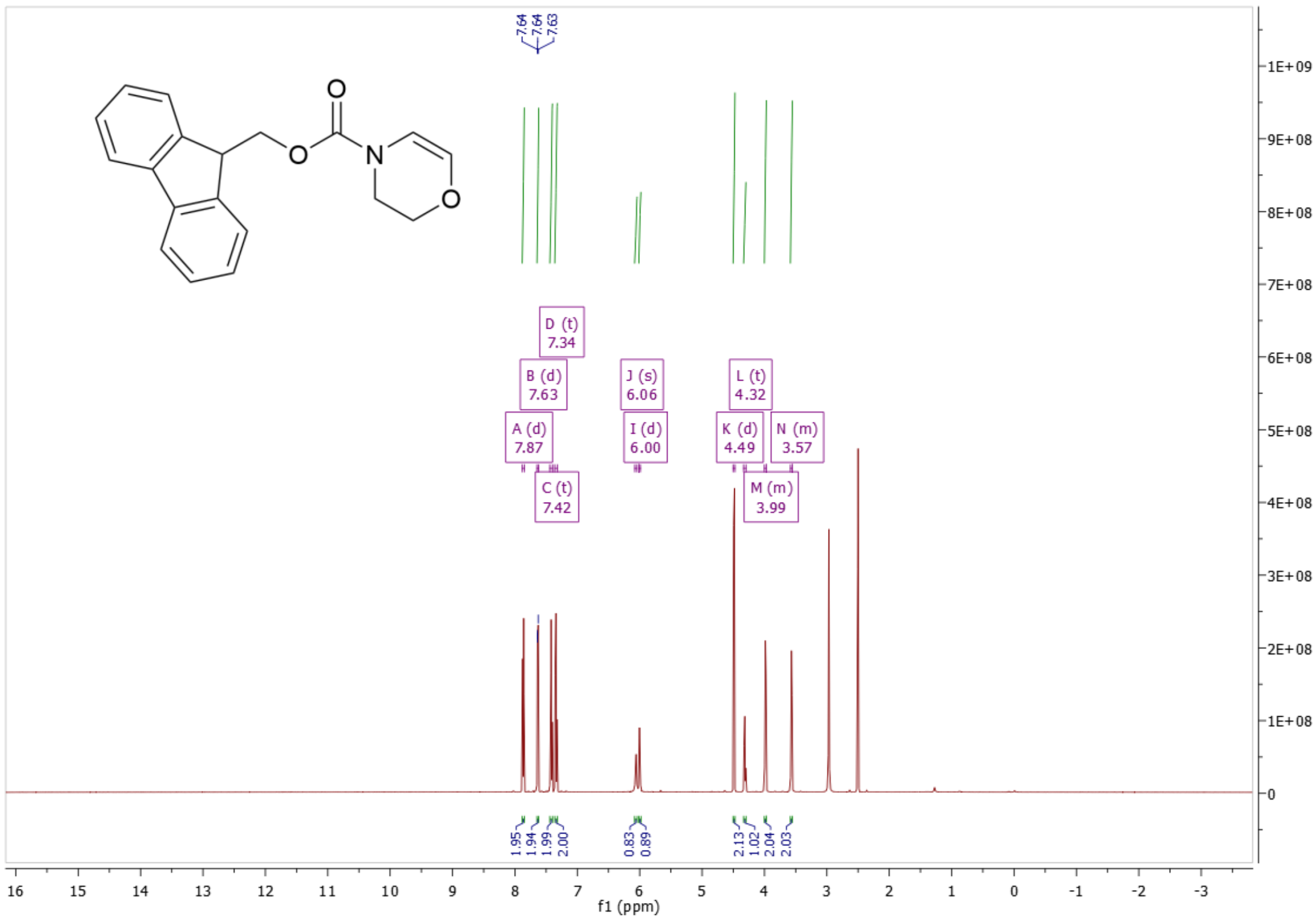

*Figure S15: ^{1}H-NMR (500.1 MHz, DMSO-d6) spectrum of dihydrooxazine **21**. Note: To avoid the doubling of the signal of the carbon-carbon double bond, this compound was measured at 100 °C.*

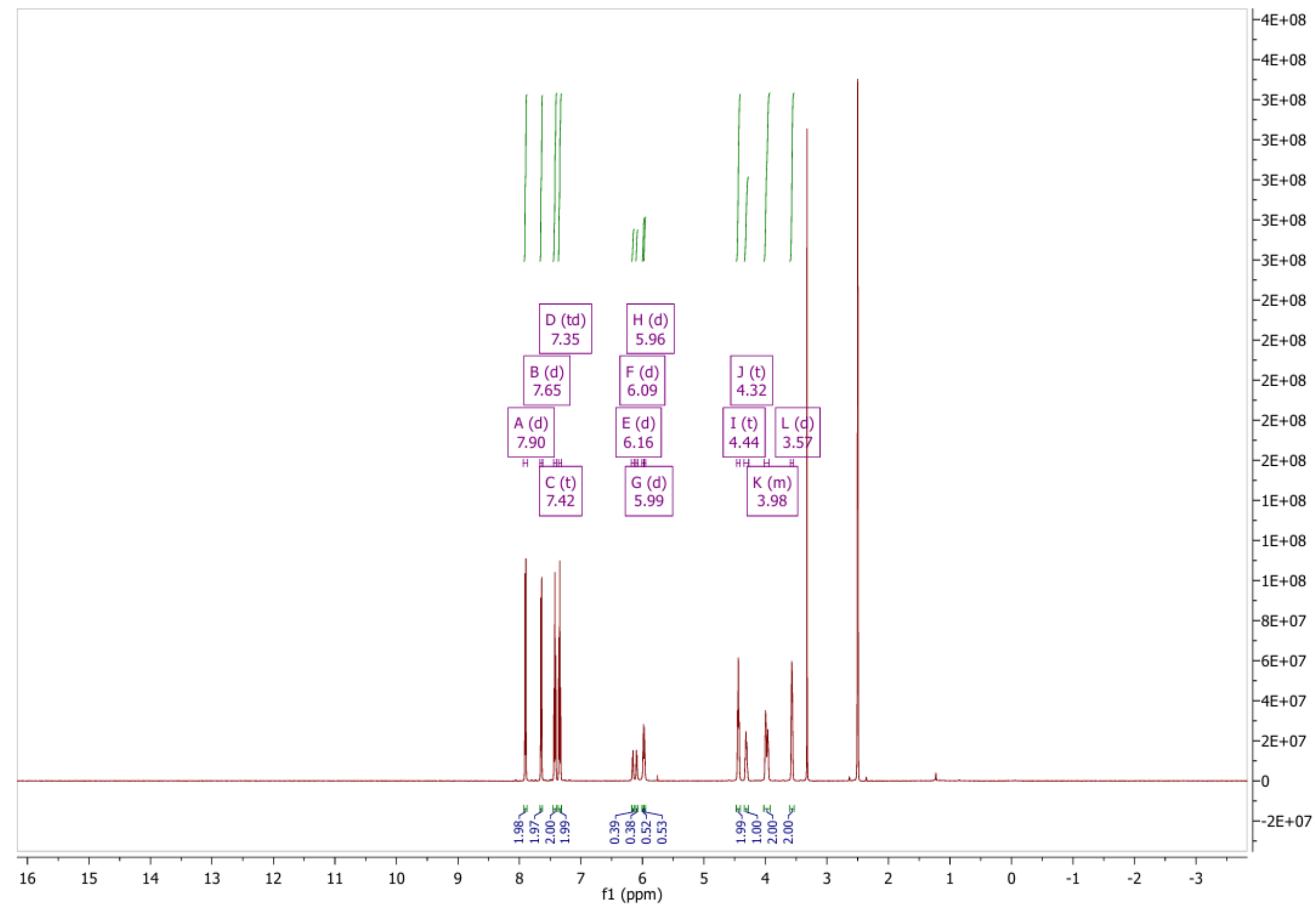

*Figure S16: ¹H-NMR (500.1 MHz, DMSO-d6) spectrum of dihydrooxazine **21**. Note: This spectrum was measured at room temperature. It can be noted that the signals between 5.9 and 6.2 ppm are doubled compared to the measurement at 100 °C.*

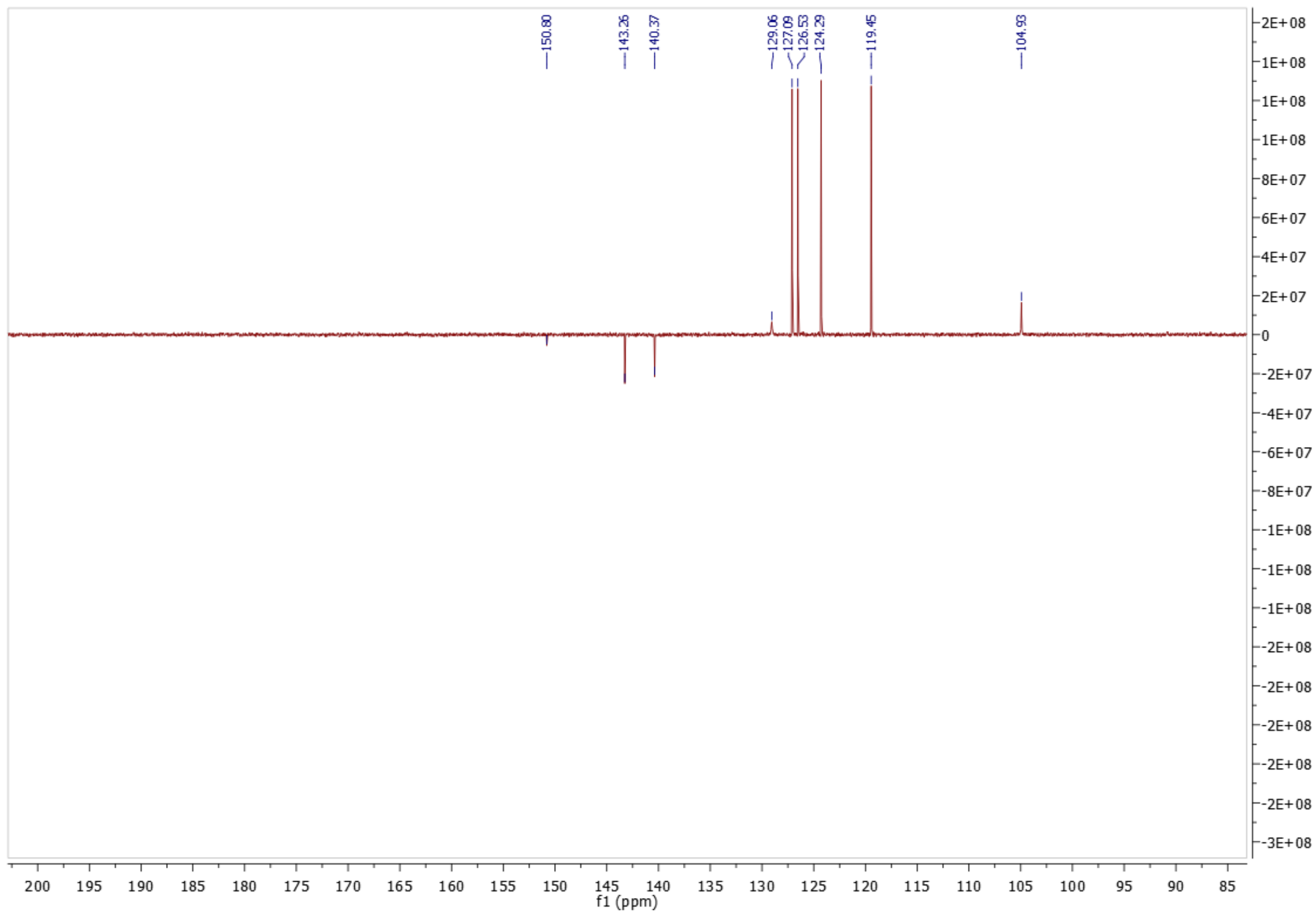

*Figure S17: ^{13}C-NMR (126 MHz, DMSO-d6) spectrum of dihydrooxazine **21**. Note: To avoid the doubling of the signal of the carbon-carbon double bond, this compound was measured at 100°C.*

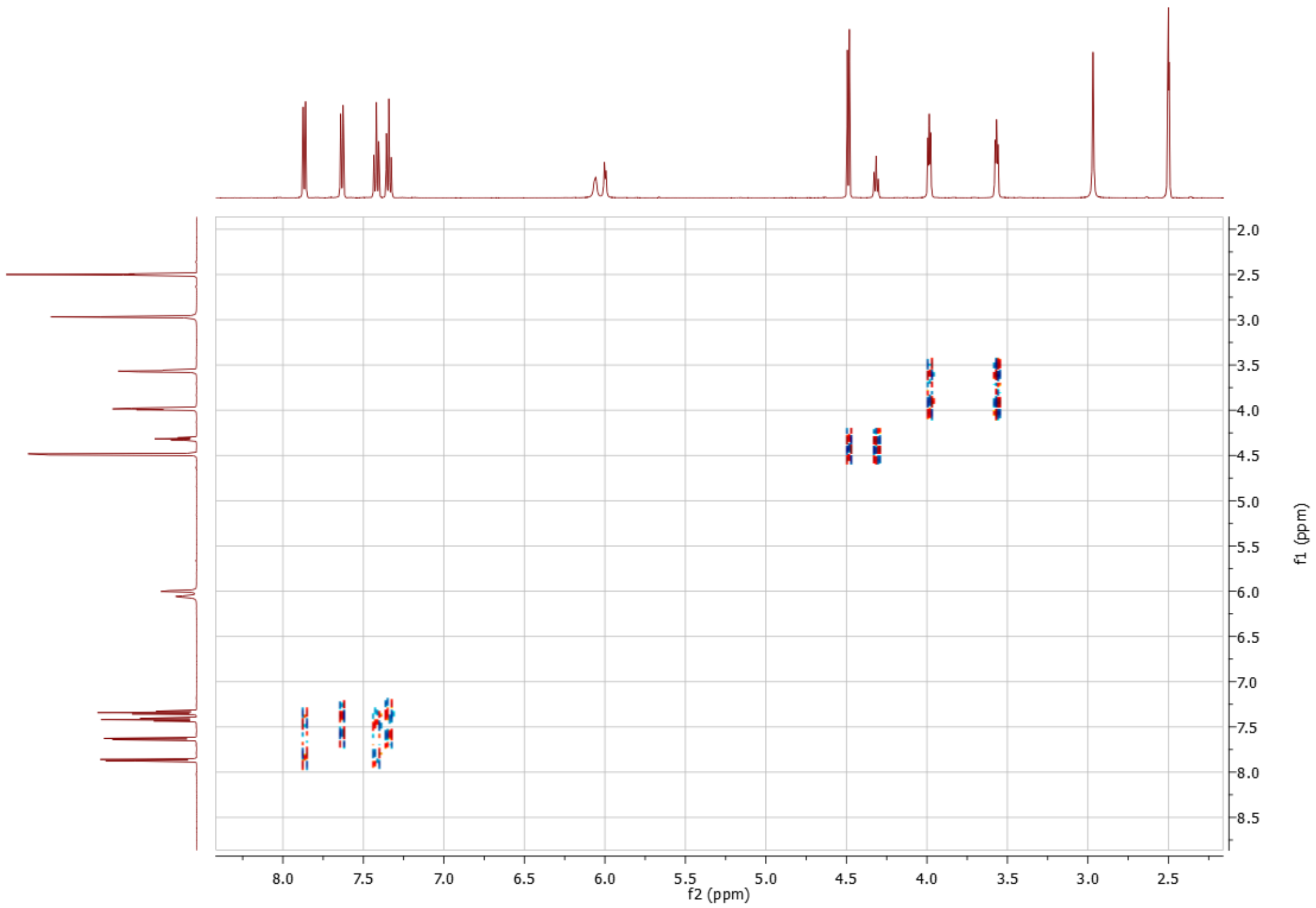

Figure S18: COSY-NMR (DMSO-d6) spectrum of dihydrooxazine ***21****. Note: To avoid the doubling of the signal of the carbon-carbon double bond, this compound was measured at 100°C.*

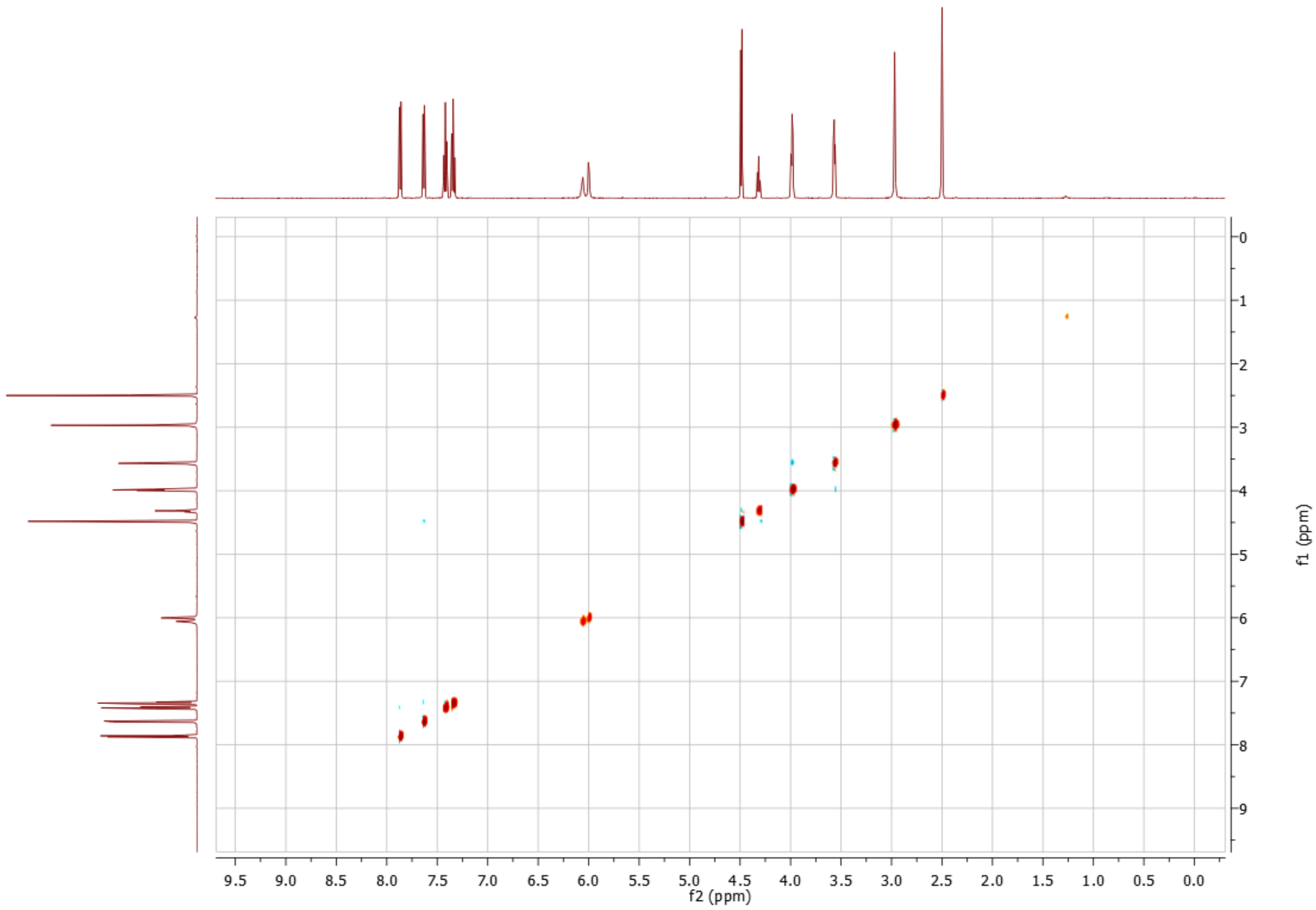

*Figure S19: NOESY-NMR (DMSO-d6) spectrum of dihydrooxazine **21**. Note: To avoid the doubling of the signal of the carbon-carbon double bond, this compound was measured at 100°C.*

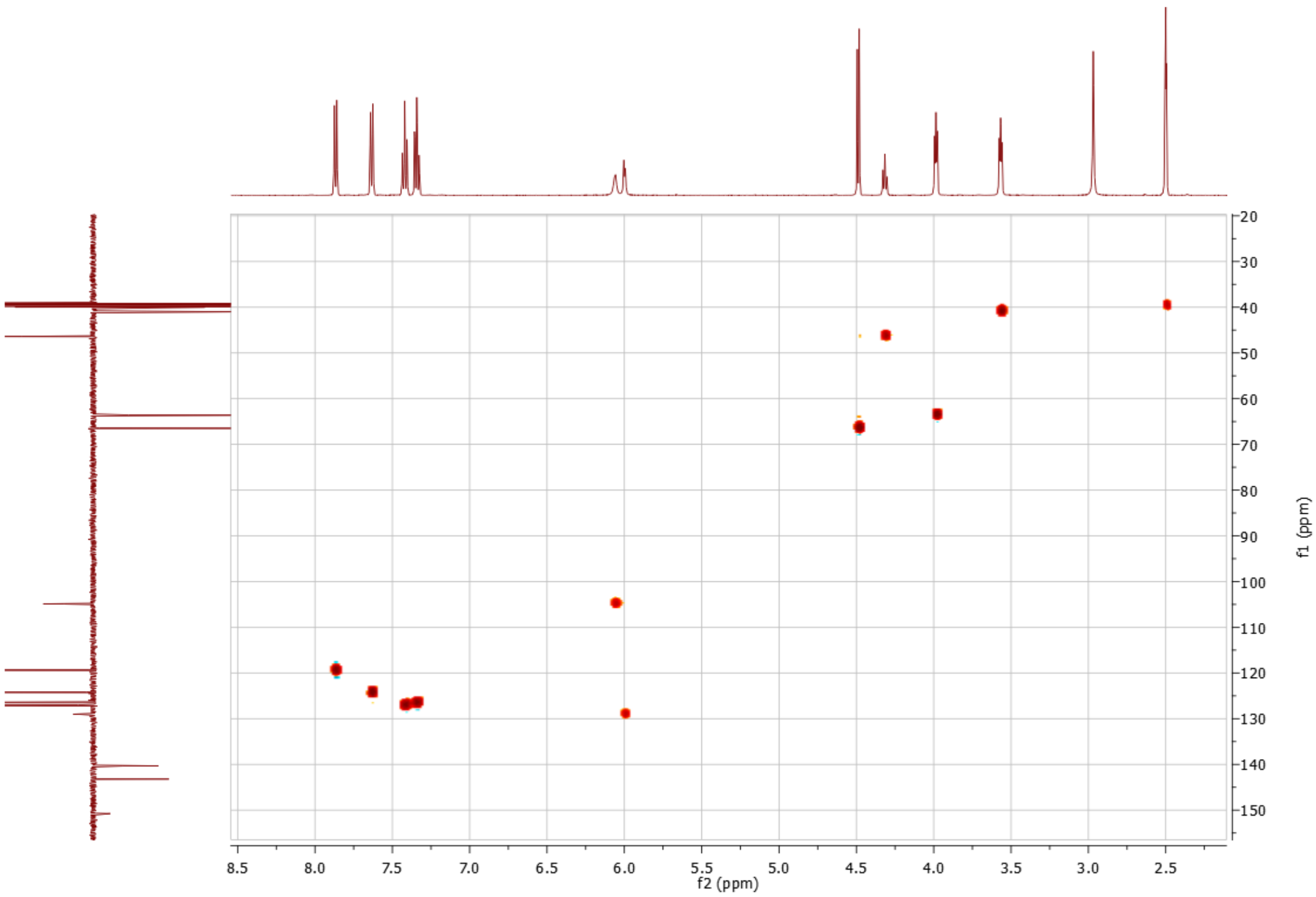

Figure S20: HSQC-NMR (DMSO-d6) spectrum of dihydrooxazine ***21****. Note: To avoid the doubling of the signal of the carbon-carbon double bond, this compound was measured at 100°C.*

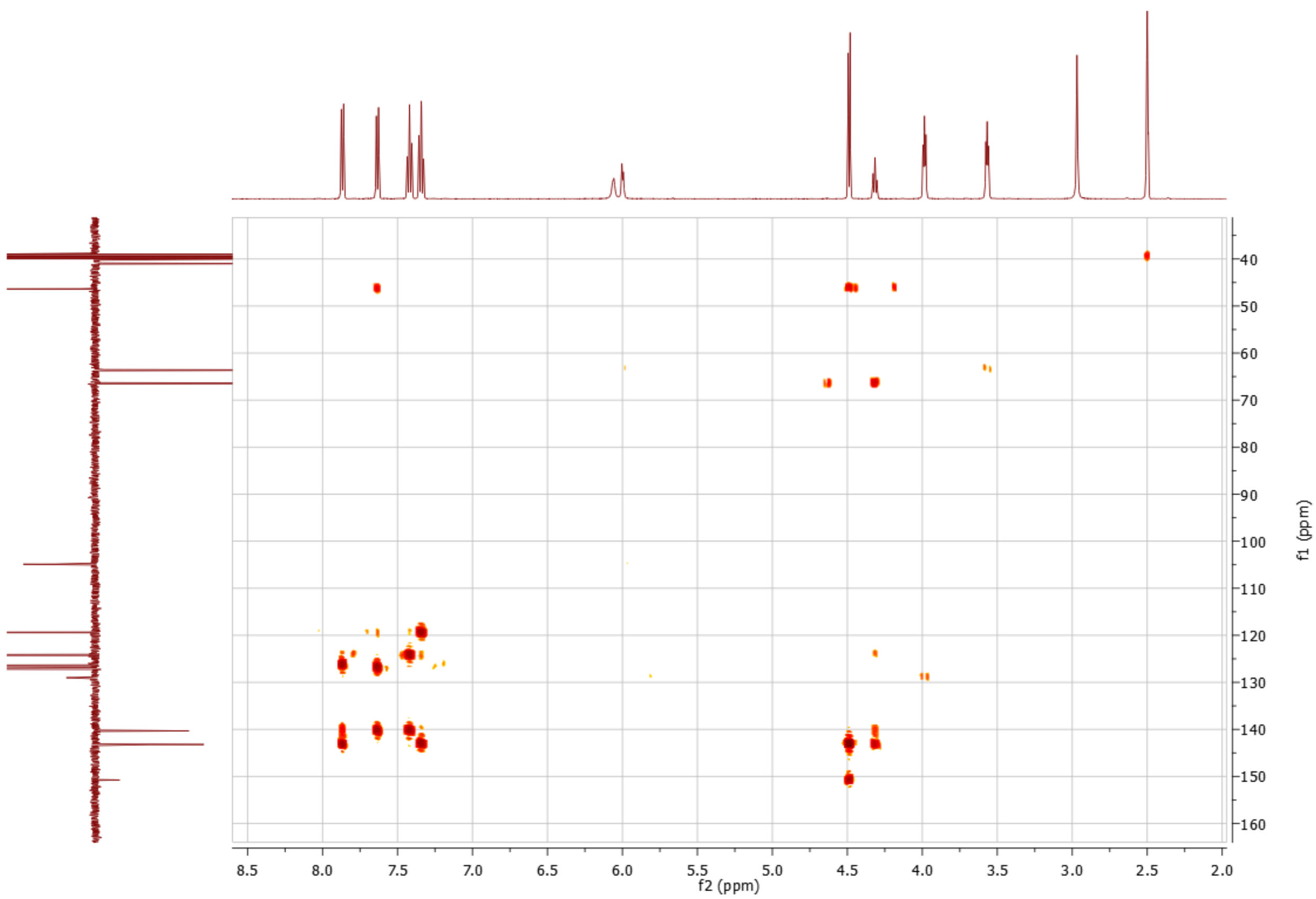

*Figure S21: HMBC-NMR (DMSO-d6) spectrum of dihydrooxazine **21**.Note: To avoid the doubling of the signal of the carbon-carbon double bond, this compound was measured at 100°C.*

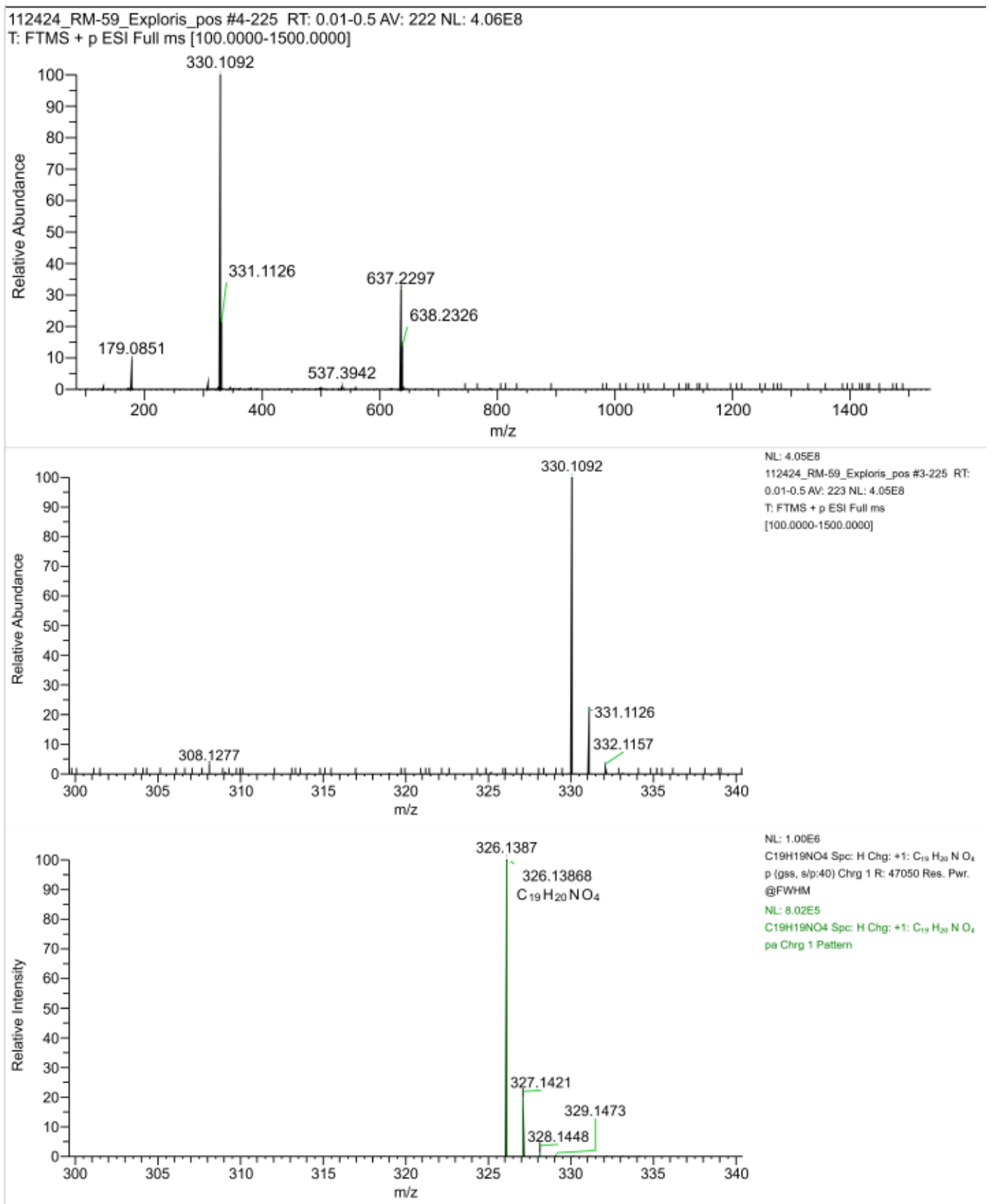

*Figure S22: HRMS spectrum of dihydrooxazine **21**.*

Hydroxylimine 34.

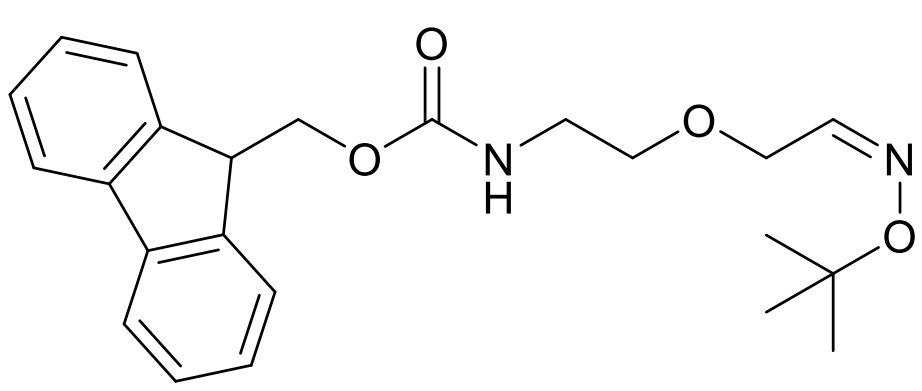

*Figure S23: Structure of hydroxylimine **34**.*

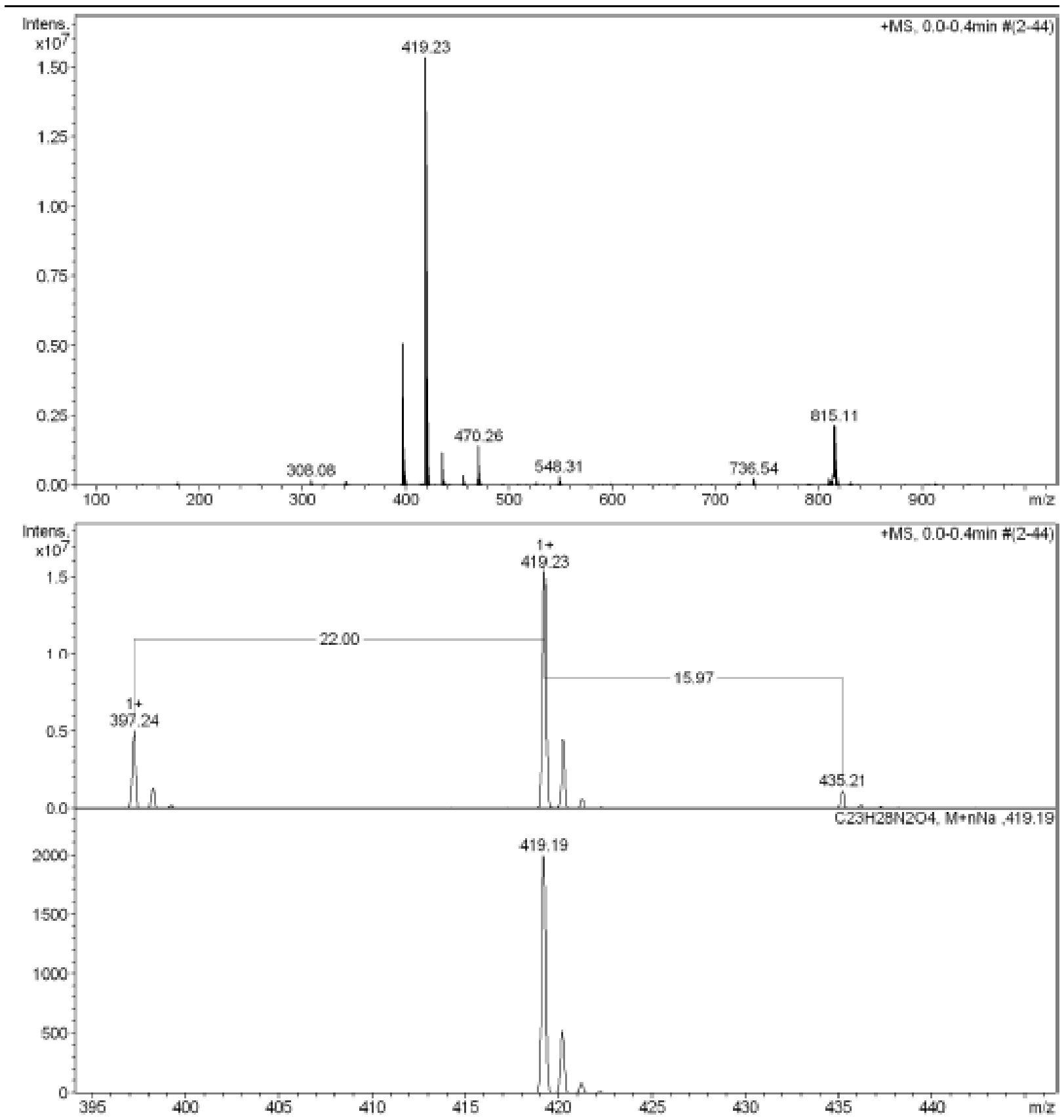

*Figure S24: HRMS spectrum of hydroxylimine **34**.*

Hydroxylamine **18**.

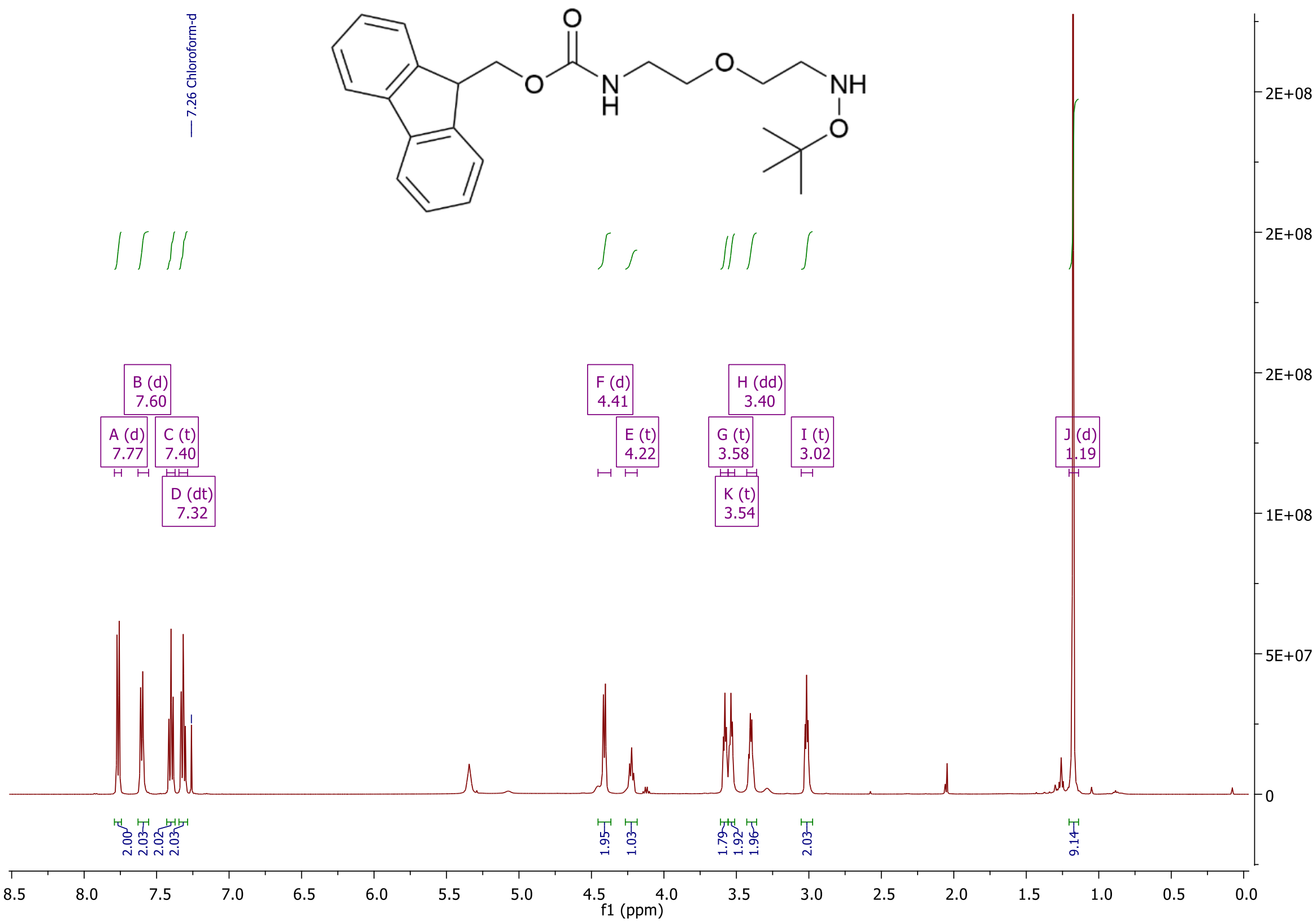

*Figure S25: ^{1}H-NMR (500.1 MHz, Chloroform-d) spectrum of hydroxylamine **18**.*

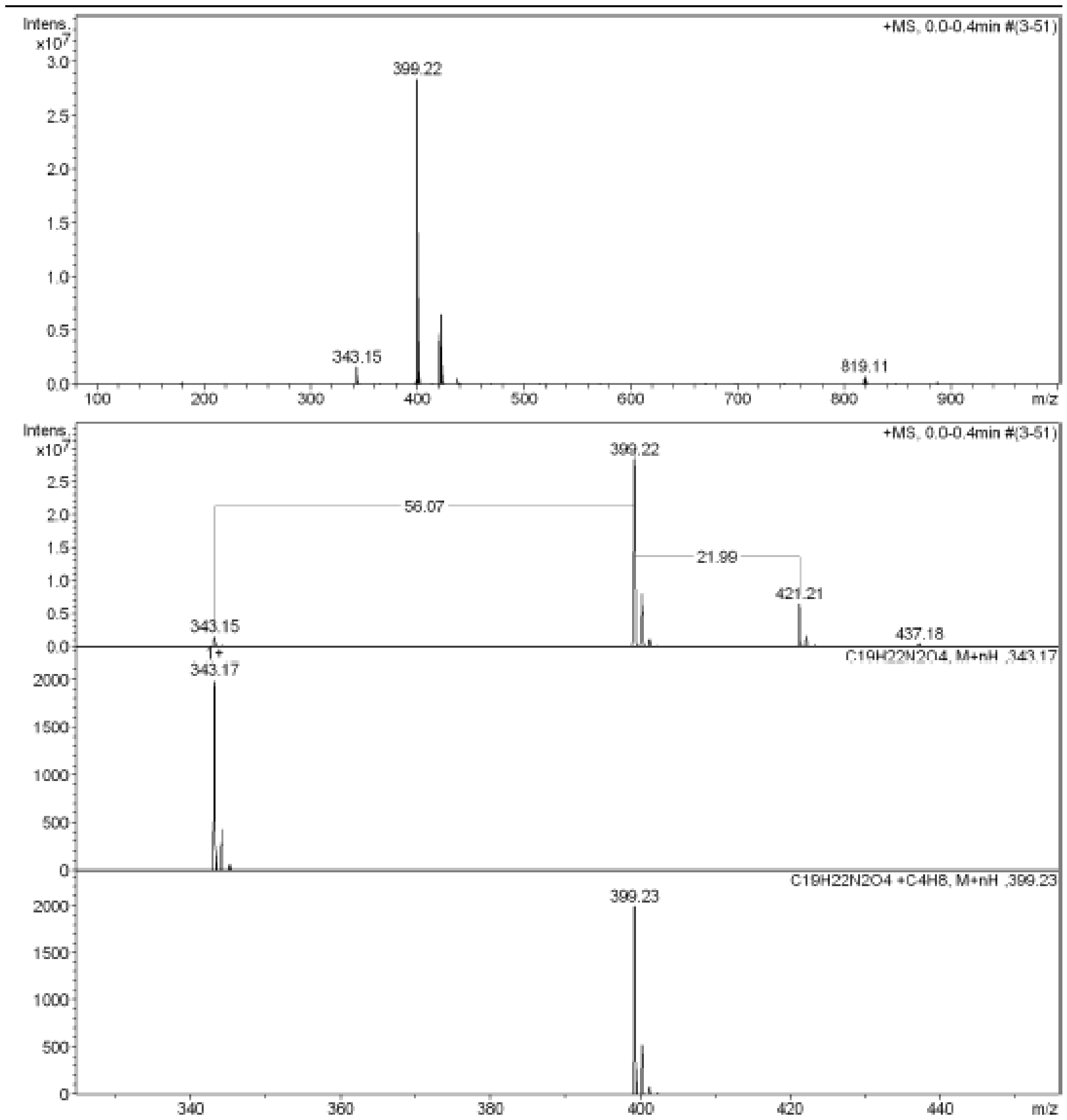

*Figure S26: HRMS spectrum of hydroxylamine **18**.*

Oxo-monomer **20**.

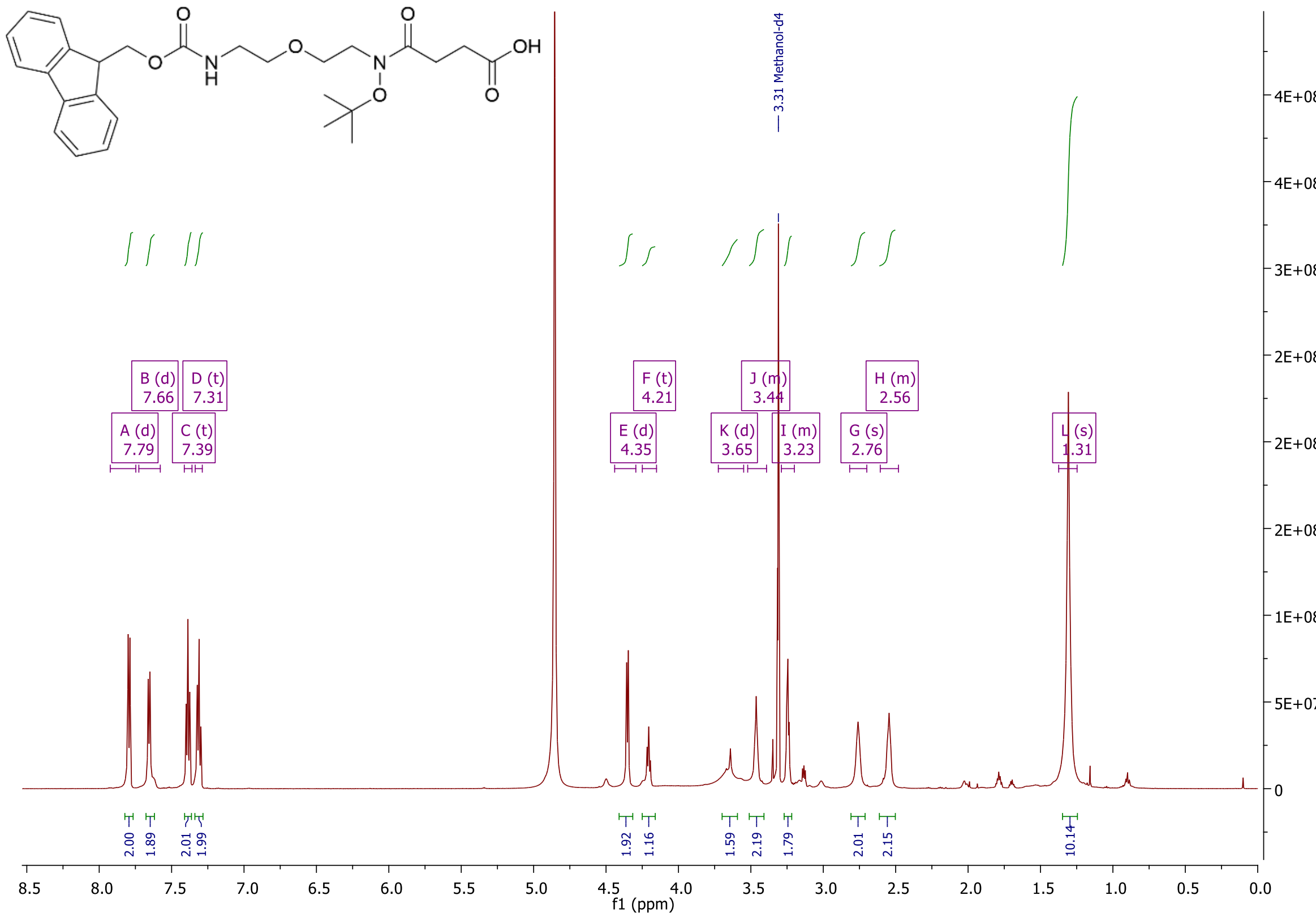

*Figure S27: ^{1}H-NMR (500.1 MHz, Methanol-d4) spectrum of oxo-monomer **20**.*

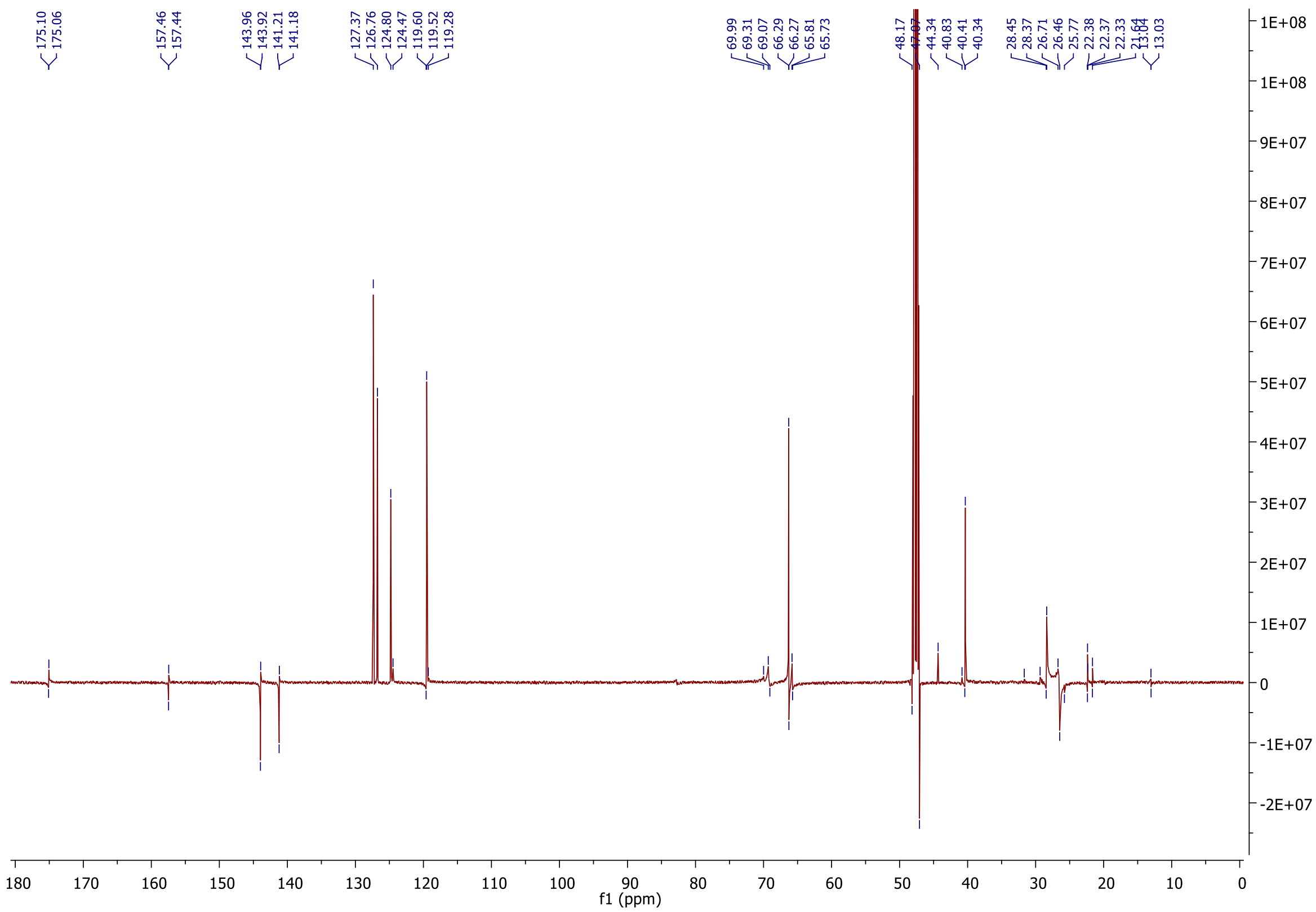

*Figure S28: ^{13}C-NMR (151 MHz, Methanol-d4) spectrum of oxo-monomer **20**.*

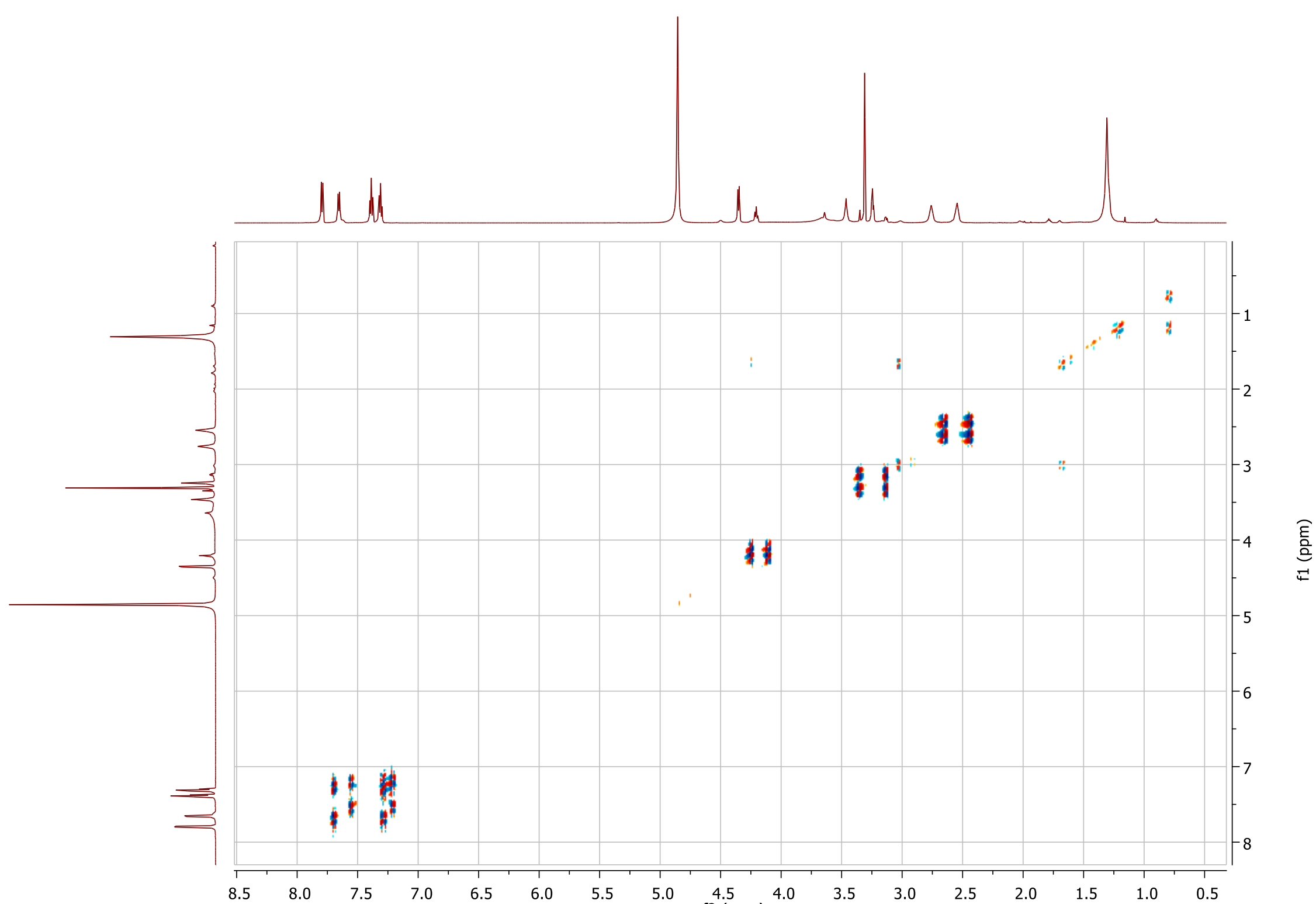

*Figure S29: COSY-NMR (Methanol-d4) spectrum of oxo-monomer **20**.*

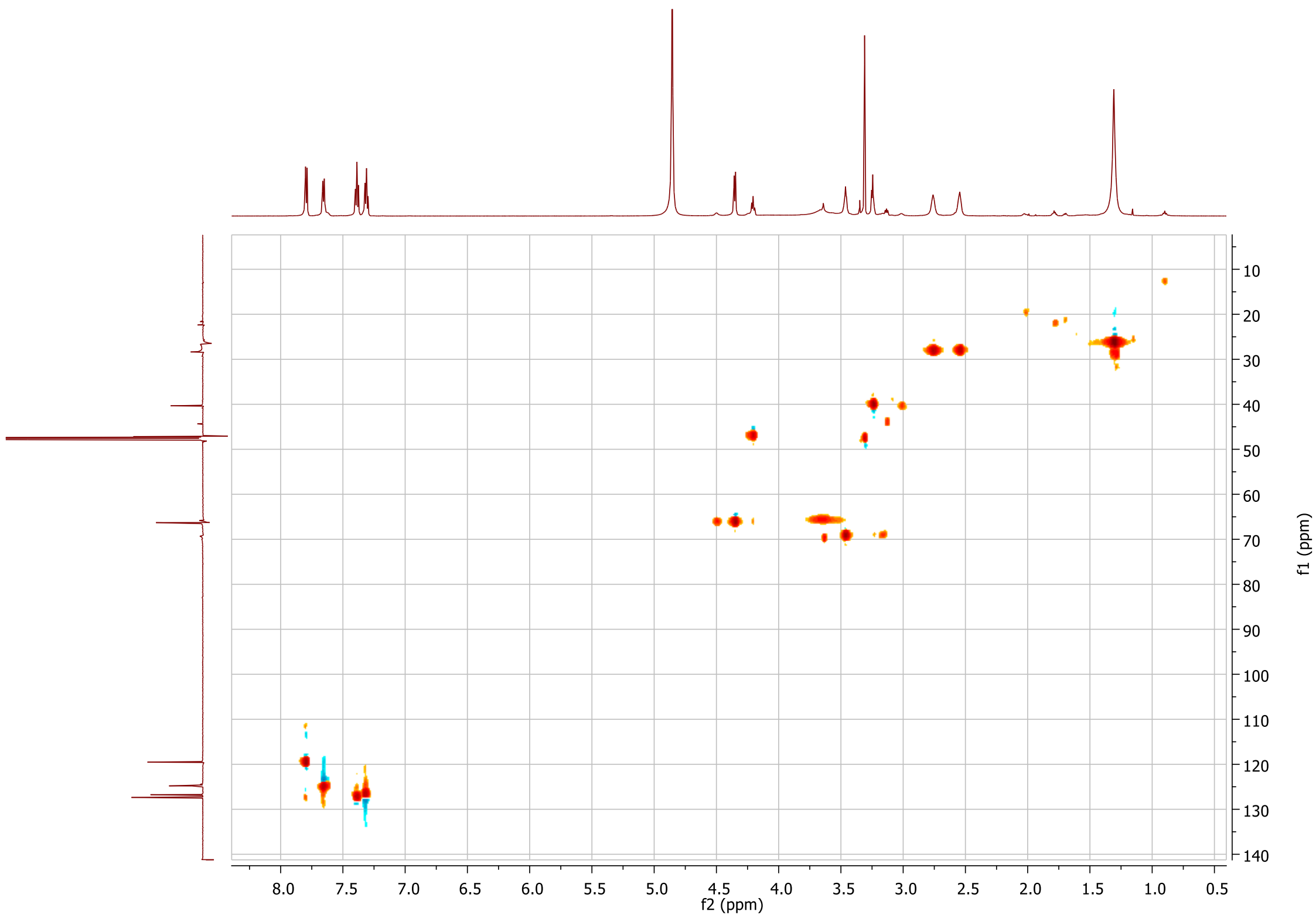

*Figure S30: HSQC-NMR (Methanol-d4) spectrum of oxo-monomer **20**.*

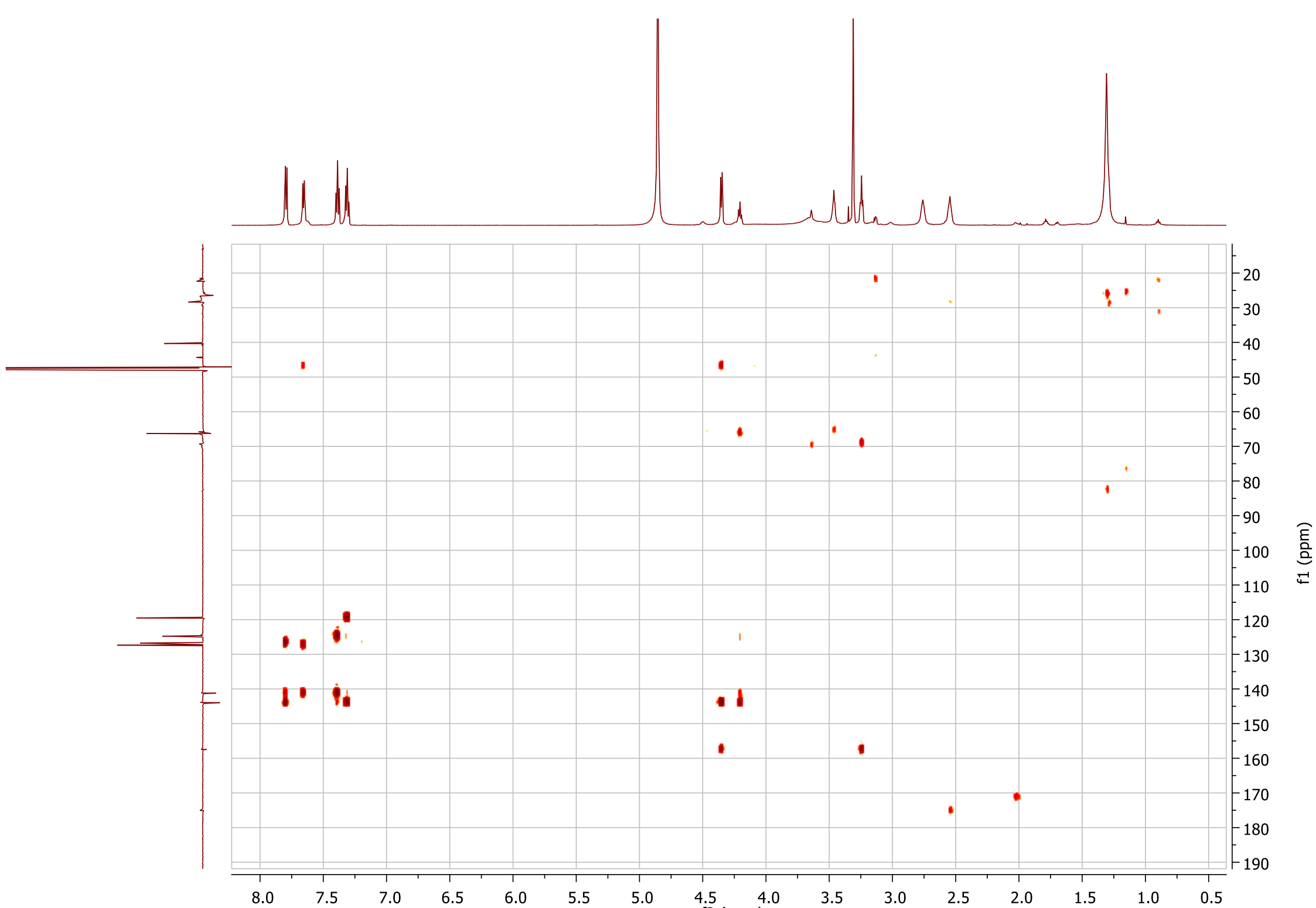

*Figure S31: HMBC-NMR (Methanol-d4) spectrum of oxo-monomer **20**.*

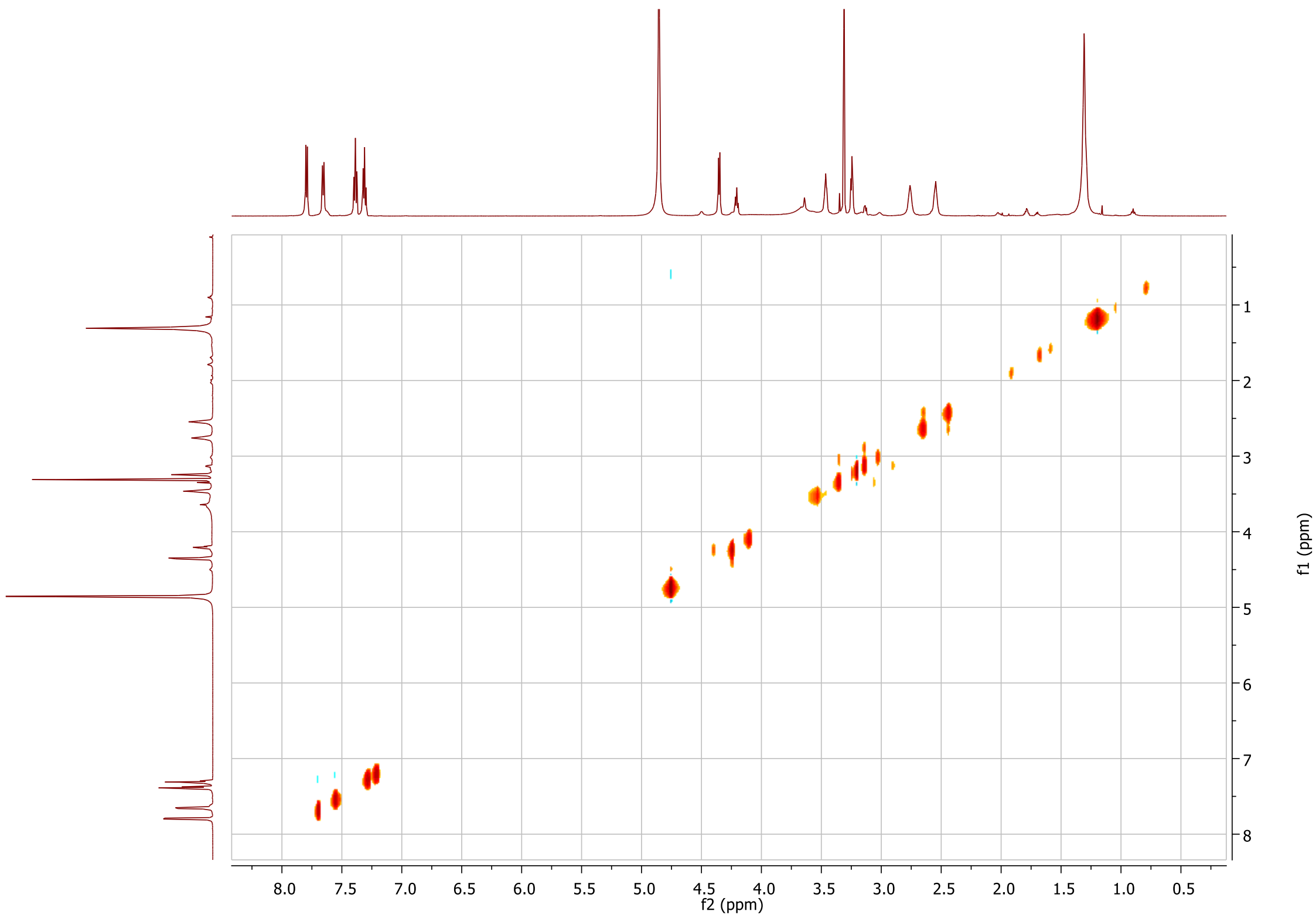

*Figure S32: NOESY-NMR (Methanol-d4) spectrum of oxo-monomer **20**.*

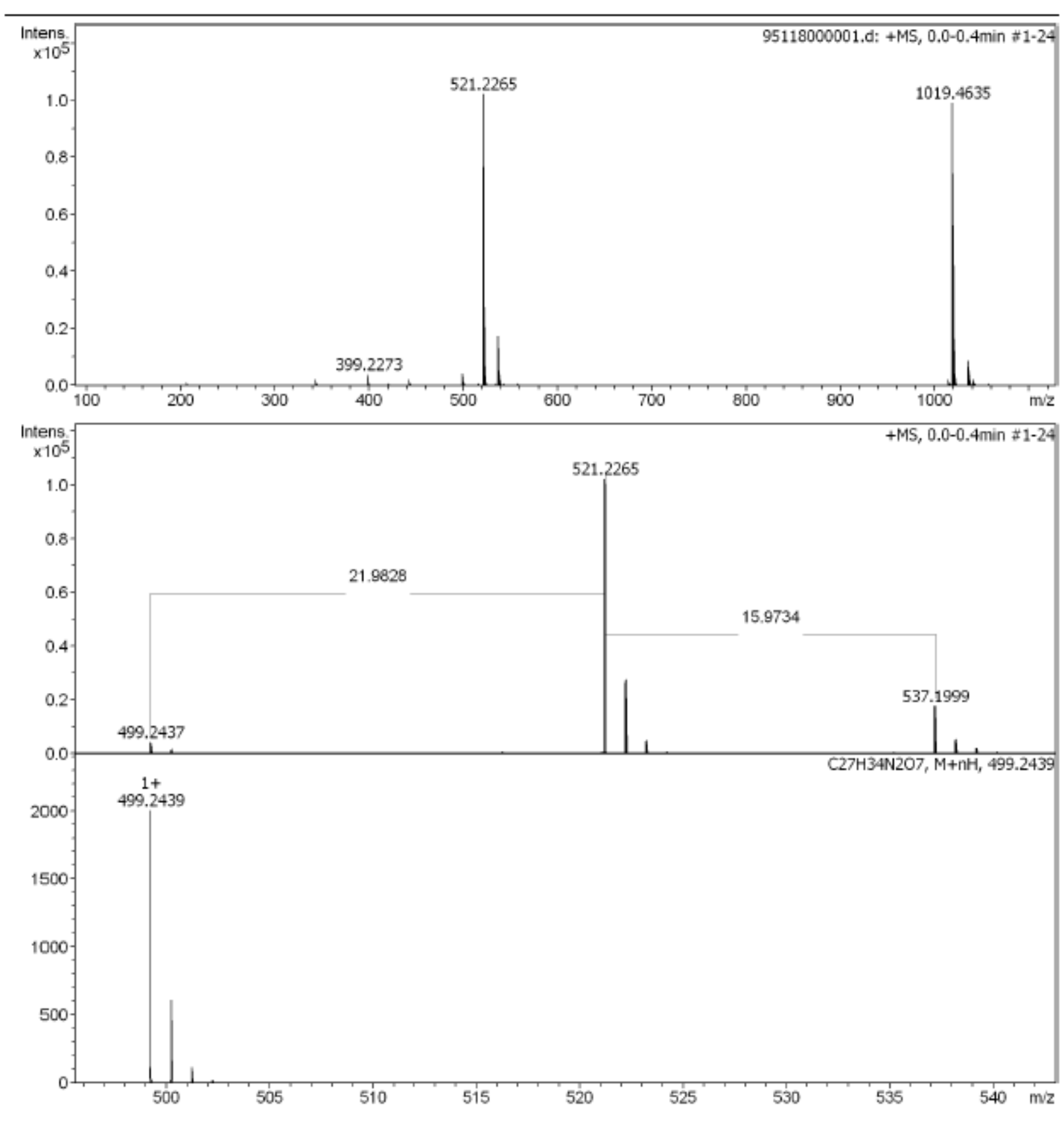

*Figure S33: HRMS spectrum of oxo-monomer **20**.*

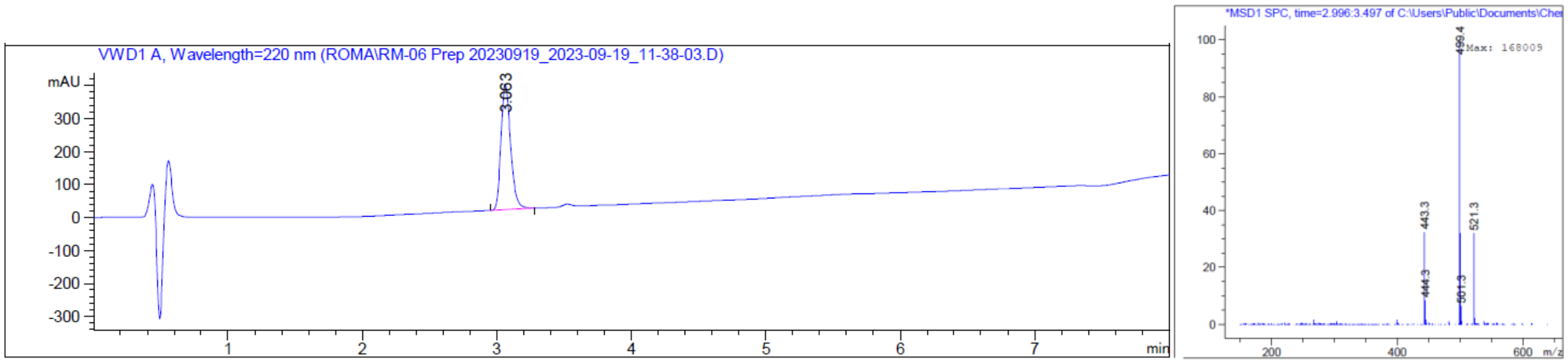

*Figure S34: LC-MS chromatogram of oxo-monomer **20**. (left UV, right MS)*

DFO*^{sp} 3.

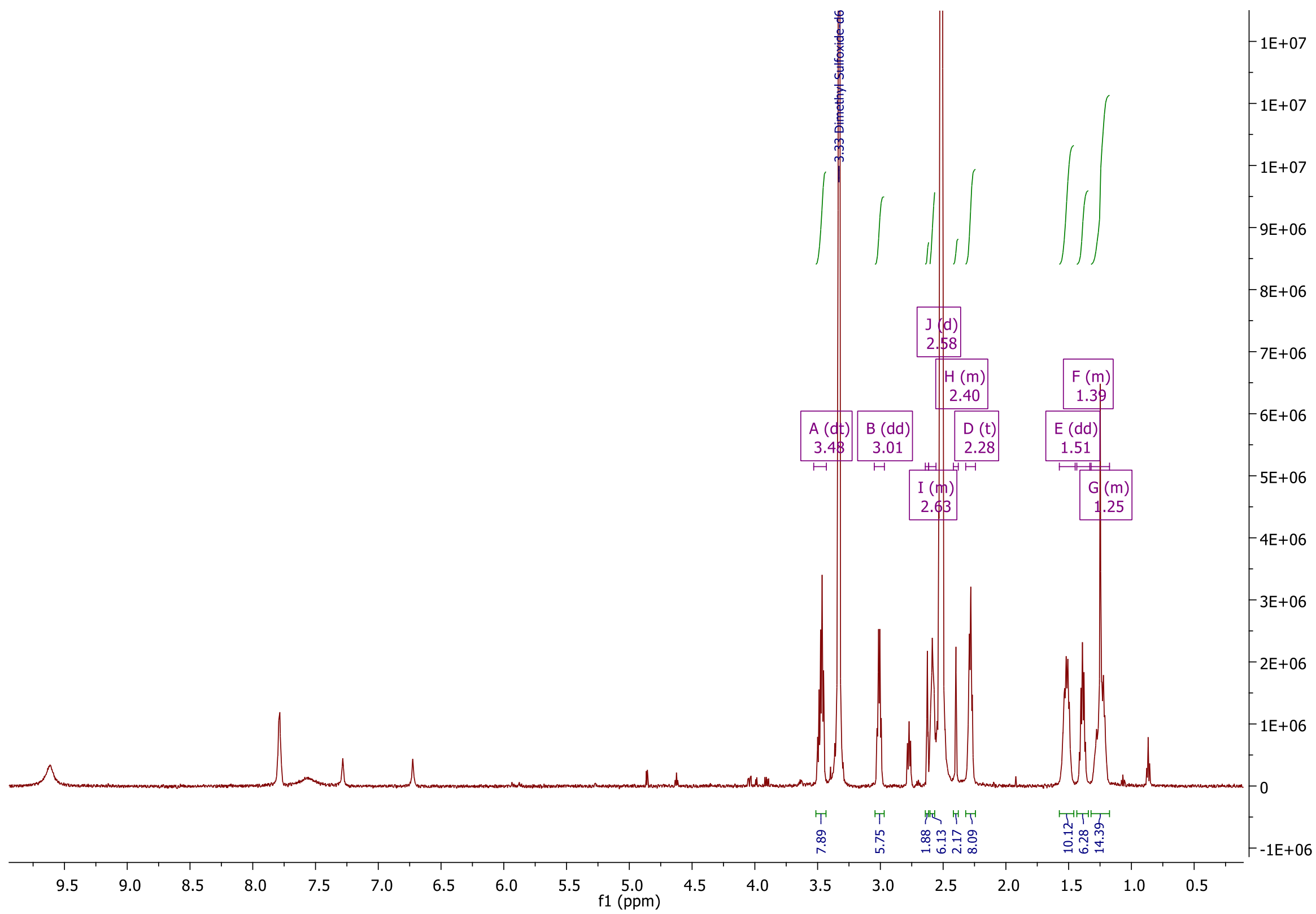

Figure S35: ^{1}H-NMR (600 MHz, DMSO-d6) spectrum of DFO^{sp} **3**.*

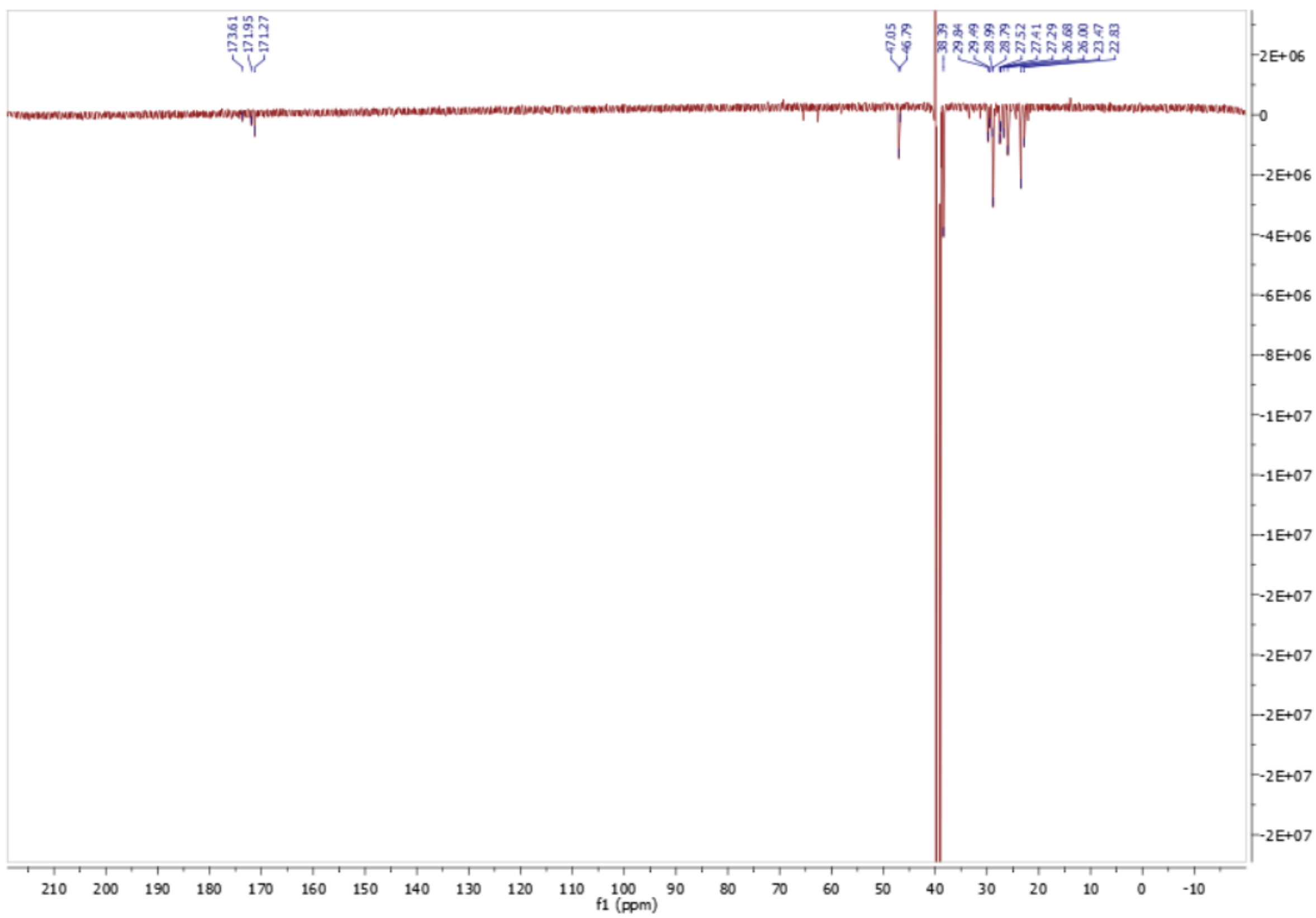

Figure S36: ^{13}C-NMR (151 MHz, DMSO-d6) of DFO^{sp} **3**.*

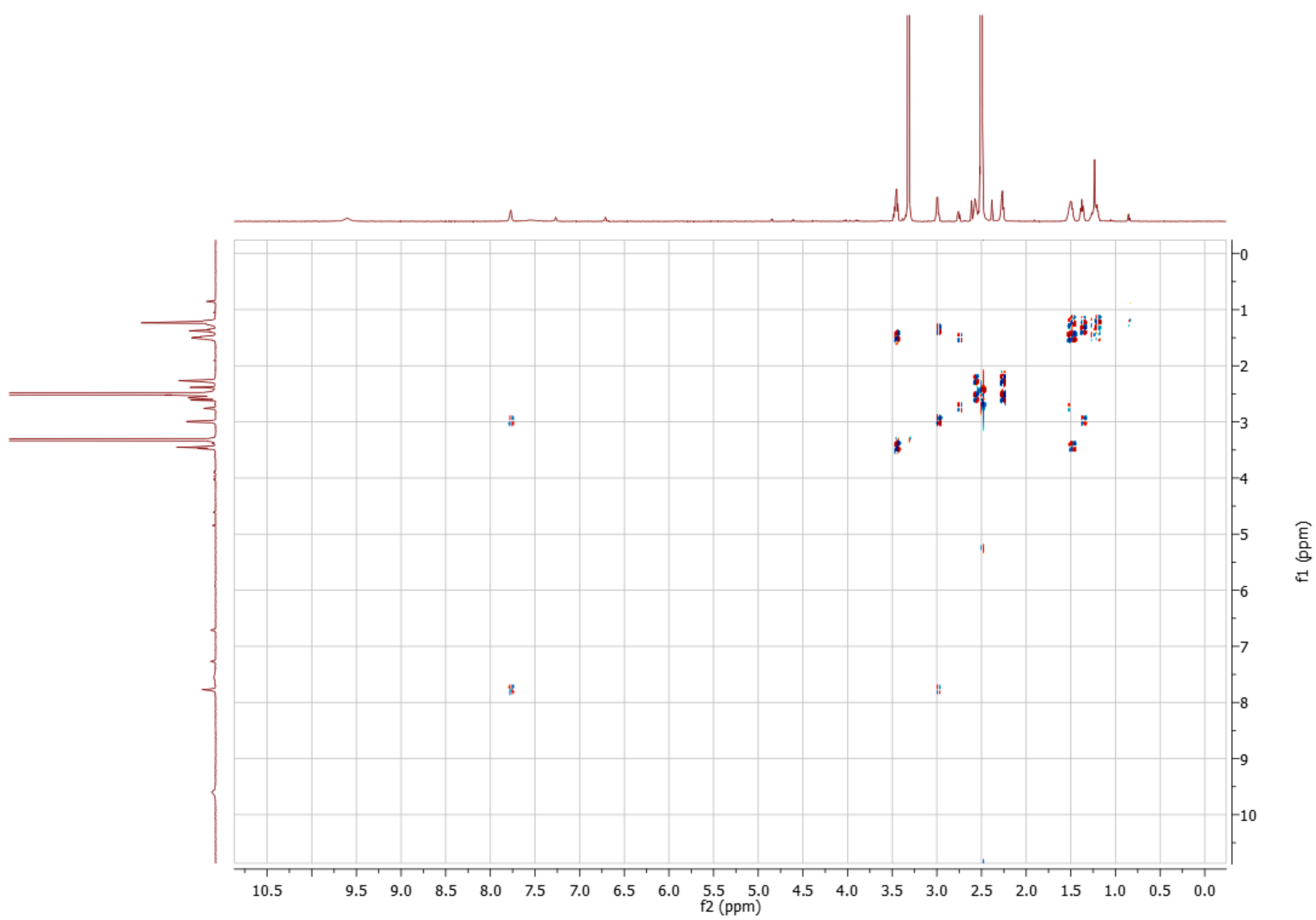

Figure S37: COSY-NMR (DMSO-d6)spectrum of DFO^{sp} **3**.*

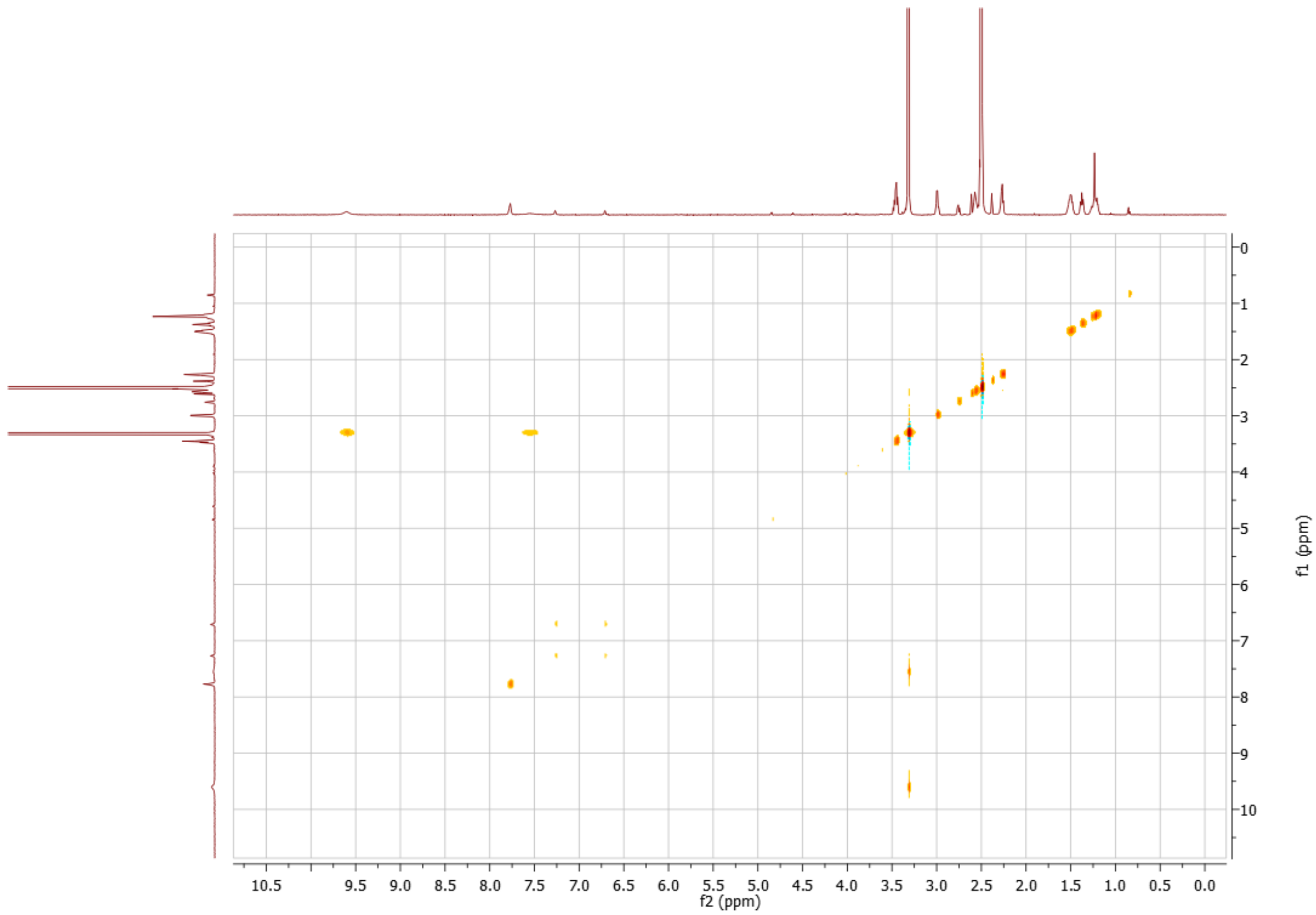

*Figure S38: NOESY-NMR (DMSO-d6) spectrum of DFO*sp **3**.*

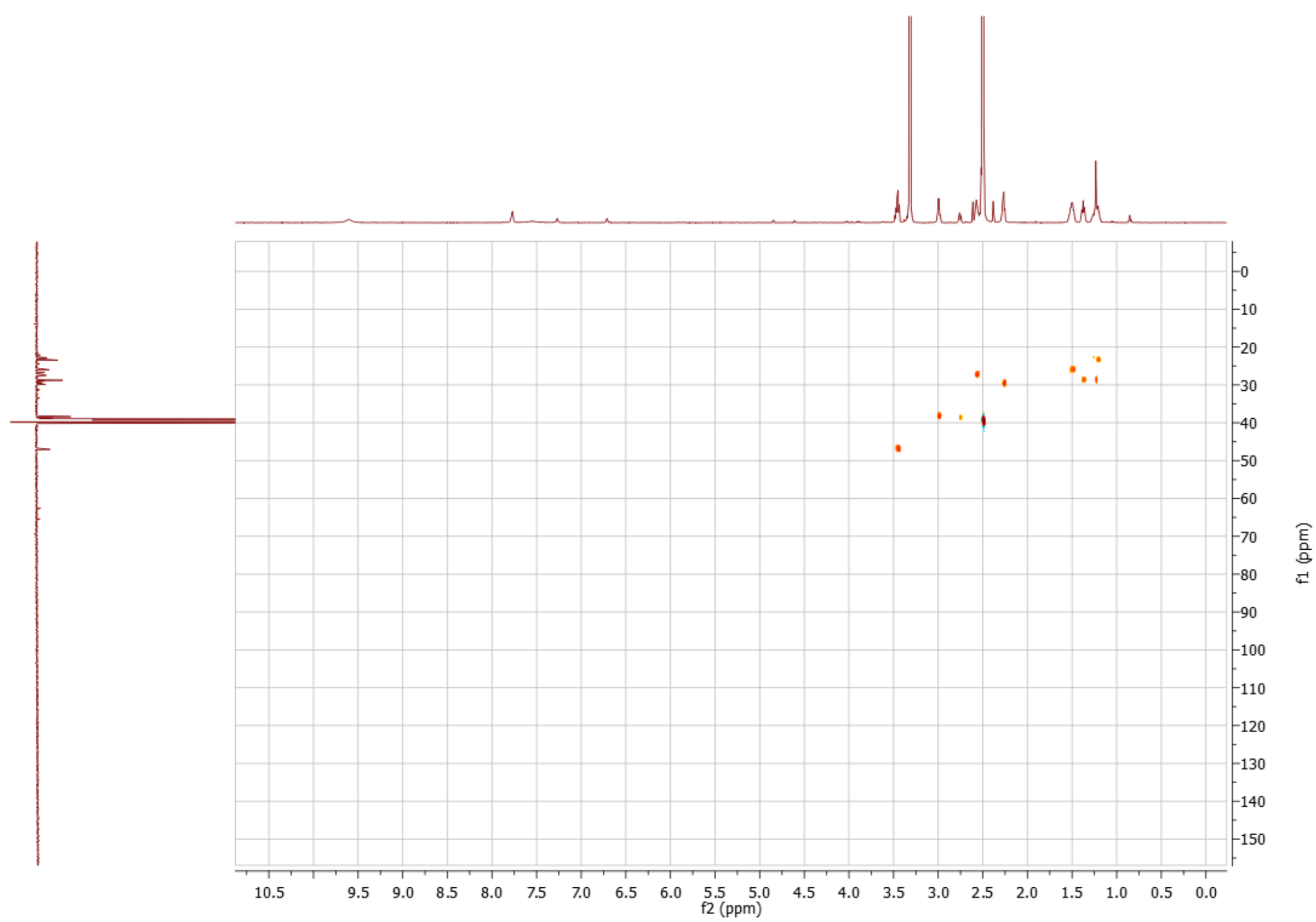

*Figure S39: HSQC-NMR (DMSO-d6) spectrum of DFO*sp **3**.*

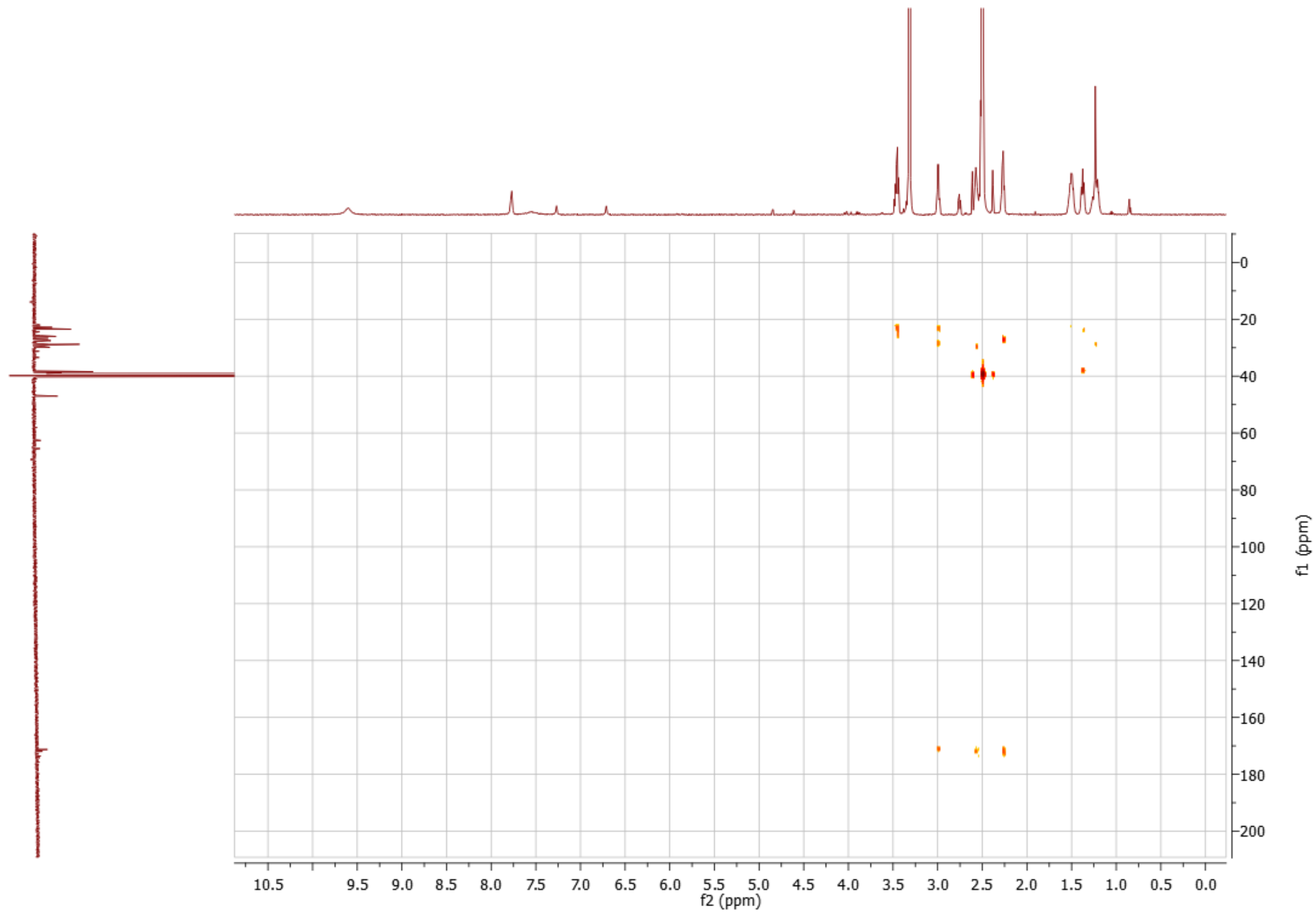

Figure S40: HMBC-NMR (DMSO-d6) spectrum of DFO^{sp} **3**.*

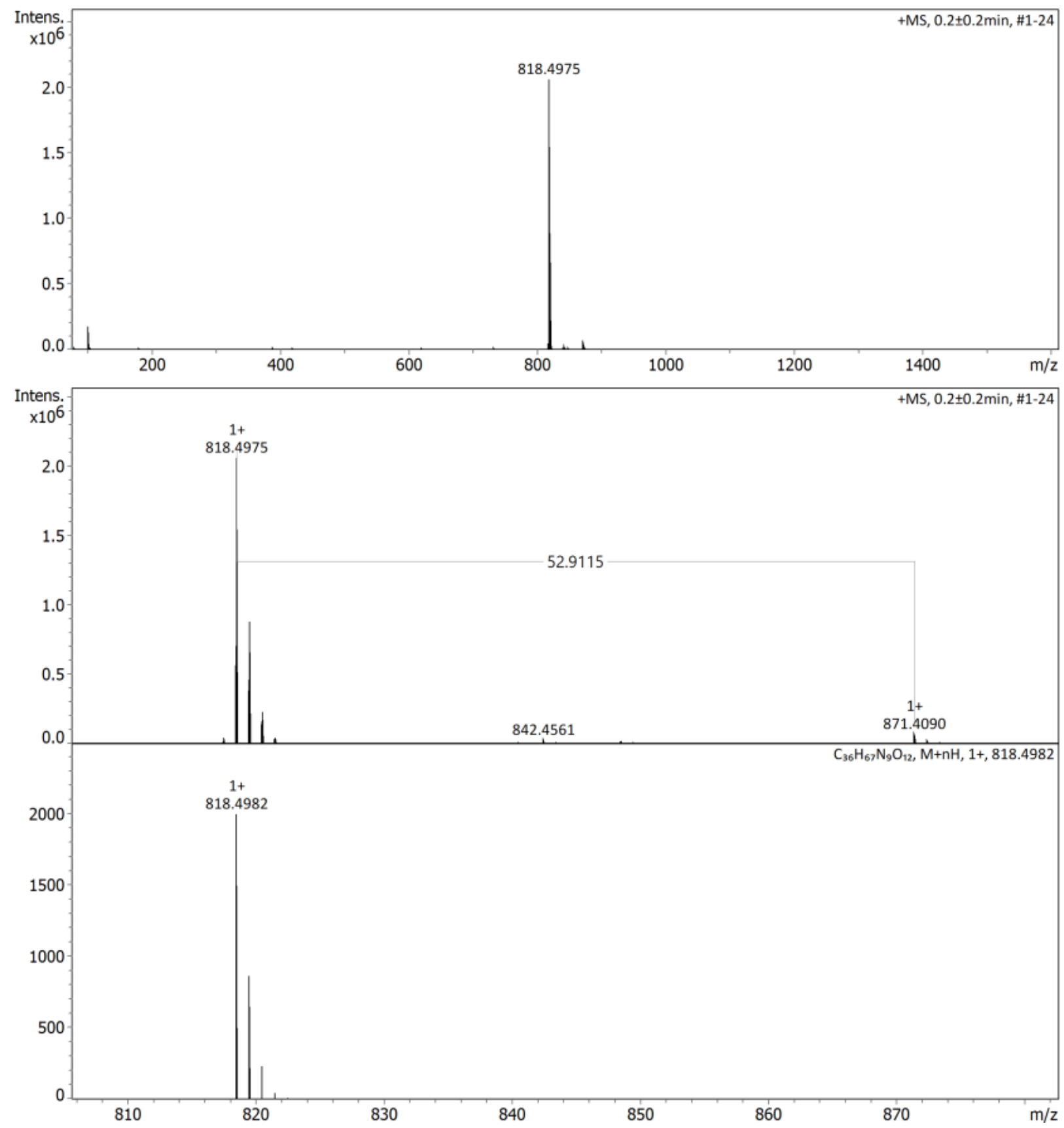

Figure S41: HRMS spectrum of DFO^sp^ **3**.*

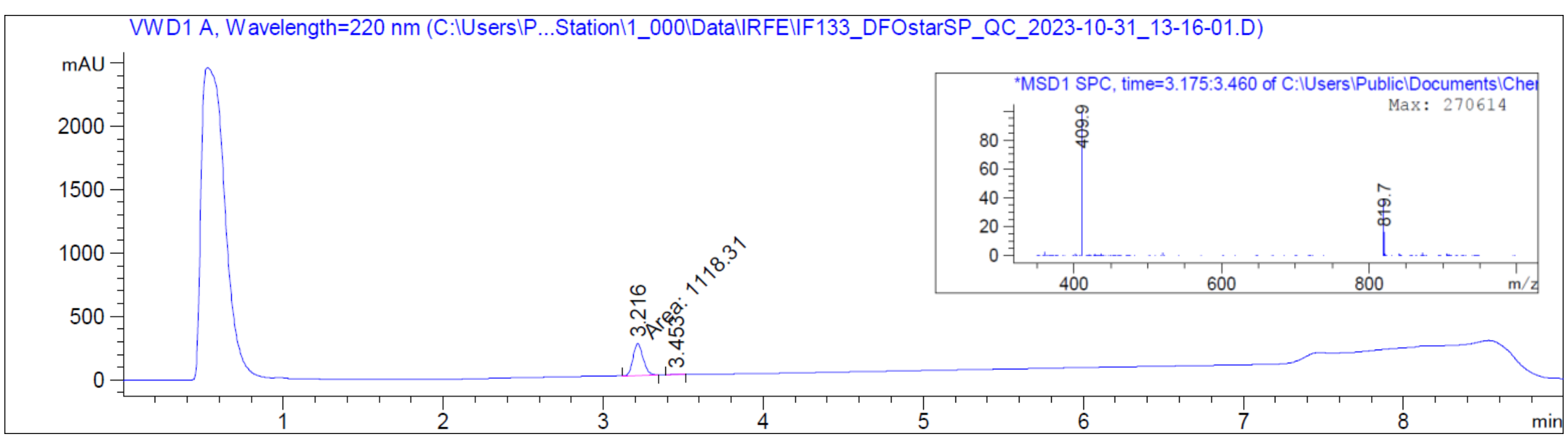

Figure S42: LC-MS analysis of DFO^sp^ **3**.*

^{nat}Ga-DFO **24**.

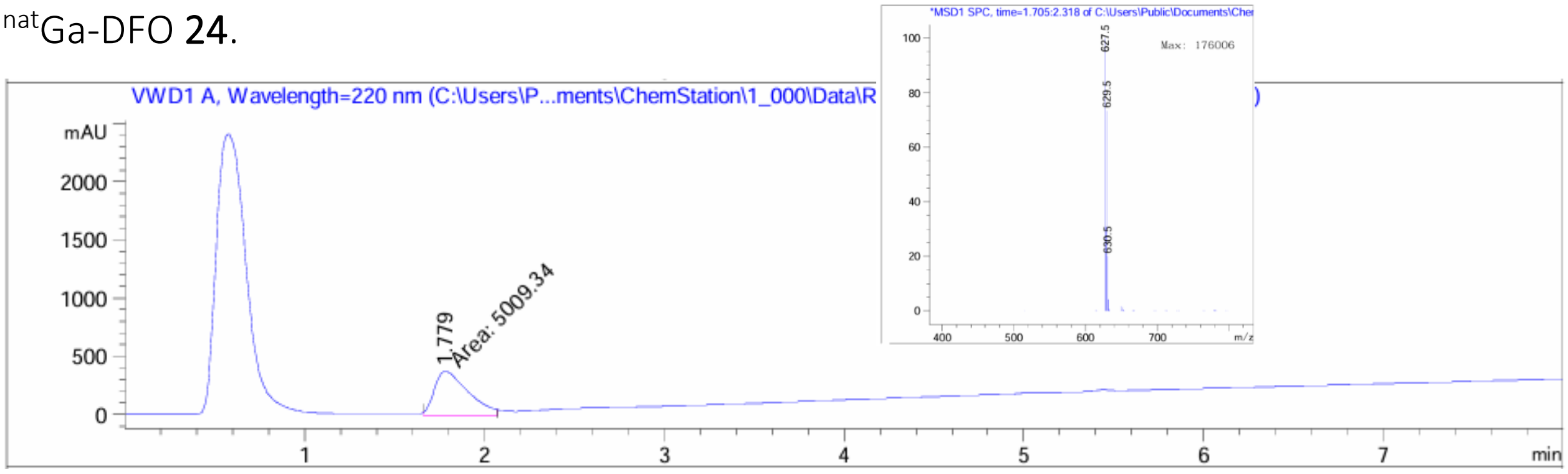

Figure S43: LC-MS analysis of ^{nat}Ga-DFO 24.

^{nat}Ga-DFO* **25**.

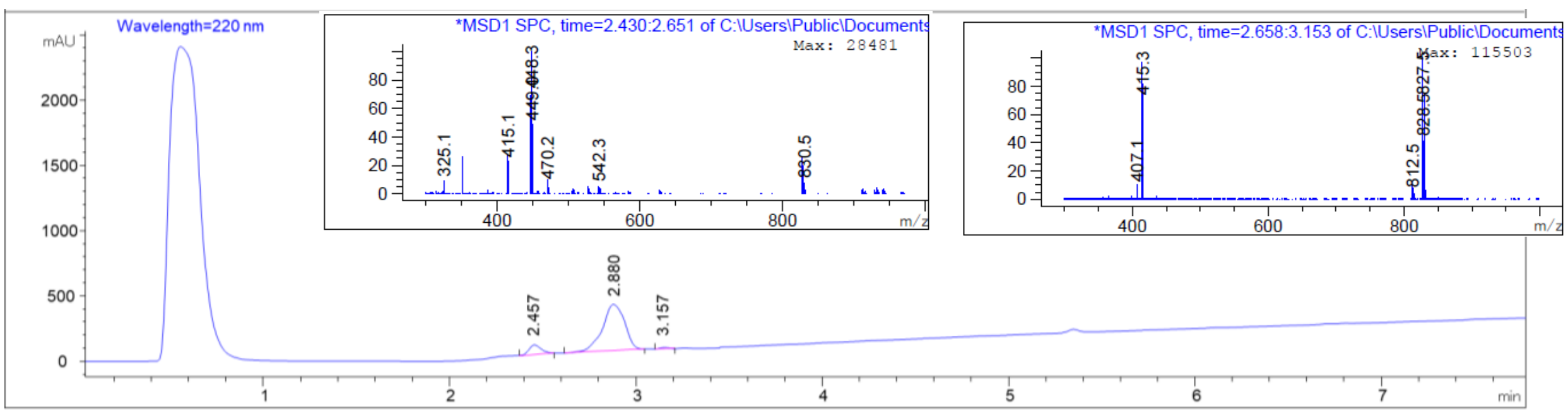

Figure S44: LC-MS analysis of ^{nat}Ga-DFO 25.*

Note: The signal with R_t = 2.457 min seems to be the chelator, complexing two Gallium atoms (~10%). LR-MS spectra; left: : LC-LRMS m/z found (calculated for $C_{34}H_{60}N_8O_{11}Ga_2$): $[M+H^+]^{2+}$ = 448.3 (447.1); right: LC-LRMS m/z found (calculated for $C_{34}H_{60}N_8O_{11}Ga$): $[M+H^+]^+$ = 827.5 (825.4).

^{nat}Ga-DFO*^{sp} **26**.

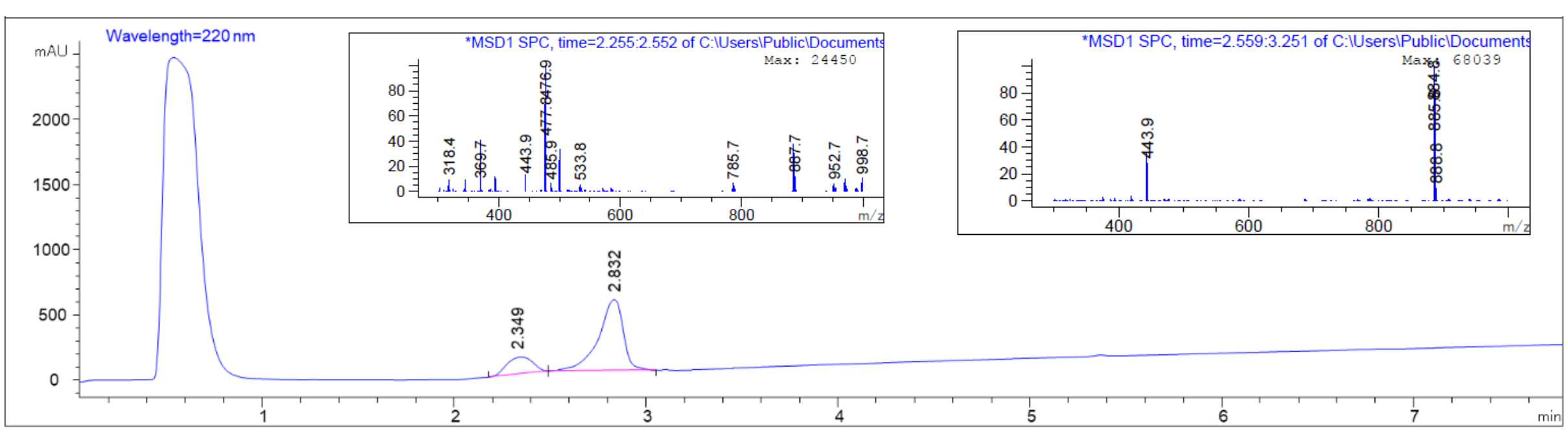

Figure S45: LC-MS analysis of ^{nat}Ga-DFO^{sp} **26**.*

Note: The signal with R_t = 2.349 min seems to be the chelator complexing two Gallium atoms (~20%). LC-LRMS spectra; left: MS m/z found (calculated for $C_{36}H_{63}N_9O_{12}Ga_2$): $[M+H^+]^+$ = 952.7 (951.3); right: MS m/z found (calculated for $C_{36}H_{63}N_9O_{12}Ga$): $[M+H^+]^+$ = 884.8 (882.4)

^{nat}Ga-oxoDFO* **27**.

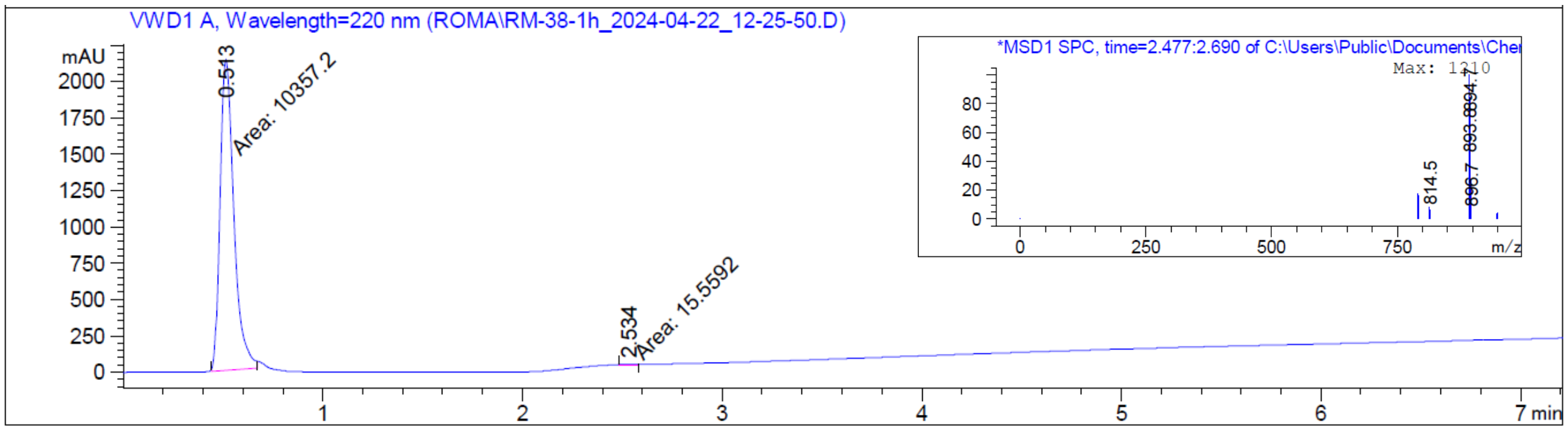

Figure S46: LC-MS analysis of ^{nat}Ga-oxoDFO **27**.*

^{nat}Zr-DFO* **29**.

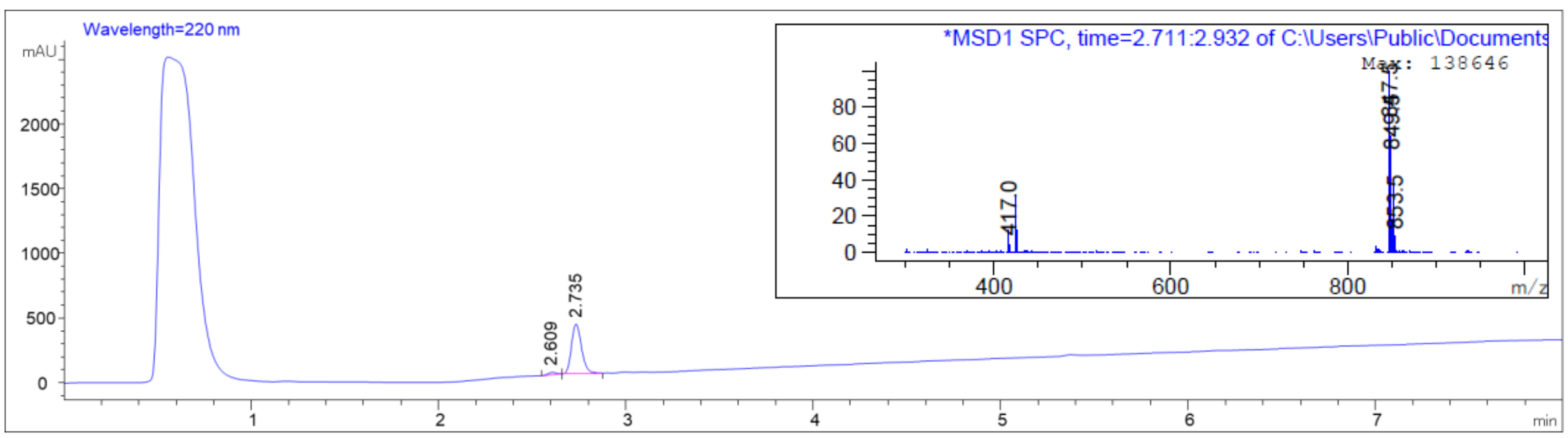

Figure S47: LC-MS analysis of ^{nat}Zr-DFO **28**.*

^{nat}Zr-DFO*^{sp} **30**.

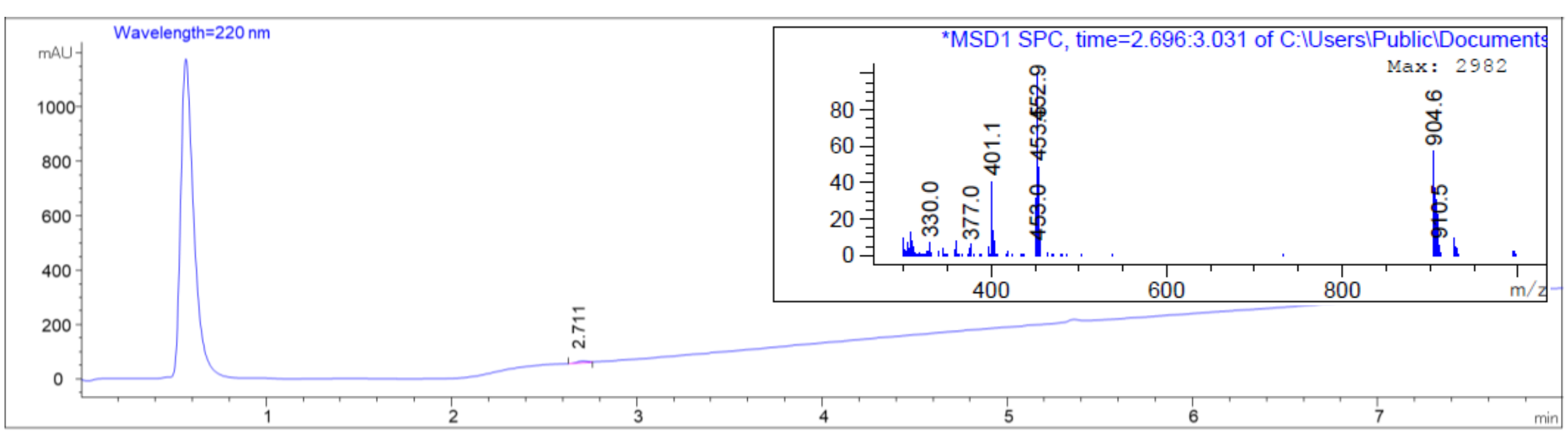

Figure S48: LC-MS analysis of ^{nat}Zr-DFO^{sp} **29**.*

^{nat}Zr-oxoDFO* **31**.

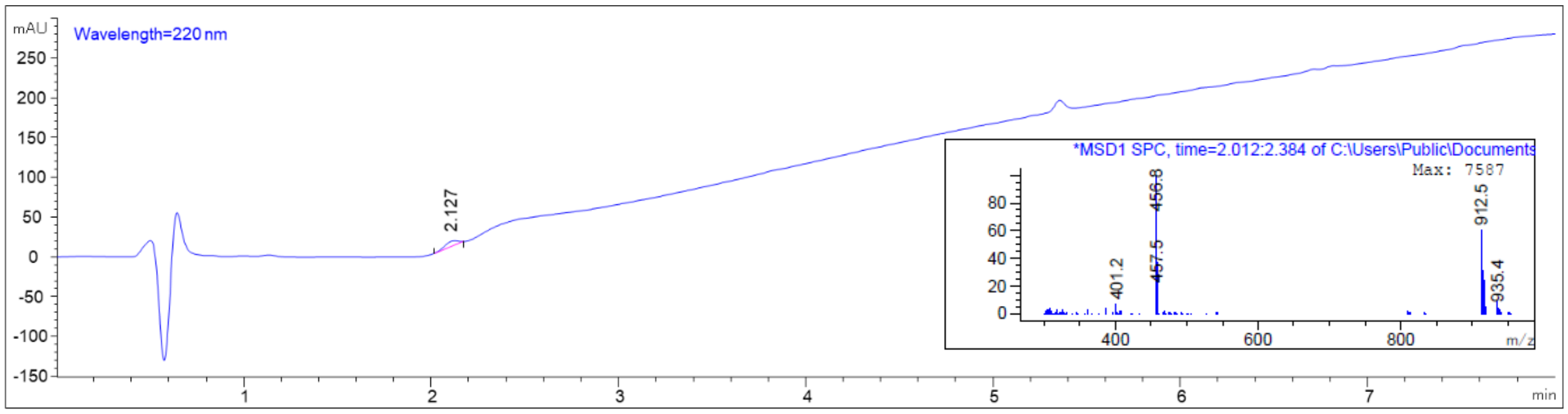

Figure S49: LC-MS analysis of ^{nat}Zr-oxoDFO **30**.*

In vitro characterization of [^{68}Ga]Ga- and [^{89}Zr] Zr-DFO-analogs **5**, **6**, **7**, **8**, **9**, **10**, **11**, and **12**.

*Table S1: In vitro characterization of [^{68}Ga]Ga-DFO-analogs **5**, **6**, **7**, and **8**.*

Radiolabeled compound	Log P (n=6)	Incubation time (h)	Protein binding in human serum (%) (n=3)	Protein binding in mouse serum (%) (n=3)	Stability in human serum (%) (n=3)	Stability in PBS (%) (n=3)
[^{68}Ga]Ga-DFO **5**	− 3.3 ± 0.2	0.5	26.1 ± 1.1	1.4 ± 0.5	98.3 ± 0.8	99.5 ± 0.2
		1.0	28.0 ± 2.8	1.7 ± 0.6	98.9 ± 0.3	99.5 ± 0.1
		2.0	26.7 ± 3.9	1.7 ± 0.3	98.4 ± 0.5	98.0 ± 1.2
[^{68}Ga]Ga-DFO* **6**	-2.4 ± 0.3	0.5	25.7 ± 1.9	7.8 ± 1.9	97.1 ± 2.4	99.4 ± 0.6
		1.0	29.7 ± 0.7	4.7 ± 1.1	99.4 ± 0.3	99.3 ± 0.5
		2.0	30.2 ± 2.5	4.3 ± 0.1	98.6 ± 1.1	98.5 ± 1.0
[^{68}Ga]Ga-DFO*^{sp} **7**	-3.2 ± 0.1	0.5	3.0 ± 0.8	6.0 ± 1.2	90.7 ± 4.3	93.9 ± 2.2
		1.0	4.1 ± 0.7	4.8 ± 0.5	92.7 ± 3.1	96.0 ± 0.9
		2.0	4.4 ± 1.2	4.3 ± 0.9	88.2 ± 5.6	94.8 ± 1.8
[^{68}Ga]Ga-oxoDFO* **8**	-3.4 ±0.3	0.5	4.9 ± 0.6	3.5 ± 1.0	93.3 ± 5.1	99.4 ± 0.1
		1.0	5.4 ± 0.3	3.4 ± 0.6	96.5 ± 1.0	97.6 ± 0.5
		2.0	9.6 ± 1.1	6.5 ± 1.1	96.4 ± 0.7	96.9 ± 2.3

*Table S2: In vitro characterization of [^{89}Zr]Zr-DFO-analogs **9**, **10**, **11**, and **12**.*

Radiolabeled compound	Log P (n=6)	Incubation time (h)	Protein binding in human serum (%) (n=3)	Protein binding in mouse serum (%) (n=3)	Stability in human serum (%) (n=3)	Stability in PBS (%) (n=3)

[89]Zr]Zr-DFO **9**	-2.7 ± 0.1	2	6.3 ± 2.8	13.7 ± 6.6	93.5 ± 8.4	99.9 ± 0.1
		24	4.4 ± 1.4	8.2 ± 3.2	95.4 ± 6.4	99.8 ± 0.1
		48	3.4 ± 1.1	13.0 ± 1.6	93.4 ±7.4	97.8 ± 1.5
[89]Zr]Zr-DFO* **10**	-2.6 ± 0.02	2	4.3 ± 1.2	14.2 ± 2.8	95.4±0.6	99.3 ± 0.6
		24	4.0 ± 0.8	6.0 ± 1.2	95.7±0.4	96.3 ± 1.5
		48	4.1 ± 1.2	5.8 ± 1.1	98.1±1.8	97.8 ± 0.9
[89]Zr]Zr-DFO*sp **11**	-2.4 ± 0.3	2	6.3 ± 3.6	13.2 ± 1.4	95.1±1.5	96.7 ± 0.6
		24	3.8 ± 0.9	4.0 ± 1.0	98.0±0.2	97.2 ± 0.3
		48	7.1 ±5.0	5.5 ± 1.5	90.4±8.3	97.6 ± 0.3
[89]Zr]Zr-oxoDFO* **12**	-2.9 ± 0.2	2	10.9 ± 5.7	17.1 ± 6.1	95.5 ± 4.5	99.7 ± 0.2
		24	4.8 ± 0.4	4.7 ± 0.6	98.5 ± 1.8	99.8 ± 0.1
		48	6.3 ±1.7	2.3 ± 0.5	96.4 ± 2.9	99.8 ± 0.1

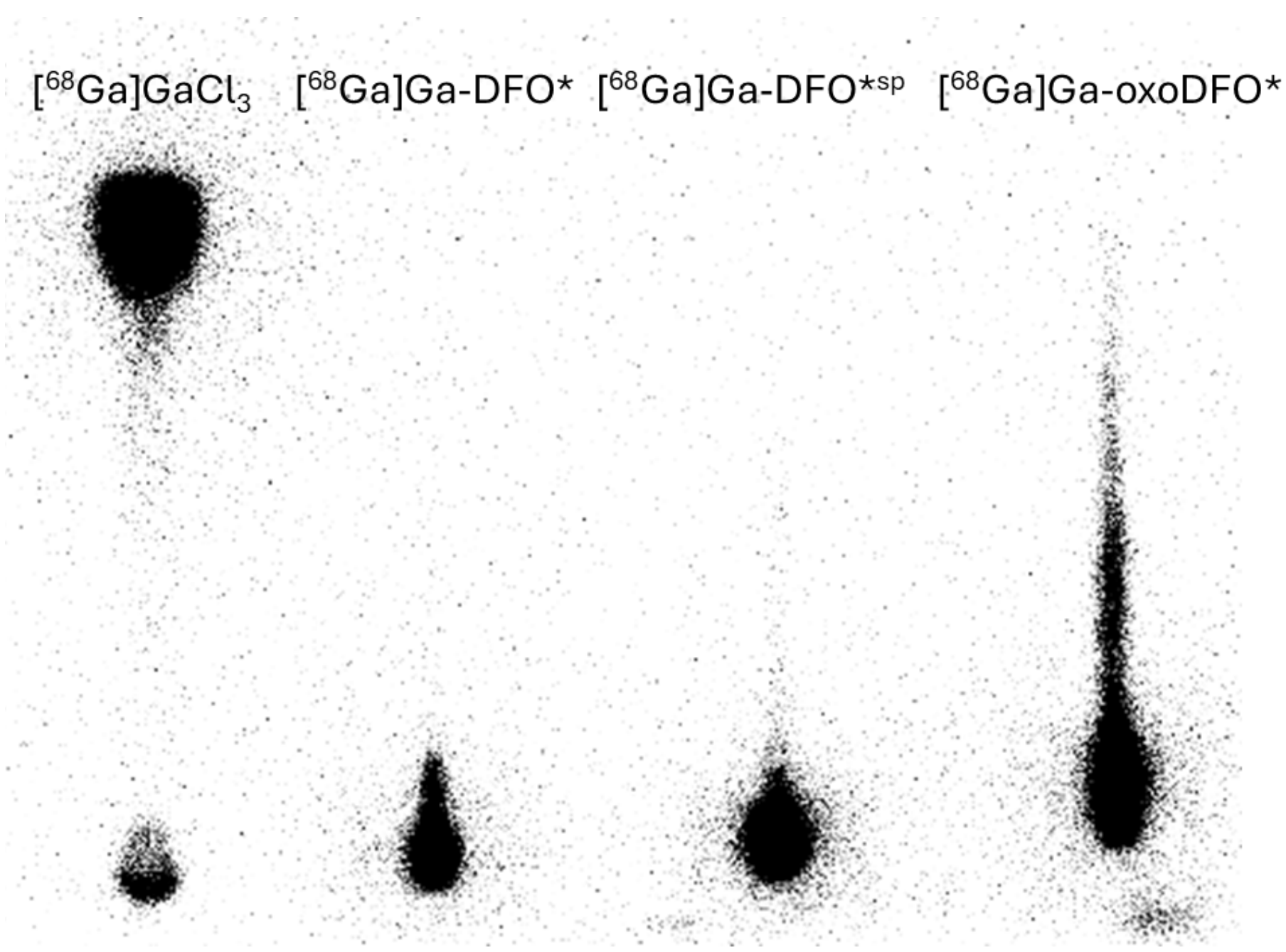

Figure S50: TLC radiochromatogram (mobile phase – citrate buffer, 0.1M, pH 5): the radiochemical purities from left: 95,4 %; 99,9 %; 99,9 %; 99,3%

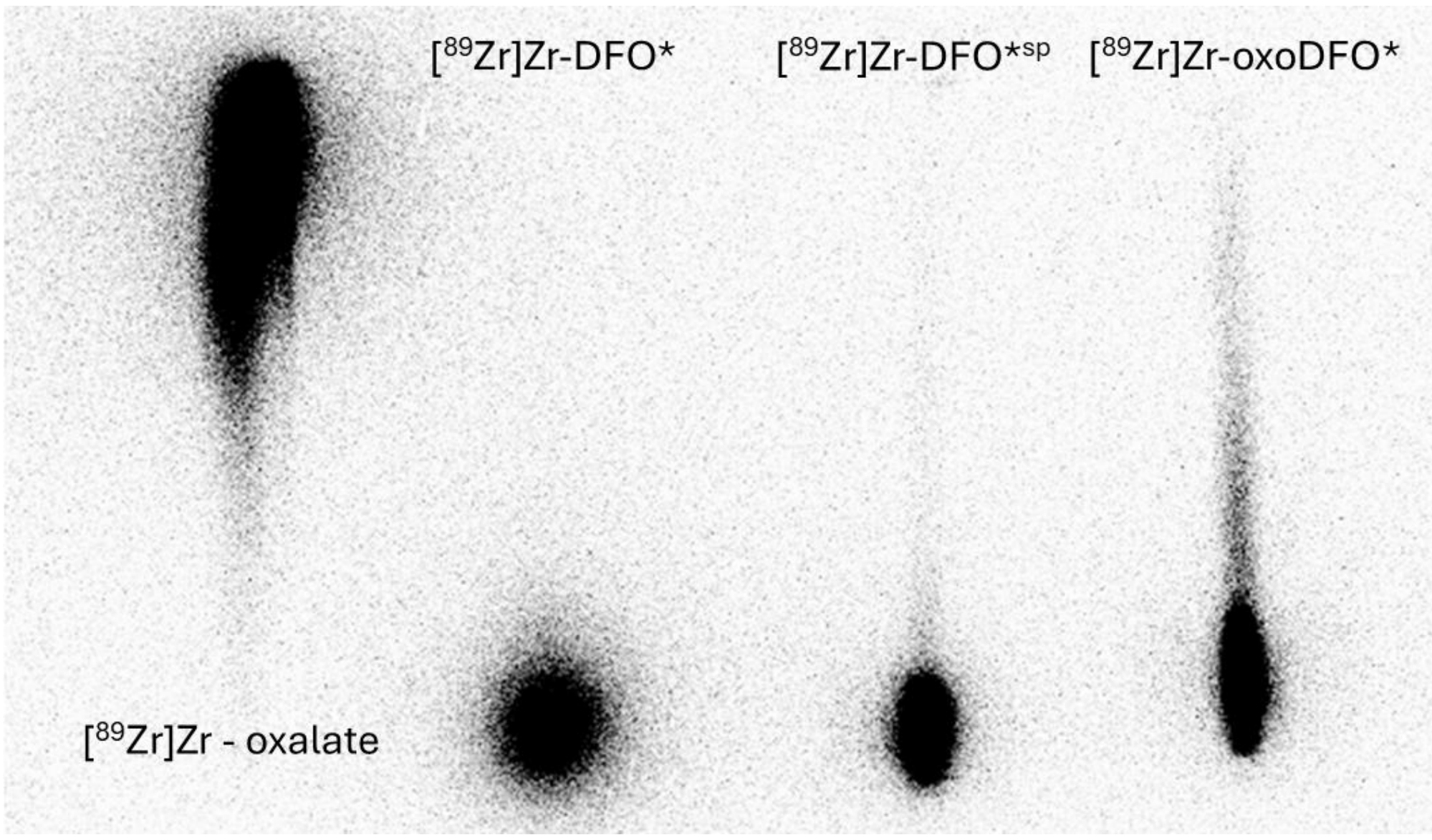

Figure S51: TLC radiochromatogram (mobile phase – citrate buffer, 0.1 M, pH 5): the radiochemical purities from left: 99,9 %; 99,9 %; 99,9 %; 98,9 %

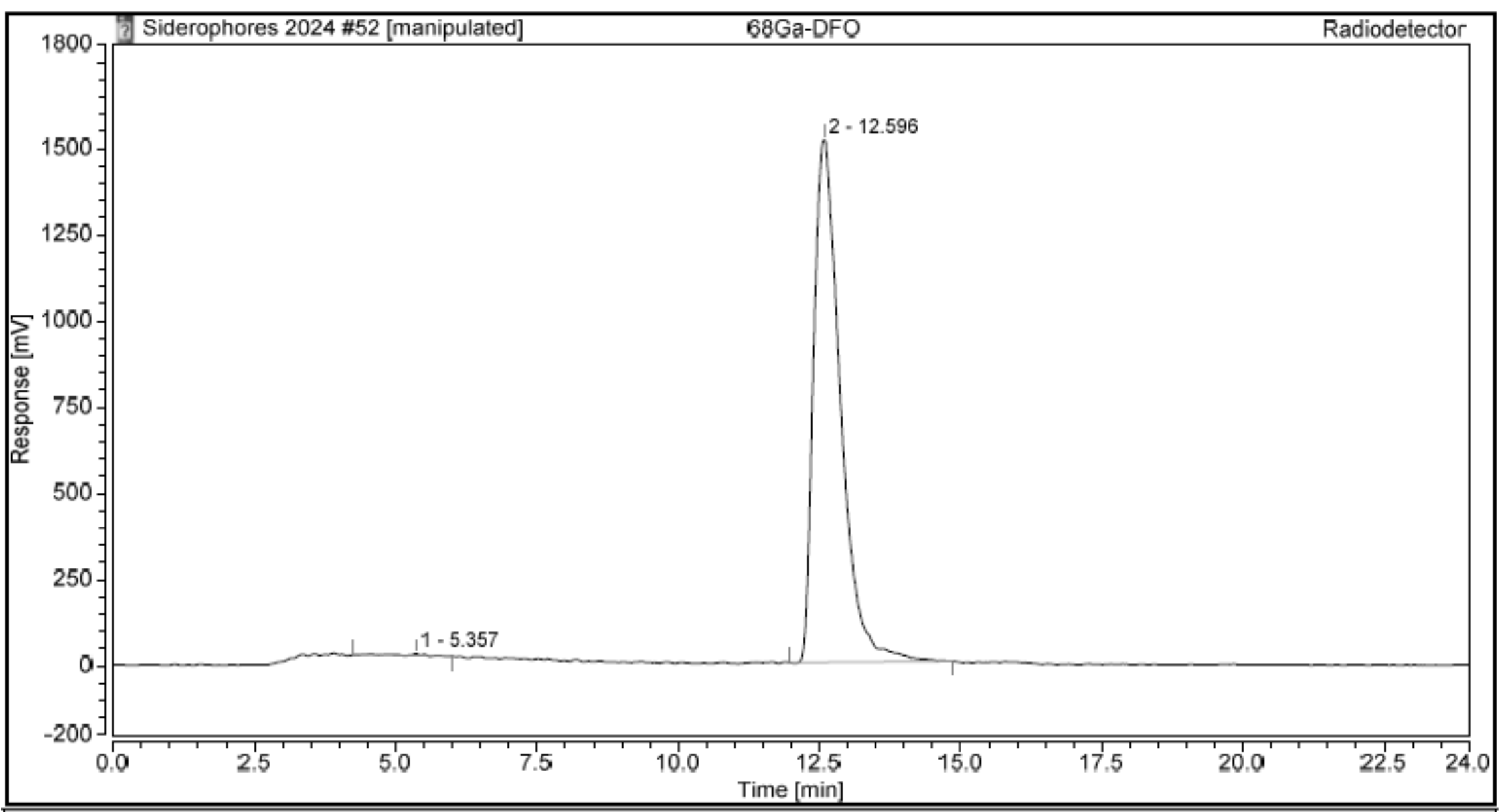

Integration Results

No.	Peak Name	Retention Time min	Area mV*min	Height mV	Relative Area %	Relative Height %	Amount n.a.
1		5.357	1.923	6.662	0.23	0.44	n.a.
2		12.596	830.941	1516.521	99.77	99.56	n.a.
**Total:**			**832.864**	**1523.183**	**100.00**	**100.00**	

*Figure S52: HPLC radiochromatogram of [^{68}Ga]Ga-DFO **5**.*

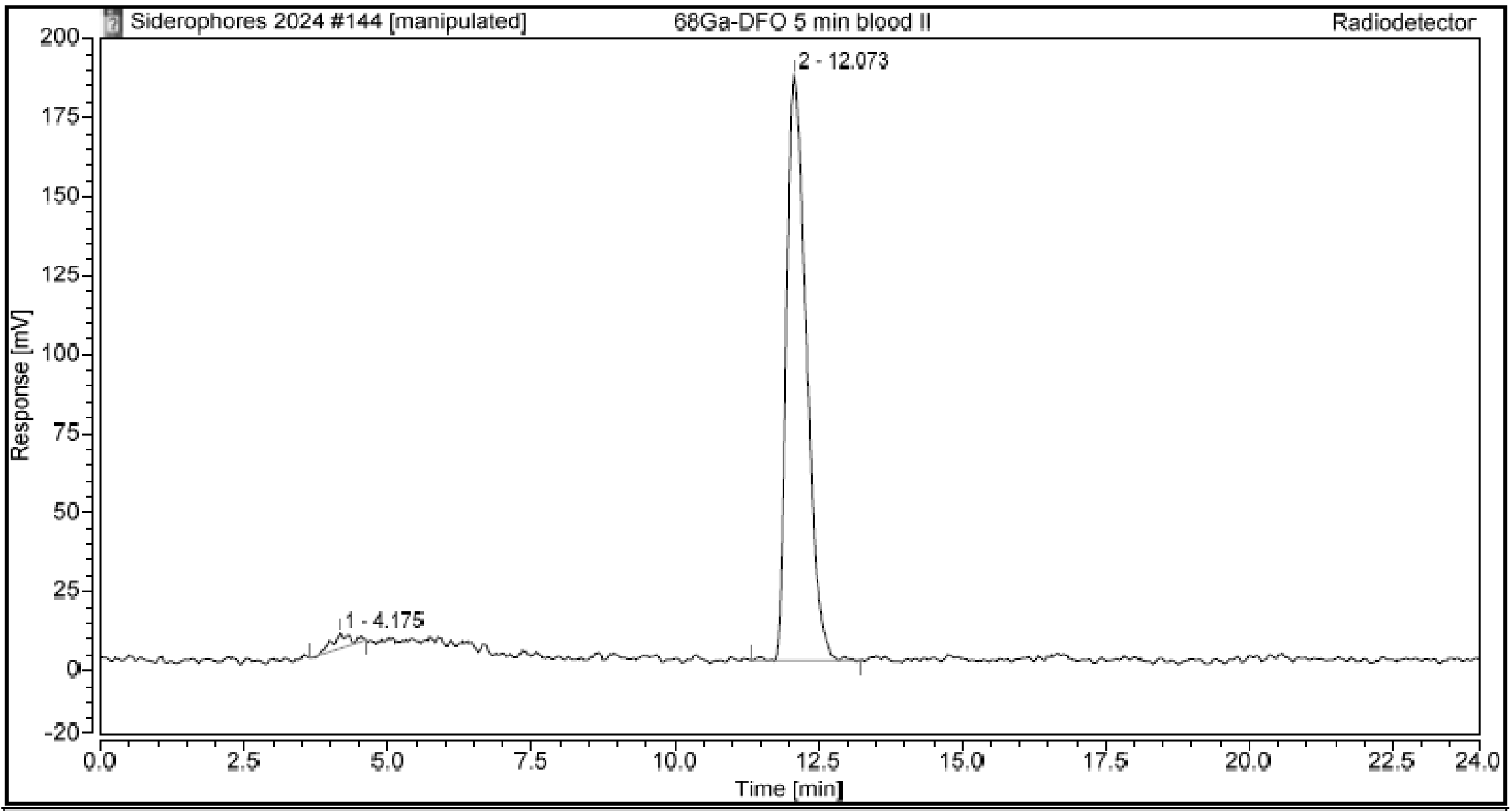

Integration Results

No.	Peak Name	Retention Time<br>min	Area<br>mV*min	Height<br>mV	Relative Area<br>%	Relative Height<br>%	Amount<br>n.a.
1		4.175	1.656	4.871	2.19	2.56	n.a.
2		12.073	74.022	185.632	97.81	97.44	n.a.
Total:			75.678	190.502	100.00	100.00	

*Figure S53: Stability of [^{68}Ga]Ga-DFO **5** in blood after 5 min.*

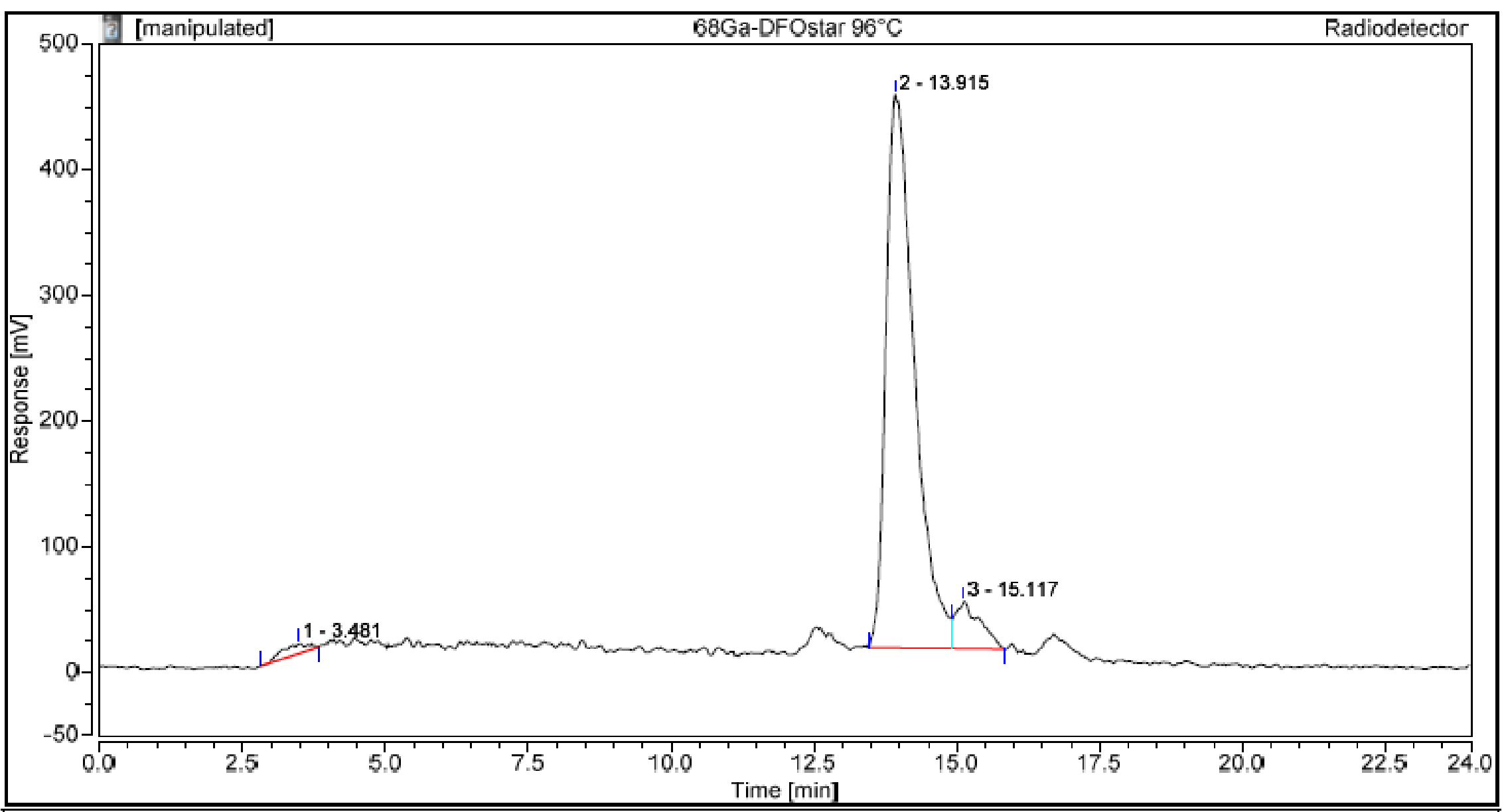

Integration Results

No.	Peak Name	Retention Time<br>min	Area<br>mV*min	Height<br>mV	Relative Area<br>%	Relative Height<br>%	Amount<br>n.a.
1		3.481	4.459	8.236	1.61	1.70	n.a.
2		13.915	253.984	439.890	91.53	90.58	n.a.
3		15.117	19.047	37.492	6.86	7.72	n.a.
Total:			277.489	485.618	100.00	100.00	

Figure S54: HPLC radiochromatogram of [^{68}Ga]Ga-DFO **6**.*

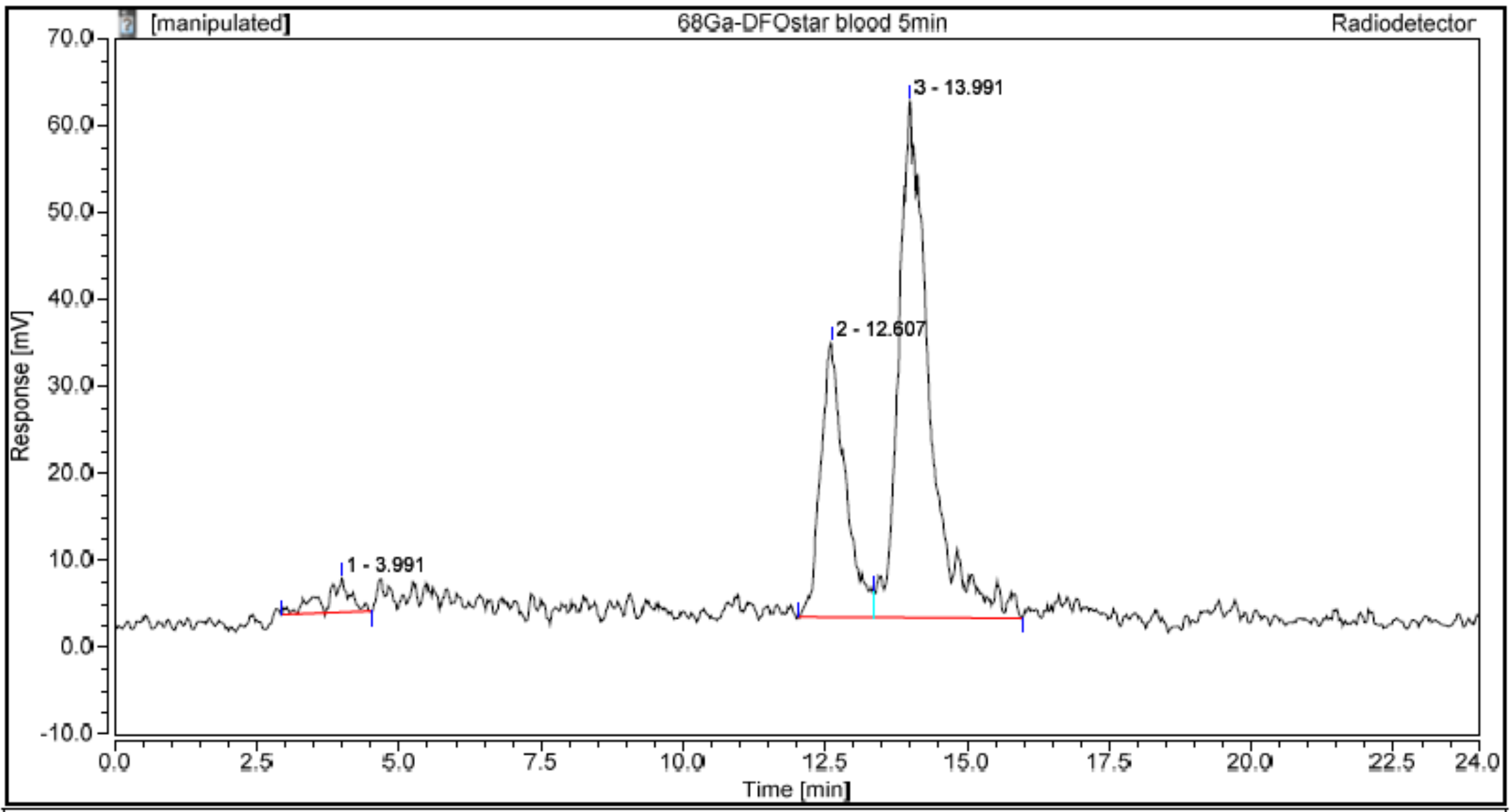

Integration Results

No.	Peak Name	Retention Time min	Area mV*min	Height mV	Relative Area %	Relative Height %	Amount n.a.
1		3.991	2.063	3.940	3.71	4.14	n.a.
2		12.607	15.839	31.730	28.46	33.33	n.a.
3		13.991	37.759	59.541	67.84	62.54	n.a.
**Total:**			**55.661**	**95.211**	**100.00**	**100.00**	

Figure S55: Stability of [^{68}Ga]Ga-DFO **6** in blood after 5 min.*

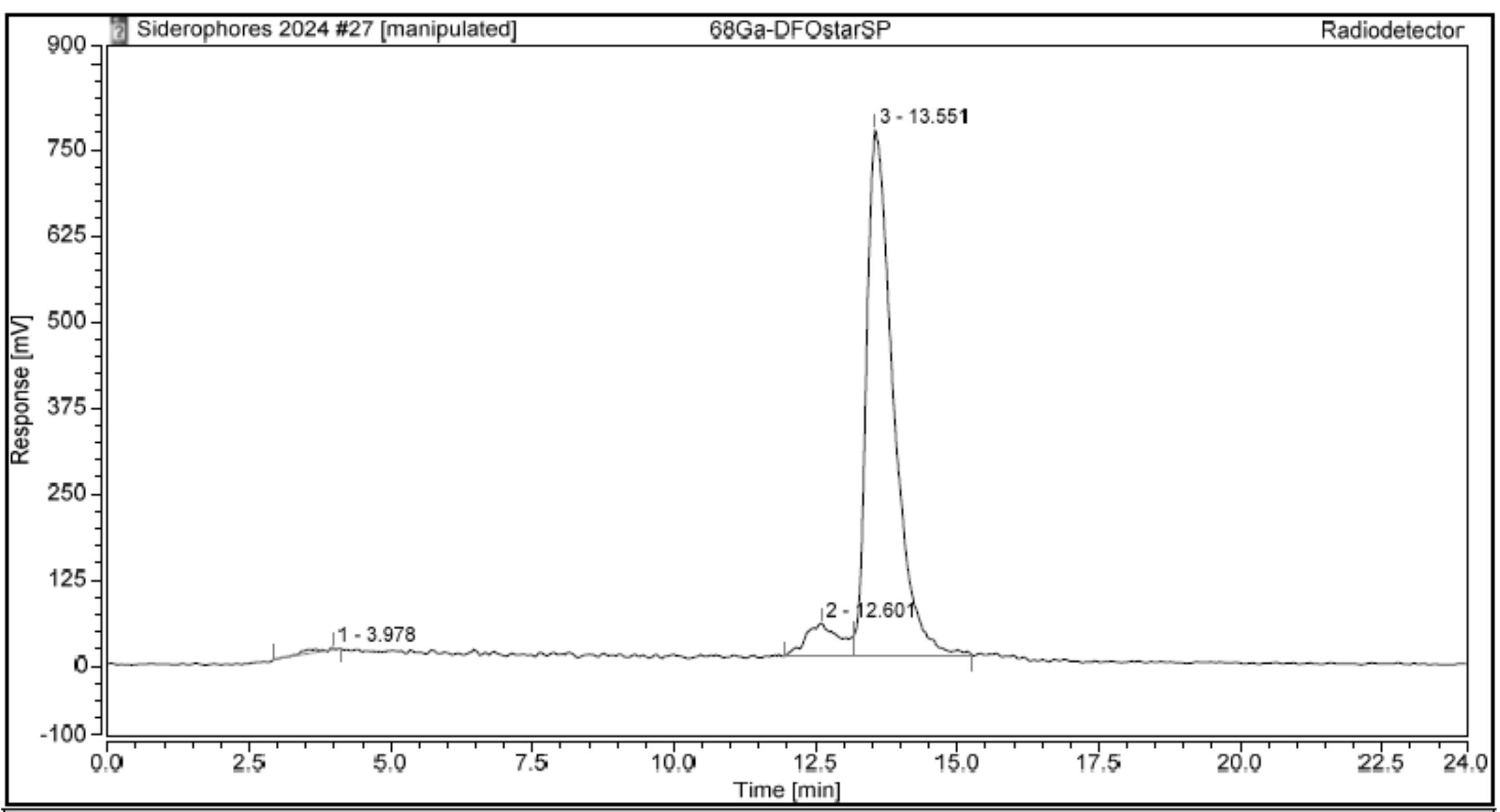

Integration Results

No.	Peak Name	Retention Time min	Area mV*min	Height mV	Relative Area %	Relative Height %	Amount n.a.
1		3.978	2.246	3.569	0.49	0.44	n.a.
2		12.601	33.573	49.001	7.37	5.99	n.a.
3		13.551	419.522	764.925	92.13	93.57	n.a.
**Total:**			**455.341**	**817.495**	**100.00**	**100.00**	

Figure S56: HPLC radiochromatogram of [^{68}Ga]Ga-DFO^{sp} **7**.*

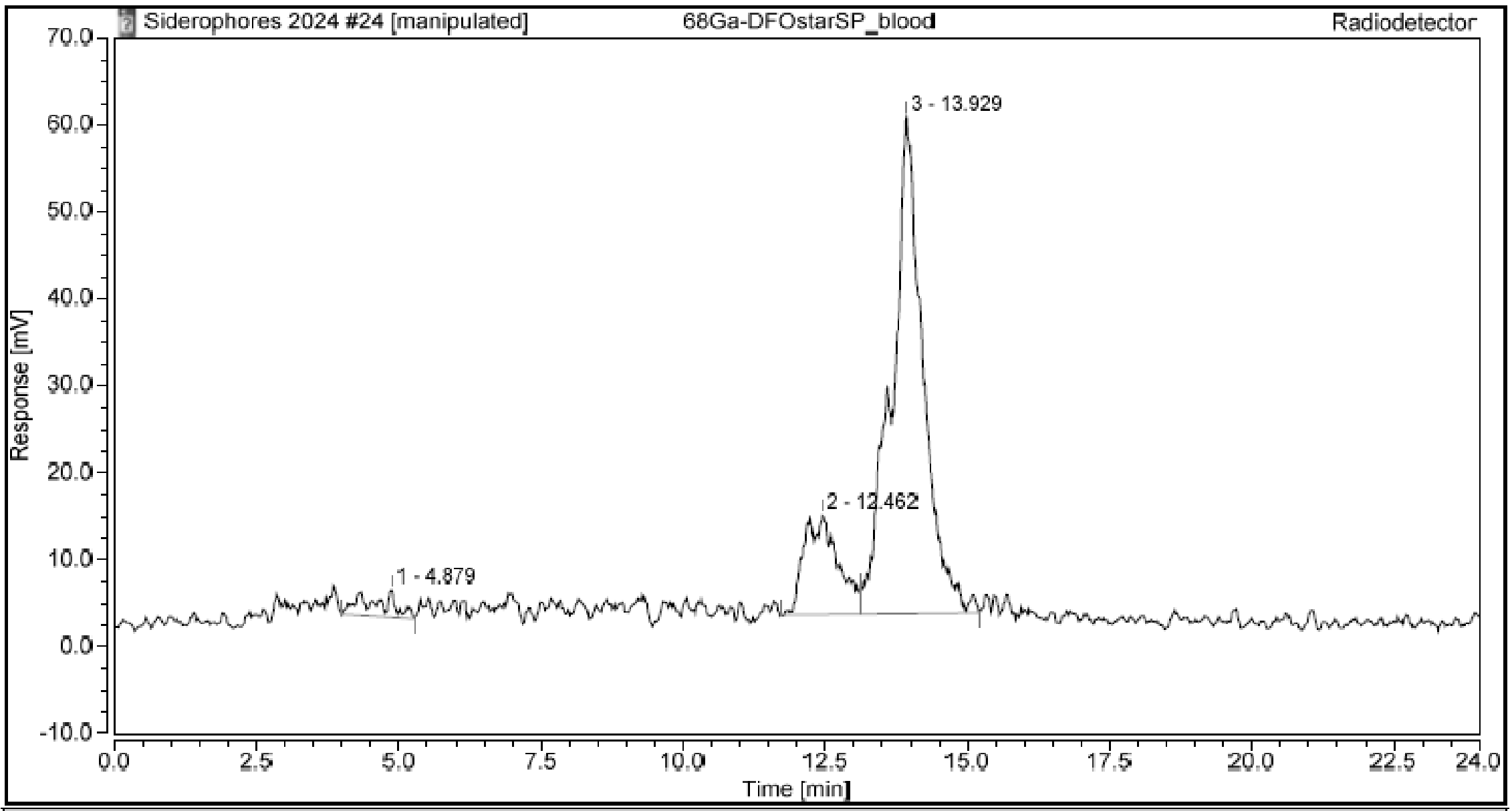

Integration Results

No.	Peak Name	Retention Time min	Area mV*min	Height mV	Relative Area %	Relative Height %	Amount n.a.
1		4.879	1.653	3.237	3.56	4.49	n.a.
2		12.462	7.947	11.587	17.11	16.07	n.a.
3		13.929	36.858	57.295	79.34	79.45	n.a.
Total:			**46.458**	**72.119**	**100.00**	**100.00**	

Figure S57: Stability of [^{68}Ga]Ga-DFO^{sp} **7** in blood after 5 min.*

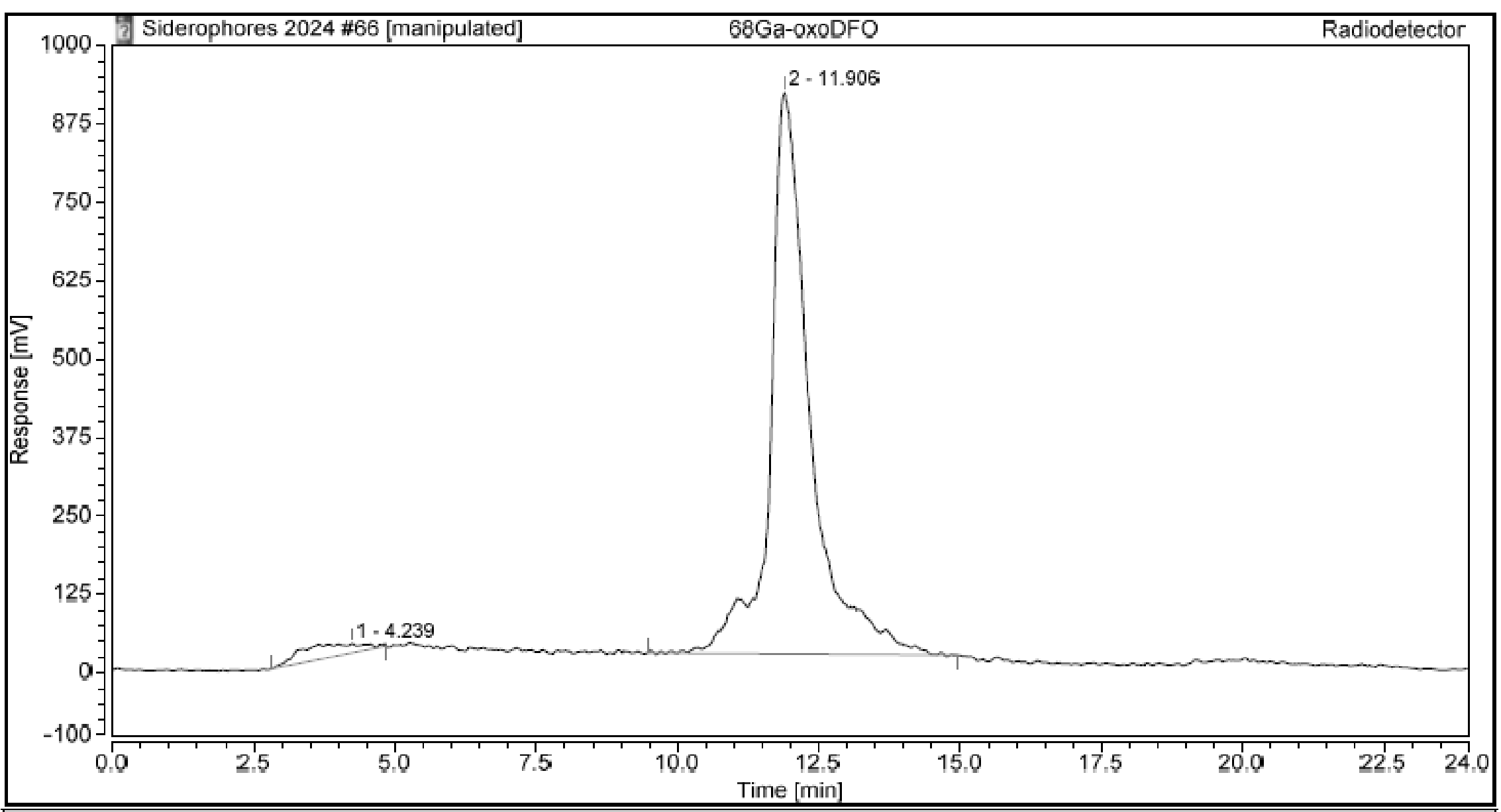

Integration Results

No.	Peak Name	Retention Time min	Area mV*min	Height mV	Relative Area %	Relative Height %	Amount n.a.
1		4.239	25.870	15.772	3.41	1.73	n.a.
2		11.906	732.997	897.123	96.59	98.27	n.a.
Total:			**758.868**	**912.895**	**100.00**	**100.00**	

Figure S58: HPLC radiochromatogram of [^{68}Ga]Ga-oxoDFO **8**.*

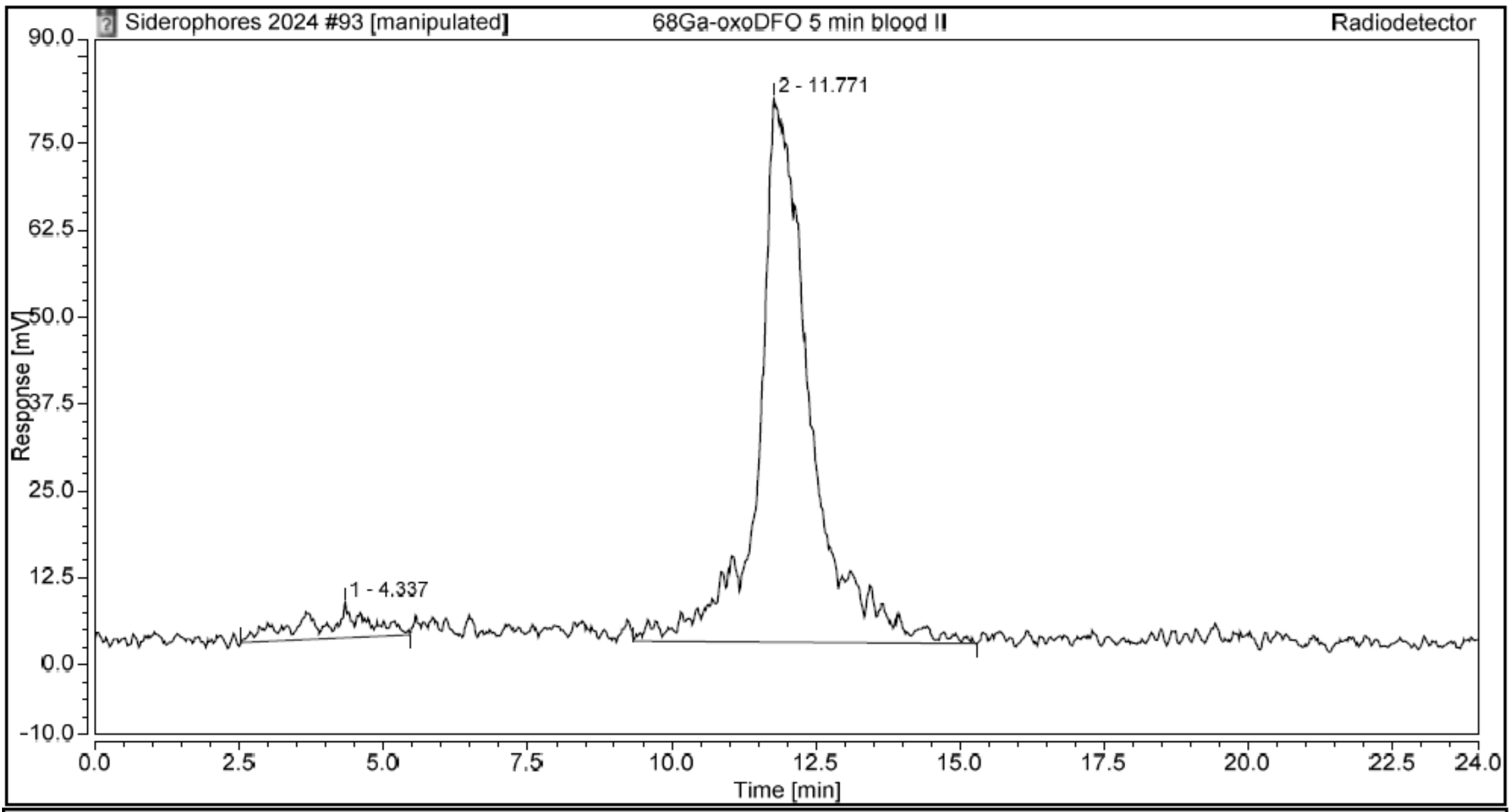

Integration Results

No.	Peak Name	Retention Time min	Area mV*min	Height mV	Relative Area %	Relative Height %	Amount n.a.
1		4.337	5.312	5.092	6.07	6.11	n.a.
2		11.771	82.239	78.247	93.93	93.89	n.a.
**Total:**			**87.550**	**83.340**	**100.00**	**100.00**	

Figure S59: Stability of [^{68}Ga]Ga-oxoDFO **8** in blood after 5 min.*

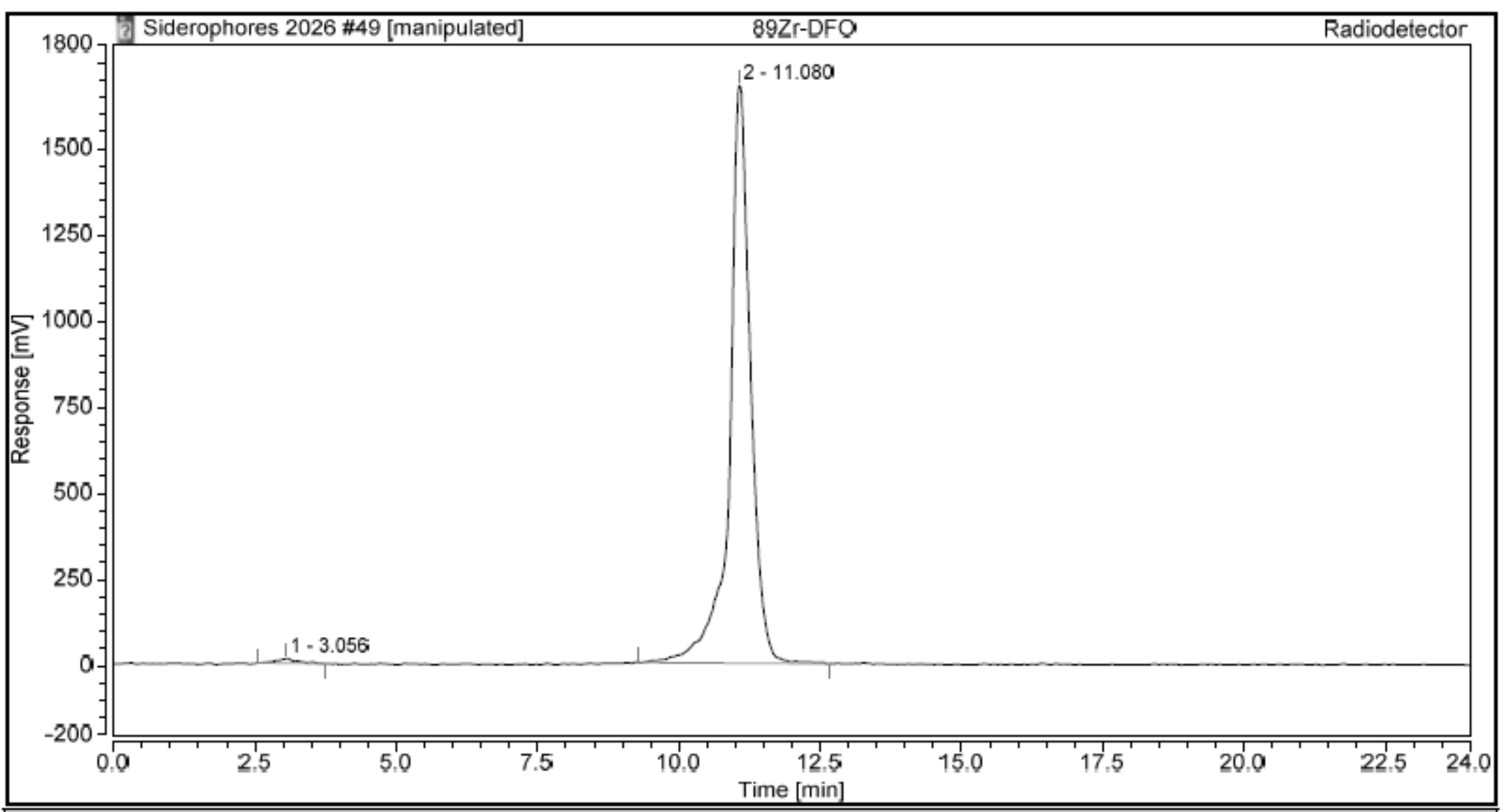

Integration Results

No.	Peak Name	Retention Time min	Area mV*min	Height mV	Relative Area %	Relative Height %	Amount n.a.
1		3.056	5.880	13.910	0.81	0.82	n.a.
2		11.080	723.086	1677.165	99.19	99.18	n.a.
**Total:**			**728.966**	**1691.076**	**100.00**	**100.00**	

*Figure S60: HPLC radiochromatogram of [^{89}Zr]Zr-DFO **9**.*

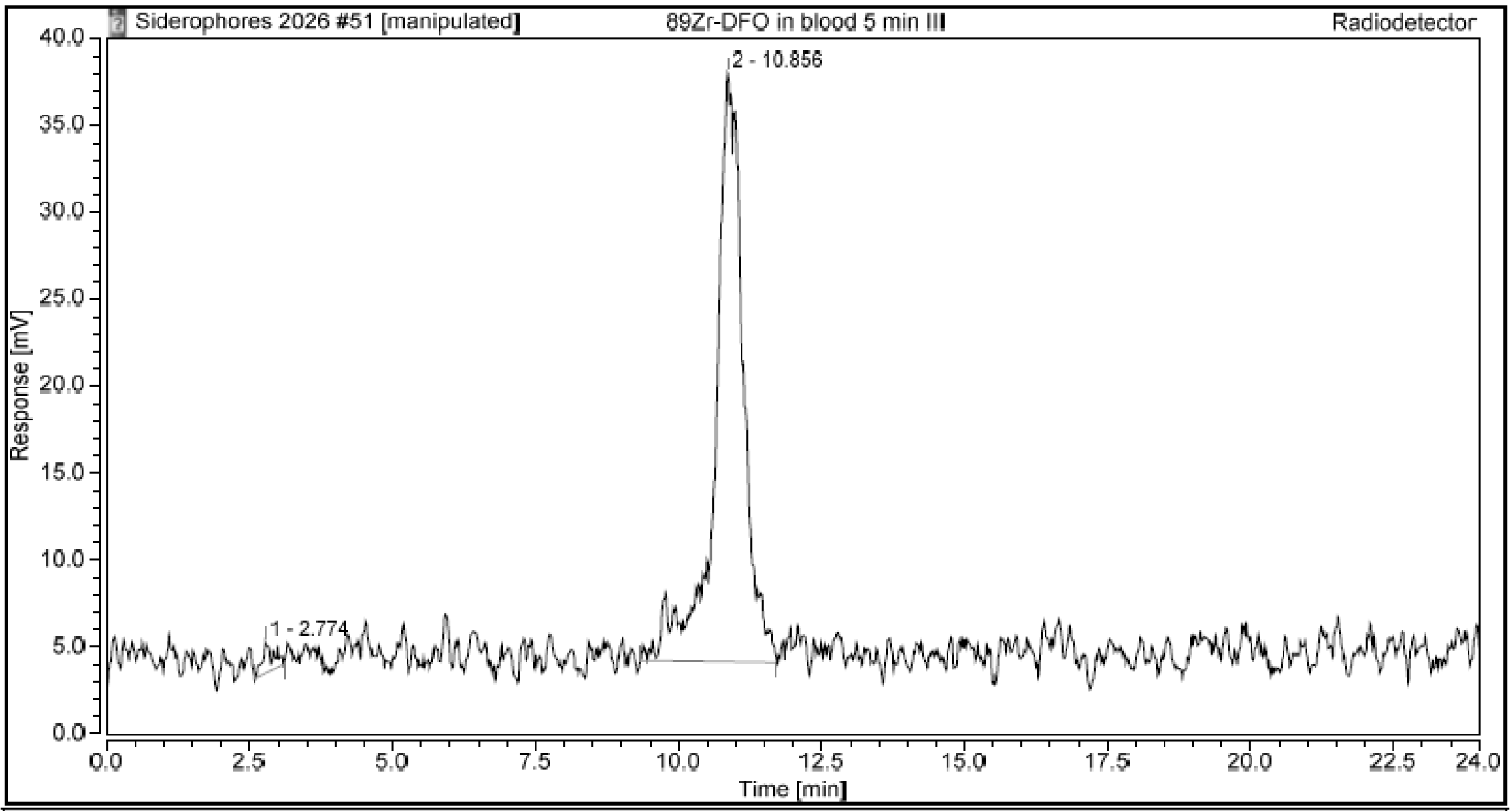

Integration Results

No.	Peak Name	Retention Time min	Area mV*min	Height mV	Relative Area %	Relative Height %	Amount n.a.
1		2.774	0.383	1.818	1.93	5.09	n.a.
2		10.856	19.480	33.919	98.07	94.91	n.a.
**Total:**			**19.863**	**35.737**	**100.00**	**100.00**	

*Figure S61: Stability of [^{89}Zr]Zr-DFO **9** in blood after 5 min.*

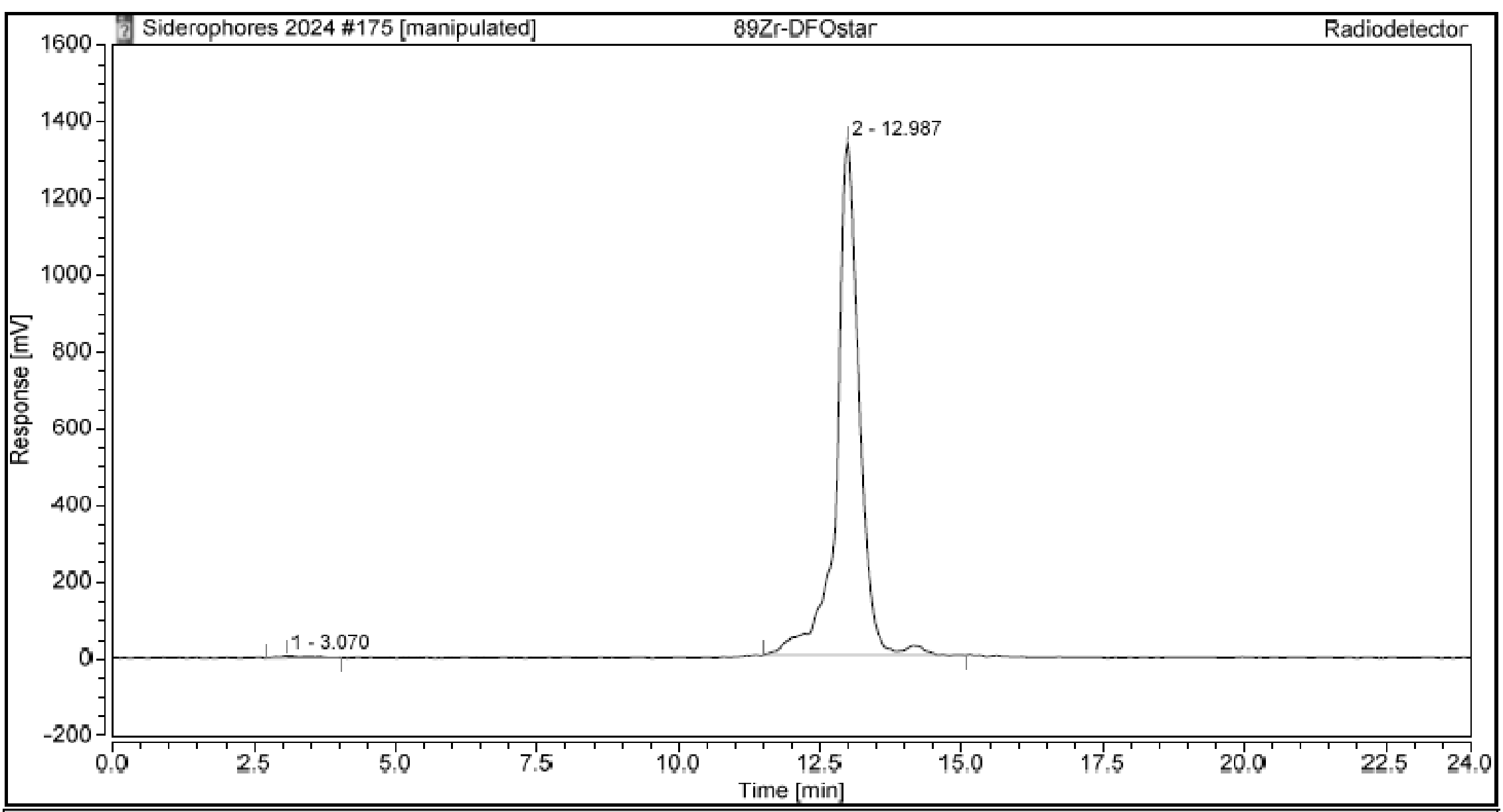

Integration Results

No.	Peak Name	Retention Time min	Area mV*min	Height mV	Relative Area %	Relative Height %	Amount n.a.
1		3.070	3.928	6.533	0.62	0.49	n.a.
2		12.987	626.006	1338.582	99.38	99.51	n.a.
**Total:**			**629.934**	**1345.114**	**100.00**	**100.00**	

Figure S62: HPLC radiochromatogram of [^{89}Zr]Zr-DFO **10**.*

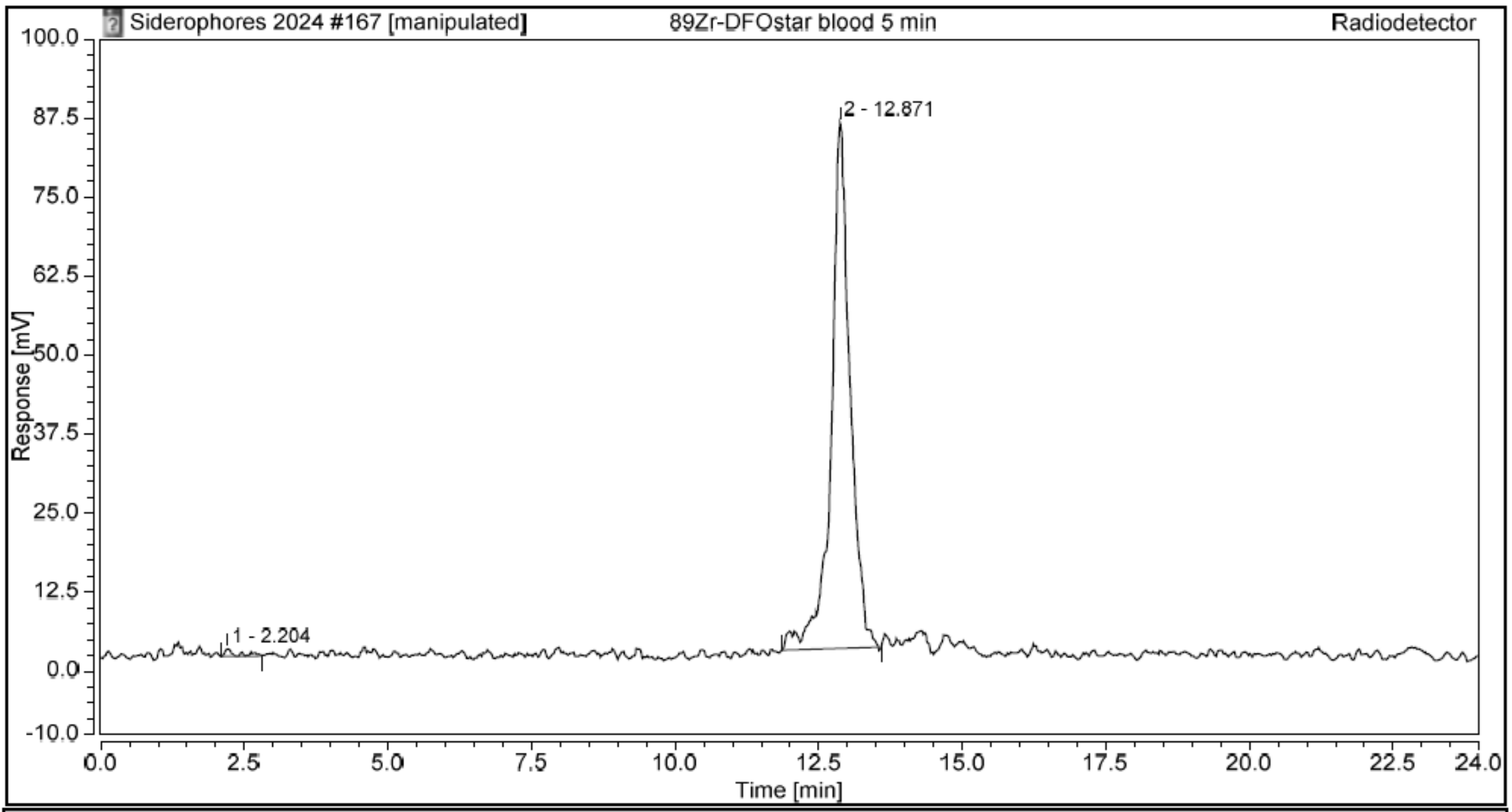

Integration Results							
No.	Peak Name	Retention Time min	Area mV*min	Height mV	Relative Area %	Relative Height %	Amount n.a.
1		2.204	0.280	1.307	0.87	1.54	n.a.
2		12.871	31.901	83.374	99.13	98.46	n.a.
**Total:**			**32.181**	**84.680**	**100.00**	**100.00**	

Figure S63: Stability of [^{89}Zr]Zr-DFO **10** in blood after 5 min.*

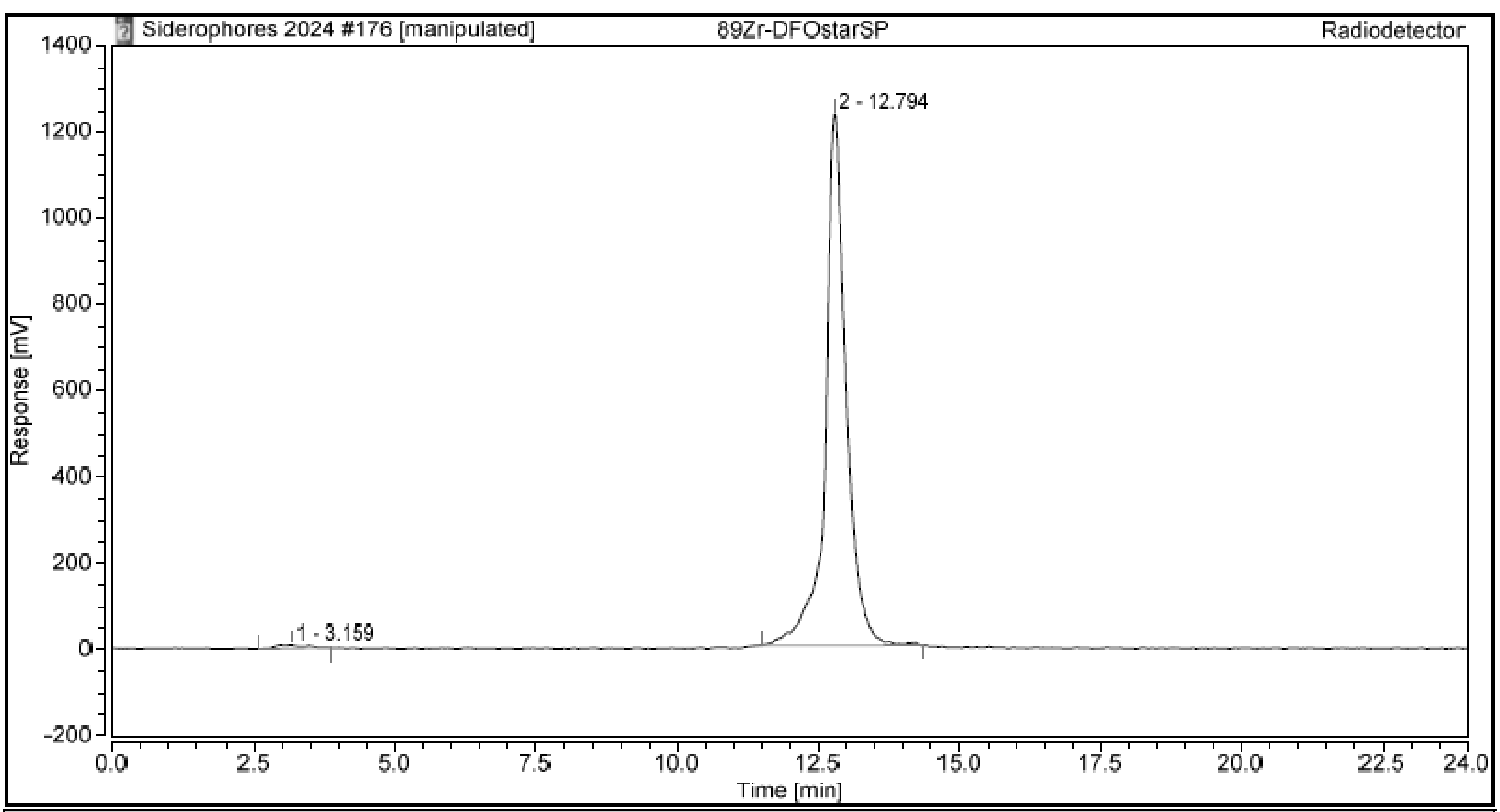

Integration Results							
No.	Peak Name	Retention Time min	Area mV*min	Height mV	Relative Area %	Relative Height %	Amount n.a.
1		3.159	4.444	7.758	0.83	0.63	n.a.
2		12.794	532.495	1232.603	99.17	99.37	n.a.
**Total:**			**536.939**	**1240.361**	**100.00**	**100.00**	

Figure S64: HPLC radiochromatogram of [^{89}Zr]Zr-DFO^{sp} **11**.*

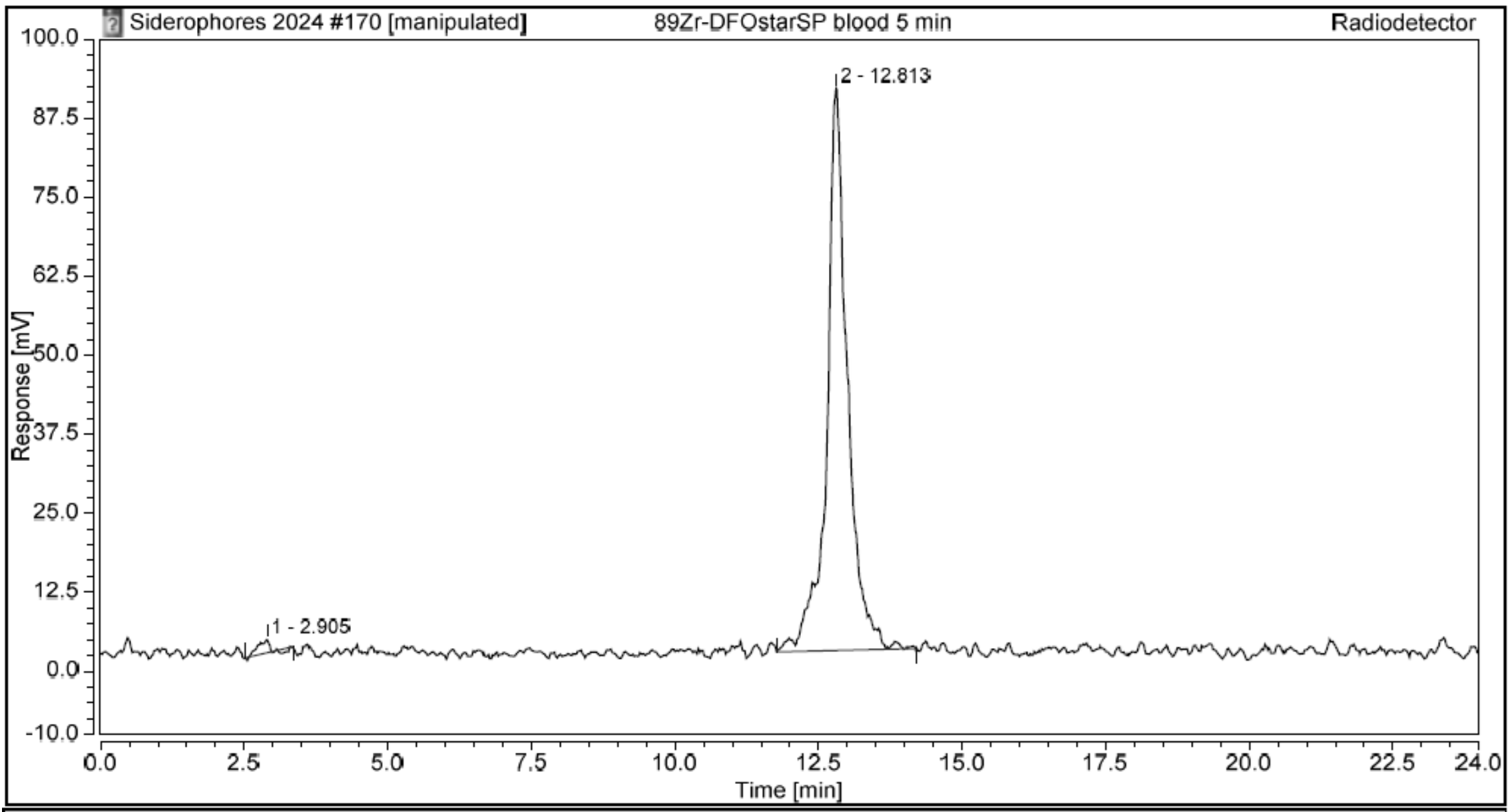

Integration Results							
No.	Peak Name	Retention Time min	Area mV*min	Height mV	Relative Area %	Relative Height %	Amount n.a.
1		2.905	0.352	2.181	0.93	2.39	n.a.
2		12.813	37.319	88.941	99.07	97.61	n.a.
**Total:**			**37.671**	**91.122**	**100.00**	**100.00**	

Figure S65: Stability of [^{89}Zr]Zr-DFO^{sp} **11** in blood after 5 min.*

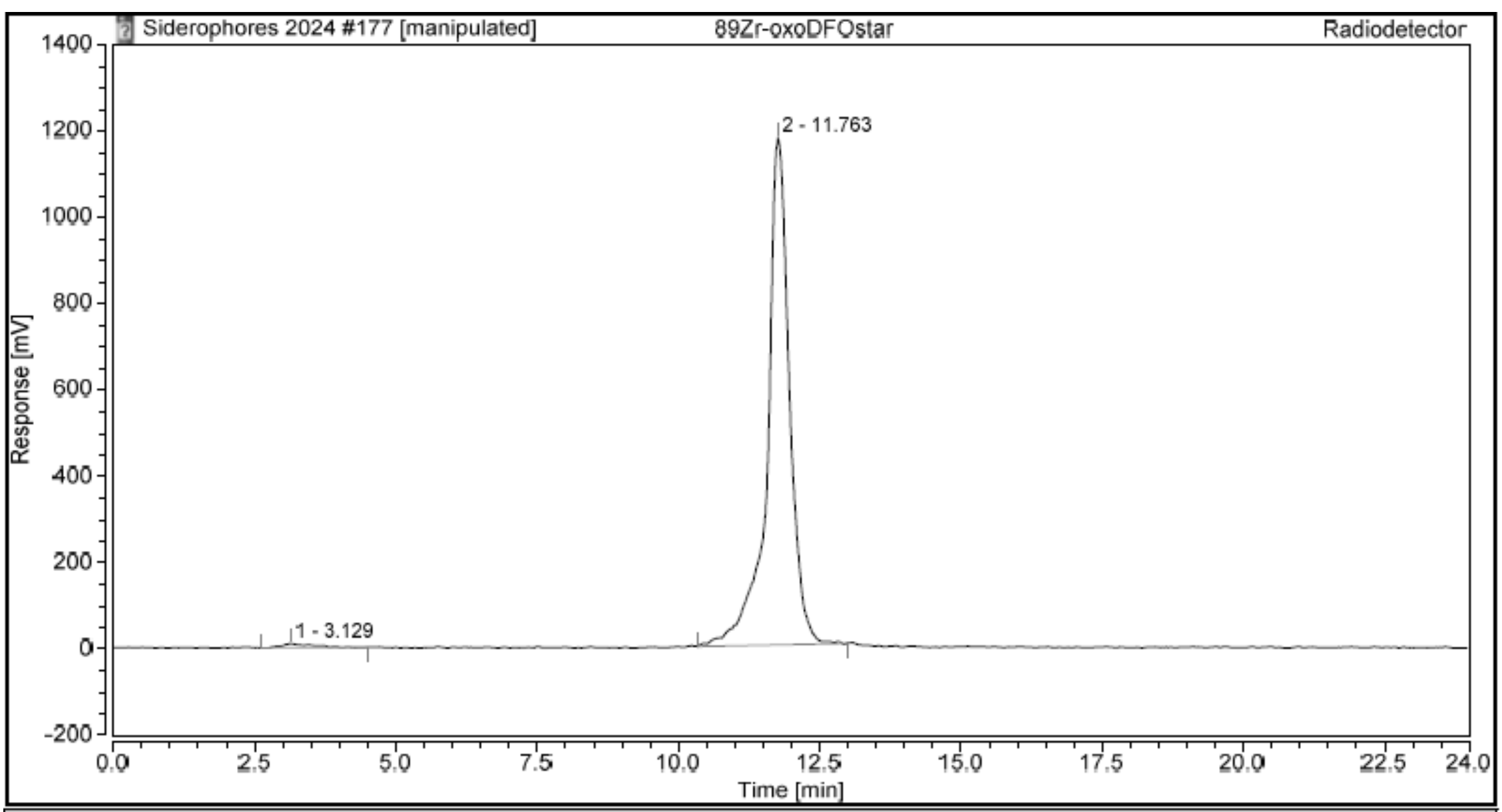

Integration Results							
No.	Peak Name	Retention Time min	Area mV*min	Height mV	Relative Area %	Relative Height %	Amount n.a.
1		3.129	5.807	10.248	1.07	0.86	n.a.
2		11.763	538.500	1176.153	98.93	99.14	n.a.
**Total:**			**544.307**	**1186.401**	**100.00**	**100.00**	

Figure S66: HPLC radiochromatogram of [^{89}Zr]Zr-oxoDFO **12**.*

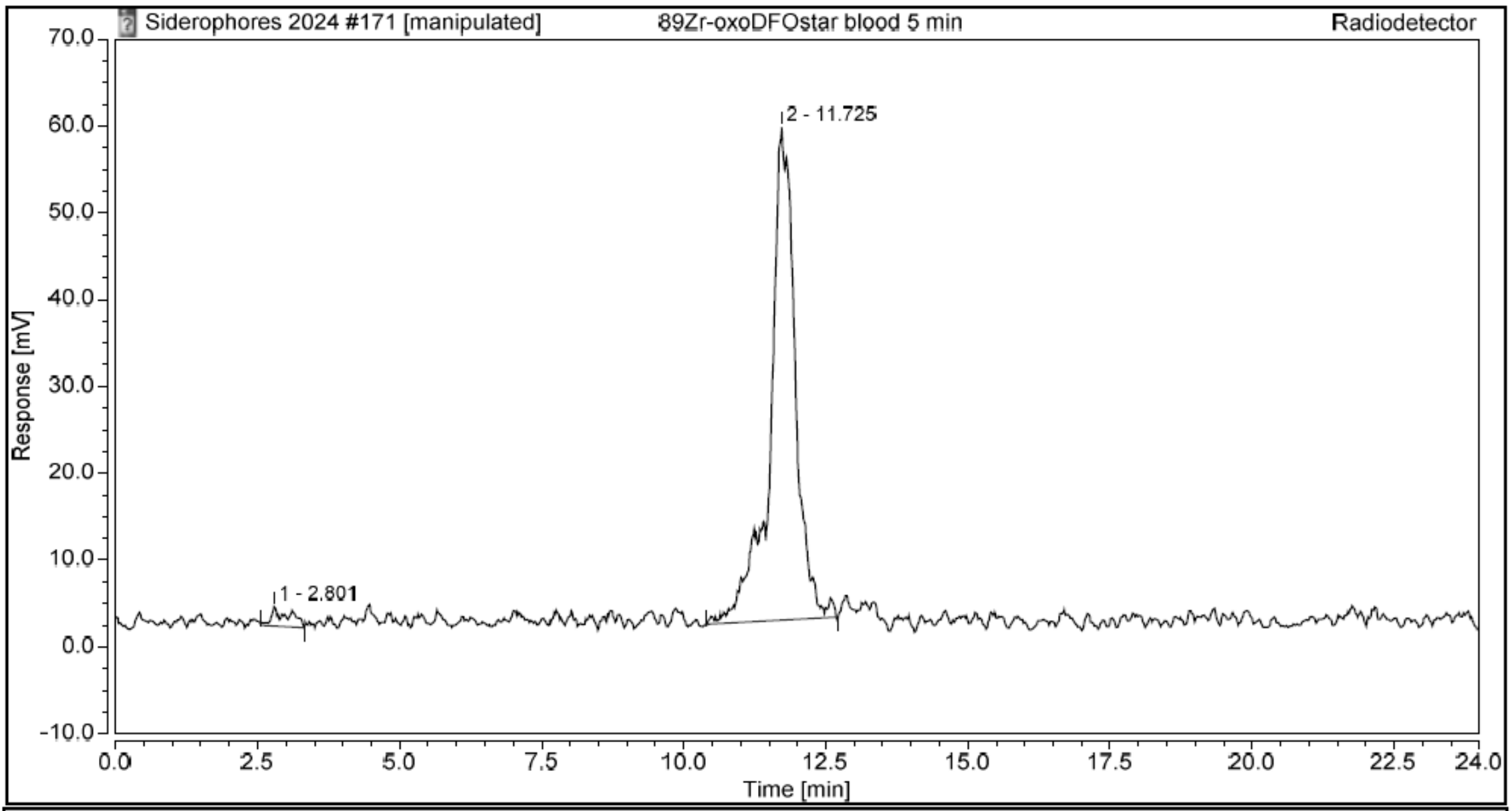

Integration Results							
No.	Peak Name	Retention Time min	Area mV*min	Height mV	Relative Area %	Relative Height %	Amount n.a.
1		2.801	0.794	2.255	2.64	3.82	n.a.
2		11.725	29.265	56.841	97.36	96.18	n.a.
**Total:**			**30.059**	**59.097**	**100.00**	**100.00**	

Figure S67: Stability of [89Zr]Zr-oxoDFO **12** in blood after 5 min.*

Ex vivo biodistribution study of [^{68}Ga]Ga- and [^{89}Zr]Zr-DFO-analogs 5, 6, 7, 8, 9, 10, 11, and 12.

*Table S3: Ex vivo biodistribution of [^{68}Ga]Ga-DFO **5**.*

	0.5 h		1.5 h	
	MV	*sd*	MV	*sd*
blood	1.24	0.11	0.04	0.01
spleen	0.39	0.01	0.07	0.01
pancreas	0.35	0.02	0.03	0.003
stomach	0.63	0.07	0.07	0.01
intestine	0.43	0.01	0.18	0.04
kidneys	5.11	0.28	2.19	0.21
liver	0.43	0.01	0.09	0.02
heart	0.54	0.02	0.04	0.002
lung	1.21	0.10	0.13	0.03
muscle	0.26	0.04	0.02	0.01
femur	0.24	0.05	0.05	0.01

Table S4: Ex vivo biodistribution of [^{68}Ga]Ga-DFO **6**.*

	0.5 h		1.5 h	
	MV	*sd*	MV	*sd*
blood	0.96	0.18	0.09	0.03
spleen	0.33	0.06	0.07	0.01
pancreas	0.28	0.06	0.04	0.01
stomach	0.59	0.13	0.14	0.05
intestine	1.12	0.08	1.29	0.38
kidneys	6.57	0.56	4.34	0.43
liver	0.66	0.06	0.17	0.06
heart	0.41	0.08	0.05	0.02
lung	1.15	0.12	0.21	0.08
muscle	0.18	0.05	0.02	0.01
femur	0.17	0.04	0.05	0.01

*Table S5: Ex vivo biodistribution of [^{68}Ga]Ga-DFO^{*sp} **7**.*

	0.5 h		1.5 h	
	MV	*sd*	MV	*sd*
blood	0.59	0.13	0.04	0.001
spleen	0.24	0.05	0.11	0.02
pancreas	0.19	0.05	0.03	0.003
stomach	0.41	0.12	0.27	0.20
intestine	0.54	0.10	0.51	0.04
kidneys	4.73	0.60	3.91	0.39
liver	0.41	0.04	0.15	0.03
heart	0.26	0.05	0.05	0.003
lung	0.89	0.20	0.15	0.01
muscle	0.13	0.02	0.02	0.002
femur	0.18	0.02	0.07	0.02

Table S6: Ex vivo biodistribution of [68Ga]Ga-oxoDFO **8**.*

	0.5 h		1.5 h	
	MV	*sd*	MV	*sd*
blood	1.07	0.10	0.11	0.003
spleen	0.33	0.03	0.10	0.01
pancreas	0.31	0.01	0.05	0.01
stomach	0.68	0.02	0.11	0.03

intestine	0.48	0.01	0.22	0.08
kidneys	5.63	0.97	3.30	0.45
liver	0.39	0.04	0.12	0.01
heart	0.41	0.06	0.06	0.002
lung	1.07	0.02	0.22	0.02
muscle	0.19	0.03	0.02	0.004
femur	0.17	0.02	0.05	0.01

*Table S7: Ex vivo biodistribution of [^{89}Zr]Zr-DFO **9**.*

	1 h		3 h		24 h	
	MV	*sd*	MV	*sd*	MV	*sd*
blood	0.15	0.02	0.02	0.003	0.01	0.001
spleen	0.10	0.02	0.06	0.01	0.06	0.002
pancreas	0.07	0.01	0.02	0.004	0.02	0.0003
stomach	0.12	0.04	0.08	0.04	0.06	0.03
intestine	0.21	0.04	0.25	0.03	0.19	0.11
kidneys	2.76	0.14	2.62	0.51	1.84	0.36
liver	0.43	0.06	0.21	0.03	0.11	0.01
heart	0.11	0.02	0.03	0.01	0.03	0.002
lung	0.23	0.02	0.06	0.02	0.03	0.01
muscle	0.06	0.01	0.02	0.004	0.01	0.002
femur	0.08	0.01	0.03	0.01	0.06	0.01

Table S8: Ex vivo biodistribution [^{89}Zr]Zr-DFO **10**.*

	1 h		3 h		24 h	
	MV	*sd*	MV	*sd*	MV	*sd*
blood	*0.11*	0.02	*0.01*	0.001	*0.01*	0.004
spleen	*0.14*	0.02	*0.10*	0.01	*0.06*	0.02
pancreas	*0.07*	0.01	*0.03*	0.01	*0.02*	0.005
stomach	*0.44*	0.06	*0.52*	0.26	*0.21*	0.23
intestine	*1.15*	0.10	*1.39*	0.23	*0.14*	0.12
kidneys	*3.68*	0.49	*2.98*	0.16	*2.14*	0.20
liver	*0.34*	0.04	*0.18*	0.02	*0.07*	0.01
heart	*0.10*	0.01	*0.05*	0.003	*0.03*	0.01
lung	*0.24*	0.01	*0.09*	0.02	*0.05*	0.003
muscle	*0.07*	0.01	*0.02*	0.003	*0.02*	0.01
femur	*0.14*	0.02	*0.08*	0.01	*0.11*	0.04

Table S9: Ex vivo biodistribution of [^{89}Zr]Zr-DFO^{sp} **11**.*

	1 h		3 h		24 h	
	MV	*sd*	MV	*sd*	MV	*sd*
blood	0.13	*0.02*	0.01	*0.002*	0.01	*0.001*
spleen	0.29	*0.09*	0.41	*0.09*	0.17	*0.08*
pancreas	0.09	*0.03*	0.05	*0.02*	0.04	*0.01*
stomach	0.16	*0.06*	0.46	*0.41*	0.12	*0.05*
intestine	0.24	*0.13*	0.31	*0.09*	0.57	*0.40*
kidneys	4.56	*0.47*	4.53	*0.50*	2.54	*0.07*
liver	0.30	*0.12*	0.26	*0.07*	0.12	*0.04*
heart	0.12	*0.02*	0.07	*0.002*	0.04	*0.002*
lung	0.30	*0.09*	0.19	*0.01*	0.07	*0.01*
muscle	0.15	*0.08*	0.37	*0.42*	0.03	*0.003*
femur	0.23	*0.04*	0.19	*0.05*	0.12	*0.003*

Table S10: Ex vivo biodistribution of [^{89}Zr]Zr-oxoDFO **12**.*

	1 h		3 h		24 h	
	MV	*sd*	MV	*sd*	MV	*sd*
blood	0.24	*0.13*	0.01	*0.002*	0.004	*0.001*
spleen	0.18	*0.06*	0.12	*0.02*	0.05	*0.003*
pancreas	0.39	*0.26*	0.07	*0.02*	0.02	*0.01*
stomach	0.27	*0.09*	0.40	*0.18*	0.03	*0.003*
intestine	0.16	*0.06*	0.23	*0.07*	0.03	*0.004*
kidneys	5.63	*1.13*	4.77	*0.24*	3.51	*0.47*
liver	0.17	*0.05*	0.10	*0.01*	0.07	*0.01*
heart	0.20	*0.09*	0.07	*0.01*	0.02	*0.01*
lung	0.36	*0.16*	0.09	*0.03*	0.04	*0.01*
muscle	0.10	*0.03*	0.06	*0.01*	0.01	*0.001*
femur	0.37	*0.04*	0.26	*0.02*	0.05	*0.01*
